# Design and Initial Results of a Multi-Phase Randomized Trial of Ceftriaxone in Amyotrophic Lateral Sclerosis

**DOI:** 10.1371/journal.pone.0061177

**Published:** 2013-04-17

**Authors:** James D. Berry, Jeremy M. Shefner, Robin Conwit, David Schoenfeld, Myles Keroack, Donna Felsenstein, Lisa Krivickas, William S. David, Francine Vriesendorp, Alan Pestronk, James B. Caress, Jonathan Katz, Ericka Simpson, Jeffrey Rosenfeld, Robert Pascuzzi, Jonathan Glass, Kourosh Rezania, Jeffrey D. Rothstein, David J. Greenblatt, Merit E. Cudkowicz, for the Northeast ALS Consortium

**Affiliations:** 1 Neurology Clinical Research Institute, Massachusetts General Hospital, Harvard Medical School, Boston, Massachusetts, United States of America; 2 Department of NeurologyState University of New York Upstate Medical University, Syracuse, New York, United States of America; 3 National Institute of Neurologic Disorders and Stroke, Bethesda, Maryland, United States of America; 4 Department of Biostatistics, Massachusetts General Hospital, Boston, Massachusetts, United States of America; 5 Department of Gastroenterology, Marshfield Clinic, Eau Claire, Wisconsin, United States of America; 6 Infectious Disease Unit/Department of Medicine, Massachusetts General Hospital, Harvard Medical School, Boston, Massachusetts, United States of America; 7 Department of Neurology, Washington University in St. Louis, St. Louis, Missouri, United States of America; 8 Department of Neurology, Wake Forest University, Winston-Salem, North Carolina, United States of America; 9 Department of Neurology, California Pacific Medical Center, San Francisco, California, United States of America; 10 Department of Neurology, Methodist Neurological Institute, Houston, Texas, United States of America; 11 Department of Neurology, University of California San Francisco Fresno, Neuroscience Institute, Fresno, California, United States of America; 12 Department of Neurology, Indiana University, Indianapolis, Indiana, United States of America; 13 Department of Neurology, Emory University, Atlanta, Georgia, United States of America; 14 Department of Neurology, University of Chicago, Chicago, Illinois, United States of America; 15 Department of Neurology, Johns Hopkins University, Baltimore, Maryland, United States of America; 16 Department of Pharmacology and Experimental Therapeutics, Tufts University School of Medicine, Boston, Massachusetts, United States of America; “Mario Negri” Institute for Pharmacological Research, Italy

## Abstract

**Objectives:**

Ceftriaxone increases expression of the astrocytic glutamate transporter, EAAT2, which might protect from glutamate-mediated excitotoxicity. A trial using a novel three stage nonstop design, incorporating Phases I-III, tested ceftriaxone in ALS. Stage 1 determined the cerebrospinal fluid pharmacokinetics of ceftriaxone in subjects with ALS. Stage 2 evaluated safety and tolerability for 20-weeks. Analysis of the pharmacokinetics, tolerability, and safety was used to determine the ceftriaxone dosage for Stage 3 efficacy testing.

**Methods:**

In Stage 1, 66 subjects at ten clinical sites were enrolled and randomized equally into three study groups receiving intravenous placebo, ceftriaxone 2 grams daily or ceftriaxone 4 grams daily divided BID. Participants provided serum and cerebrospinal fluid for pharmacokinetic analysis on study day 7. Participants continued their assigned treatment in Stage 2. The Data and Safety Monitoring Board (DSMB) reviewed the data after the last participants completed 20 weeks on study drug.

**Results:**

Stage 1 analysis revealed linear pharmacokinetics, and CSF trough levels for both dosage levels exceeding the pre-specified target trough level of 1 µM (0.55 µg/mL). Tolerability (Stages 1 and 2) results showed that ceftriaxone at dosages up to 4 grams/day was well tolerated at 20 weeks. Biliary adverse events were more common with ceftriaxone but not dose-dependent and improved with ursodeoxycholic (ursodiol) therapy.

**Conclusions:**

The goals of Stages 1 and 2 of the ceftriaxone trial were successfully achieved. Based on the pre-specified decision rules, the DSMB recommended the use of ceftriaxone 4 g/d (divided BID) for Stage 3, which recently closed.

**Trial Registration:**

ClinicalTrials.gov NCT00349622.

## Introduction

Amyotrophic lateral sclerosis (ALS) is a disorder primarily of the motor neurons, which results in progressive wasting and paralysis of voluntary muscles [1]. The incidence of ALS is approximately 2/100,000/year [2]. Average age of onset for sporadic ALS is 55–65 years, though there is a wide variability around this mean, and mean survival is 3–5 years [3]. Studies demonstrate a modest survival benefit of riluzole [4], [5], but the urgent need for novel treatments is clear.

ALS pathophysiology is not fully understood, but glutamate excitotoxicity may be a factor in disease progression. A large body of research supports the role of excitotoxicity in the pathogenesis of ALS (reviewed in [6]). Pointedly, transgenic murine ALS models and human post-mortem tissue have shown decreased excitatory amino acid transporter 2 (EAAT2; mouse analog GLT1), which clears synaptic glutamate [7], [8], [9].

Numerous preclinical studies highlight ceftriaxone as a potential anti-excitotoxic therapy for patients with ALS. The Neurodegeneration Drug Screening Consortium (NDSC) screened 1040 compounds in 29 laboratories using 29 different assays, seven of which were relevant to putative disease mechanisms in ALS. Beta-lactam antibiotics were active in the majority of ALS-related assays, particularly in models related to glutamate excitotoxicity via EAAT2 and mutant SOD1 (mSOD1) toxicity [10], [11].

Ceftriaxone had previously been reported to be neuroprotective in many *in vitro* [12] and *in vivo* models by reducing glutamate excitoxicity [13], [14], [15], [16], [17], though some *in vitro* models did not demonstrate neuroprotection [18]. It increases EAAT2 promoter activation and EAAT2 activity in rodent brains [11], protects motor neurons in culture from excitotoxicity [10], [12], and has antioxidant activity [19], [20]. Finally, ceftriaxone administered to SOD1^G93A^ mice at disease onset slowed the disease course, preserved strength, delayed weight loss, and prolonged survival [11]. In animal models, ceftriaxone increased GLT1 (mouse analog of EAAT2) expression. It increased EAAT2 promoter in human astrocyte cultures at concentrations of 1.0 µM, but not 0.1 µM [11].

In addition to the preclinical data supporting its beneficial effects in models of ALS, ceftriaxone has been reported as neuroprotective *in vitro* [12] and *in vivo* in other neurologic diseases, including spinal muscular atrophy [13], Huntington’s Disease [14] and ischemia [15], [16], [18], and multiple sclerosis [17].

Ceftriaxone is an FDA-approved beta-lactam antibiotic with good central nervous system (CNS) penetration. The usual adult daily dose of intravenous (IV) ceftriaxone is 1–2 grams daily; maximum dosage is 4 grams daily. The typical duration is less than four weeks, though it is occasionally used for up to 8 weeks to treat osteomyelitis or endocarditis. The CSF half-life of ceftriaxone has been estimated at 16.8 hours in studies of patients with external ventriculostomies and half-life in blood is 8.5 hours [21], [22]. Studies of longer treatment with ceftriaxone are limited [23], [24], [25], [26], [27]. The actual CNS/CSF concentration necessary to be neuroprotective in human brain or to induce human EAAT2 in vivo are not known.

Given the need for new therapies for the treatment of ALS, and the large body of research supporting a neuroprotective role for ceftriaxone, the decision was made to test the safety and efficacy of ceftriaxone in patients with ALS. A three-stage nonstop drug development program with intermediate analyses and no pauses between stages was designed to obtain pharmacokinetic (PK) (Stage 1) and safety and tolerability (Stage 2) data, and apply this information to the development of an efficacy study (Stage 3). This design was chosen as a method to expedite drug development (Unpublished Data: Presented by Helms, Kesler, Monti, and Wallace at *FDA -ASA BioPharm Section Workshop*, 2002). We report here on results of the first two stages.

## Methods

### Study Design and Aims

Stage 1 determined the pharmacokinetics (PK) of ceftriaxone in the plasma and cerebrospinal fluid (CSF) of subjects with ALS. Stage 2 determined safety and tolerability over 20 weeks. Stage 3 tested the efficacy of ceftriaxone to slow disease progression.

### Ethics Statement

The study was conducted under an IND (#68,892), approved by the Massachusetts General Hospital (MGH) Coordination Center Institutional Review Board (IRB) and all participating center IRBs, including Harvard Partners, State University of New York Upstate Medical University, Washington University in St. Louis, Wake Forest University, California Pacific Medical Center, Methodist Neurological Institute, Carolinas Medical Center, Indiana University, Emory University, and University of Chicago. The study was listed on clinicaltrials.gov (NCT00349622). The protocol for this trial and supporting CONSORT checklist are available as supporting information; see Checklist S1 and Protocol S1.

An independent Data Safety Monitoring Board (DSMB) reviewed safety and made recommendations regarding the final dosage for Stage 3. All coordination center staff, participants, investigators, site coordinators and site evaluators were blinded to treatment group assignment throughout the study. Screening for Stages 1 and 2 began in August, 2006 and completed in March 2008.

### Stage 1

The aim of Stage 1 was to determine if either dose, or both doses, of ceftriaxone achieve a CSF trough concentration of ≥1 µM in at least 80% of the participants. The PK of ceftriaxone in plasma and CSF were measured to allow estimation of CSF levels based on dosing and plasma level. The PK effect of a drug holiday was also modeled. Participants were randomized equally into treatment arms receiving placebo twice daily, low dose (2 grams per day in the morning and placebo in the afternoon) ceftriaxone (LD) or high dose (4 grams per day divided into 2 grams twice daily) ceftriaxone (HD) administered via indwelling central venous catheter. Placebo was pediatric multivitamin matching the study drug in appearance, taste, and odor.

### Stage 2

The treatment blind was not broken after Stage 1; all subjects continued treatment in their previously assigned dosage group for Stage 2 to determine safety and tolerability after 20 weeks, inclusive of time on study drug in Stage 1. Subjects were regarded as treatment failures if they permanently discontinued study drug prior to completing 20 weeks of treatment for any reason. A dosage was considered tolerable if the proportion of treatment failures was less than 40% with 80% confidence. Safety was also assessed, as defined by the occurrence of serious adverse events (SAEs). Because the study design was non-stop continuous, all participants remained on study drug at the end of Stage 2.

By design, the DSMB performed an interim analysis and made a recommendation about dosage for Stage 3. Stage 2 participants continued to Stage 3 in a blinded fashion. Those on placebo continued on placebo, those on active treatment had their doses adjusted to match the selected Stage 3 dosage. New participants in Stage 3 were randomized to active treatment or placebo in a 2∶1 ratio.

### Participant Selection Criteria

At screening, eligible participants had a diagnosis of probable lab-supported, probable, or definite ALS by El Escorial Criteria [28], a vital capacity (VC) ≥60% of the predicted normal value for height, age and gender, symptom duration of less than 3 years, and were not on riluzole or were on a stable dose for ≥30 days. Trial participants were discouraged from starting or stopping riluzole during the trial but were not prevented from doing so. Exclusion criteria included use of mechanical ventilation, known sensitivity to beta-lactam antibiotics, pregnancy, exposure to investigational agents within 30 days of screening, active gastrointestinal or biliary disease within 30 days of screening, history of antibiotic-induced colitis, or clinically significant abnormal safety laboratory values.

### Study Procedures

Participants signed an informed consent form prior to screening. Medical history, physical and neurological examinations, medication review, VC testing, administration of the revised ALS functional rating scale (ALSFRS-R), vital signs, electrocardiogram (ECG), and laboratory tests were performed. Investigators determined the competence of the participant’s caregiver(s) to administer the study drug at home. Screening procedures took place within 28 days of the baseline (randomization) visit.

Following screening and before placement of the catheter, participants and their caregiver(s) were taught catheter care, signs of line infection, and aseptic infusion technique. Participants and caregivers demonstrated their proficiency at these techniques and passed a catheter-care competency assessment.

### Central Venous Catheter Placement

The single-lumen tunneled central venous (CV) catheter was placed by trained personnel prior to randomization.

### Randomization

The randomization scheme developed by the Biostatistics Center at MGH and assigned each participant to one of the three treatment arms (placebo, LD or HD ceftriaxone) in equal proportion. There was an additional randomization to the time of the lumbar puncture for CSF sampling (2, 4, 6, 8, or 10 hours after dosing). Subjects were not randomized if they had fever or catheter complications, or if caregiver was unable to demonstrate catheter care competency.

### Pharmacokinetic Procedures

Seven days after initiating study drug, plasma drug concentration was measured immediately before and after administration, and every 2 hours thereafter for 12 hours. Blood samples for pre-dose plasma trough levels were taken for every participant at weeks 2, 4, 8, and 12. Samples were centrifuged and the plasma separated and frozen until analysis. Drug concentration in the CSF was measured immediately preceding administration (hour 0) and at a single time point following study drug administration (2, 4, 6, 8, or 10 hours post-drug administration). Plasma and CSF drug concentration analysis was performed by a technician blinded to dosage level, but not time of collection, using high-performance liquid chromatography (HPLC) with ultraviolet detection.

#### Determination of ceftriaxone in plasma and CSF

For determination of ceftriaxone in plasma, calibration tubes were prepared containing varying known concentrations of ceftriaxone (0, and 5 to 40 ug/mL). Drug-free control human or bovine plasma (0.1 uL) was added to calibration tubes, and 0.1 uL of study sample plasma added to other tubes. To each tube was added 0.1 uL of ammonium acetate buffer (0.1 M, pH 5) containing cefazolin as internal standard. After addition of 0.5 mL acetonitrile, samples were vortex-mixed and centrifuged. The supernatant was transferred to a conical tube containing 0.5 mL of chloroform. After vortex mixing and centrifugation, the upper aqueous layer was transferred to an auto-sampling vial for HPLC analysis. The HPLC mobile phase was water:methanol:triethylamine (750∶250∶4), at a flow rate of 1.0 mL/min. The column was Waters micro-Bondapak C18, 3.9 mm × 30 cm. Detection was U. V. absorbance at 270 nm. Calibration curves were linear and passed through the origin. The sensitivity limit was 1.0 ug/mL. Within- and between-day variability did not exceed 15%.

For determination of ceftriaxone in CSF, the method was modified as follows. To the calibration standards (containing 0, and 0.25–20 ug/mL) were added 0.2 mL distilled water; sample tubes contained 0.2 mL of study CSF.

### Safety Procedures

Vital signs, weight, vital capacity and ALSFRS-R were assessed monthly. Safety blood tests were performed weekly for the first 20 weeks of the study. Ceftriaxone is known to cause biliary sludging, and site investigators were instructed to treat study participants with ursodeoxycholic acid (ursodiol) 300 mg twice daily, as indicated for presence of biliary stones or sludge.

### Protocol Deviations

There were 234 protocol exceptions granted: 11 for inclusion/exclusion criteria (4 placebo, 3 LD, 4 HD), nine for procedures performed out of order, 23 for a missed procedure, 17 for methodologic errors regarding sample or study drug processing, 114 for out of window study visits, 11 for patients receiving prophylactic antibiotic for line placement, 23 for additional labs or imaging not specified in the protocol, and 26 to correct a blood draw omitted from the protocol inadvertently. There were 105 protocol violations: three violations for improperly obtained informed consent, 21 for out of window visits, six for out of sequence procedures, seven for additional study procedures, 48 for omitting a test or procedure, six for concomitant use of antibiotics, eight for methodologic errors regarding outcome measure, sample or study drug processing, one for a blood draw omitted from the protocol inadvertently, two for subjects who took an extra dose of study drug in a day, one for failure to report an SAE appropriately, one for a patient who initiated co-enzyme Q, and one for a patient who had a Hickman Catheter placed but was not entered into the study because the patient did not meet inclusion criteria.

### Statistical Analysis

Distributions of baseline characteristics, demographics and screening laboratory tests were compared among the three groups using a Kruskal-Wallis (K-W) test for continuous variables and a Fisher’s exact test for discrete variables.

#### Plasma concentrations

Plasma kinetics of ceftriaxone were consistent with a two-compartment model. Accordingly, plasma concentrations of ceftriaxone during and after the zero-order infusion were analyzed by derivative-free weighted nonlinear least-squares regression analysis (SAS PROC NLIN) to calculate total volume of distribution using the area method (Vd), apparent half-life of distribution and elimination, and total clearance [29]. It was assumed that subjects were at steady-state at the time of the study. Means were compared using Student’s t-test.

#### CSF concentrations

CSF ceftriaxone concentrations were analyzed by nonlinear regression, assuming first-order entry into and removal from CSF. All data points at each dosage level were assumed to be independent, and analyzed simultaneously. Correlation between pre-dose plasma and CSF concentrations were calculated using simple linear regression.

#### Safety and Tolerability (Stages 1 and 2)

Adverse event (AE) frequency was categorized using the Common Terminology Criteria for Adverse Events (CTCAE) Version 4 and analyzed across treatment groups. Biliary AEs and ursodiol use were anticipated and specifically analyzed. Changes in laboratory values relative to baseline were measured weekly for the first 20 weeks of the study and analyzed by repeated-measures ANOVA.

Log-Rank was used to compare median values for time-to-event analyses, and Jonckheere-Terpstra (JT) test or Fisher’s Exact test was used for frequency analyses by treatment group. In accordance with the intention-to-treat principle, all randomized participants were included in the primary statistical analyses according to the treatment group to which they were originally assigned.

The end of Stage 2 was defined as the time at which the final participant completed 20 weeks on study drug. For the primary tolerability analysis only the first 20 weeks of study drug therapy was analyzed for each participant. For secondary analyses, all available data for Stages 1 and 2 was analyzed.

The rates of change in VC, ALSFRS-R, and hand-held dynamometry (HHD) in Stages 1 and 2 of the study remains blinded through completion of Stage 3. SAS version 9.2 was used for statistical analyses.

#### Decision rules for stage 3 dosage level

Dosage level for Stage 3 was determined based on PK and safety/tolerability. If both dosage levels were tolerable, achieved ≥1 µM CSF concentrations, the higher dose was safe (not significantly more serious AEs, and no differences between rates of important AEs (one-sided, p = 0.10 level), then the higher dosage would be chosen for Stage 3. If only the higher dosage level achieved the CSF concentration target (≥1 µM in CSF) and it met tolerability goals, then it would be chosen for Stage 3. If the higher dosage level were not tolerable or safe, and the lower dosage level met tolerability and PK goals, then the lower dosage would be chosen for Stage 3. If neither dose met PK and/or tolerability goals, then the trial would end.

#### Power analysis

With 20 subjects per treatment arm, with one-sided alpha of 0.10, a conclusion that tolerability was >–40% could be made with at least 80% confidence if ≤5 subjects failed to complete the 20-week study. We had ≥80% power to observe at least one occurrence of an AE occurring with a frequency of ≥8% in one treatment arm.

## Results

**Figure 1:**
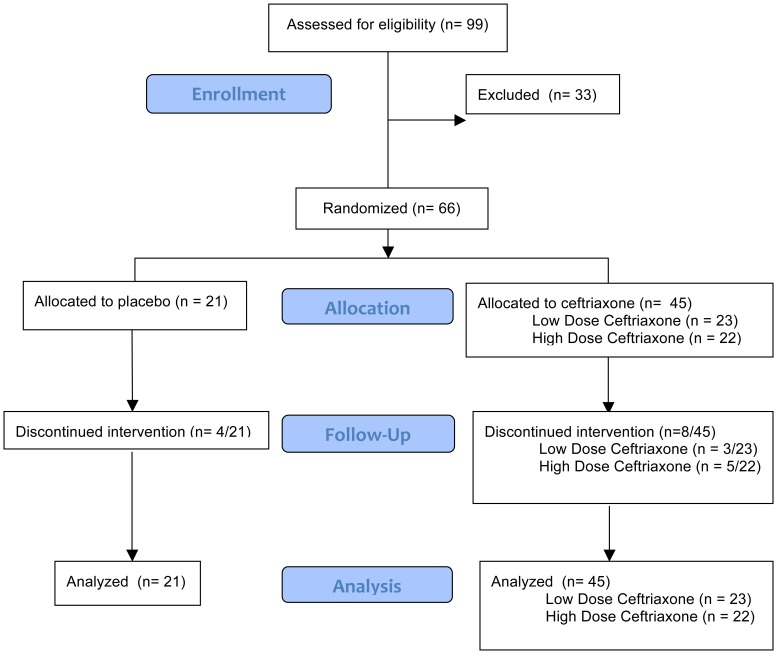
Enrollment, Randomization, Follow-Up and Analysis.

Sixty-six subjects were enrolled in Stages 1 and 2 (Figure 1). Treatment arms were similar on baseline characteristics (Table 1). The baseline ALS Quality of Life Score was highest in the low-dose ceftriaxone group and lowest in the high dose ceftriaxone group (K-W; p = 0.044).

**Table 1:** Demographics.

	Placebo*(mean/SD)*	Ceftriaxone 2 g/day*(mean/SD)*	Ceftriaxone 4 g/day*(mean/SD)*	p-value
Age	51 (11)	49 (10)	54 (13)	0.55
Yrs from Symptom Onset to Diagnosis	0.77 (0.47)	0.99 (0.65)	0.78 (0.43)	0.46
Yrs from Symptom Onset to Screening	1.48 (0.77)	1.60 (0.67)	1.44 (0.60)	0.63
% Male	52%	70%	82%	0.13
% Limb Onset	86%	78%	86%	0.77
% Taking Riluzole	67%	78%	64%	0.56
% Predicted VC	87.4% (15.5%)	94.7% (16.8%)	89.9% (17.1%)	0.24
HHD	−1.6 (0.8)	−1.3 (0.9)	−1.1 (1.1)	0.26
ALSFRS-R	35.2 (5.7)	37.5 (5.7)	36.8 (6.0)	0.47
ALS QOL	429 (70)	457 (65)	405 (64)	0.04
V_d_ in Liters	−	13.7	13.9	0.88
V_d_ in Liters/kg	−	0.171	0.171	1.00
T_1/2_ in Hours	−	9.1	8.0	0.12
Clearance in mL/min	−	17.5	20.5	0.04
Clearance in mL/min/kg	−	0.22	0.25	0.23

VC – Vital Capacity; HHD - Hand-Held Dynamometry; ALSFRS-R – Revised ALS Functional Rating Scale; ALSQOL – ALS Quality of Life Questionnaire; V_d_ – Volume of Distribution; T_1/2_– Half-life; mL – milliliters; min – minutes; kg - kilograms.

### Pharmacokinetic Analysis

**Figure 2:**
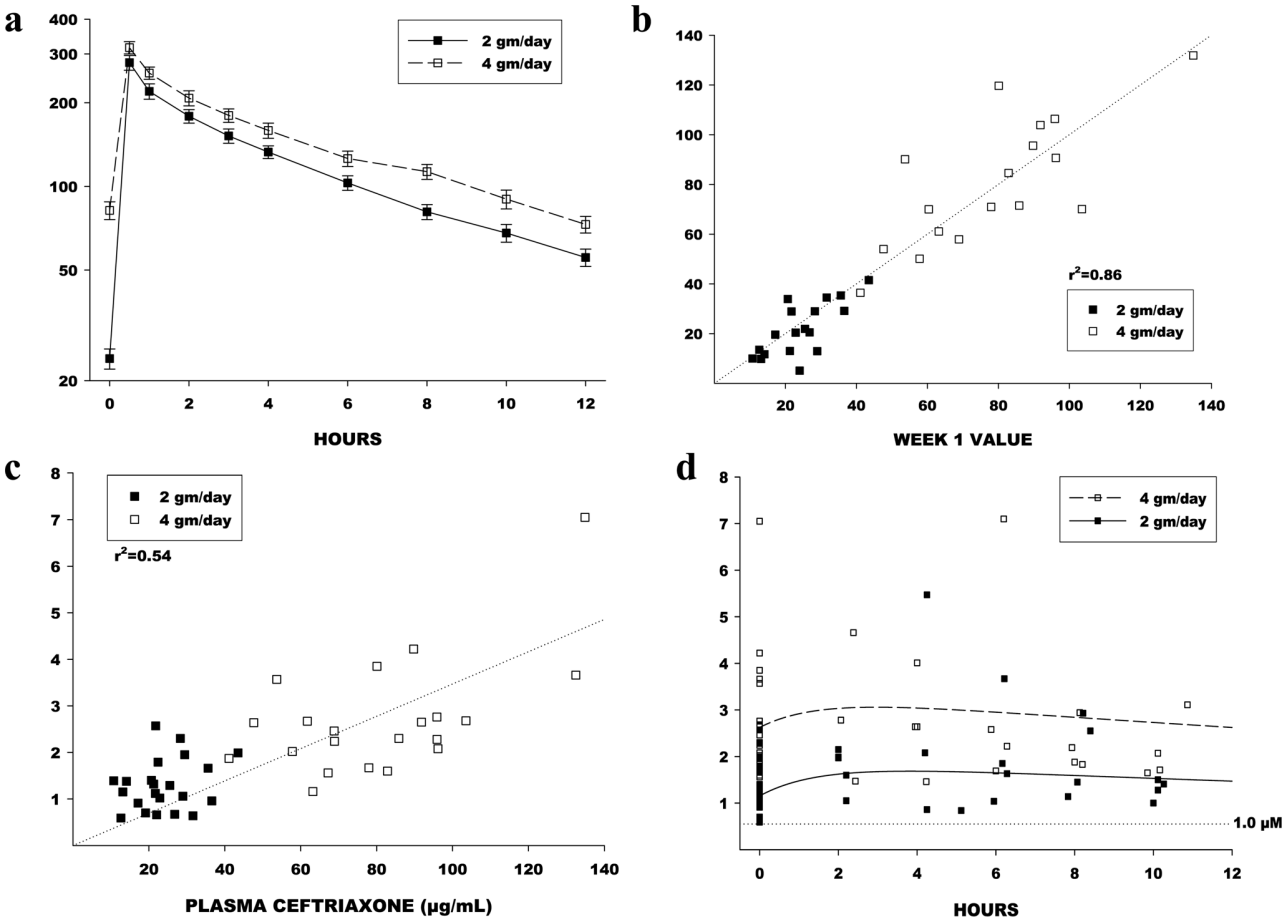
Plasma and CSF Pharmacokinetics. a. Mean and SE plasma ceftriaxone concentrations at corresponding times for both LD and HD arms. Levels are always higher with HD, relative to LD. b. Pre-dose (trough) plasma levels at Week 1 were highly correlated with the pre-dose levels at Week 4, indicating that steady-state had been reached by Week 1. Dashed line is the line of identity (y = x). c. Pre-dose CSF and plasma concentrations were significantly correlated using simple linear regression. The r-square value (0.54) indicates that plasma levels explain about 54% of variability in CSF levels. The slope of the regression line is about 0.04, indicating that CSF levels are much lower than plasma levels. Assuming that CSF uptake happens by passive diffusion, the difference is probably explained by plasma protein binding of ceftriaxone. d. Actual CSF concentrations, and the predicted “typical” concentration curves for the 2 gm/day and 4 gm/day dosage groups. Also shown is the boundary of 1.0 micromolar, equivalent to 0.55 µg/mL. This is the minimum effective concentration based on *in vitro* studies and the goal concentration for Stage 1.

**Figure 3:**
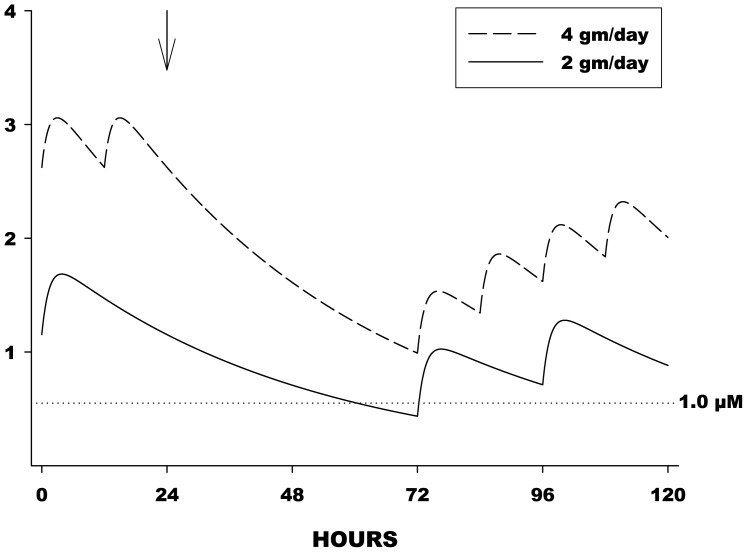
Effect of Drug Holiday on CSF Drug Levels. Predictive illustration of the effect of “drug holidays” at steady-state. Drug holiday began at the arrow (24 hours). Dosing then resumed as usual at 72 hours. Despite the holiday, CSF levels remained above 1 micromolar in the 4 gm/day group, and fell slightly below 1 micromolar in the 2 gm/day group.

Ceftriaxone had a mean volume of distribution of 13.8 liters and a plasma half-life of 8.6 hours. Mean plasma concentration levels for LD and HD arms are presented in Figure 2a. Trough drug levels in plasma were highly correlated (r^2^ = 0.86) at 1 and 4 weeks suggesting steady-state drug levels at day 7 (Figure 2b). Plasma and CSF concentrations were correlated (r^2^ = 0.54) (Figure 2c). CSF trough levels for both dosage levels exceeded the prespecified target trough level of 1 µM (0.55 µg/mL) (Figure 2d). In the HD arm, modeling predicted that CSF levels would stay above 1 µM for 72 hours off study drug, enabling drug holidays of up to 72 hours if needed, while in the LD arm, levels dipped below 1 µM between 48 and 72 hours off drug, necessitating drug holidays be limited to 48 hours (Figure 3). There was no effect of riluzole on plasma PK of ceftriaxone.

### Tolerability

**Figure 4:**
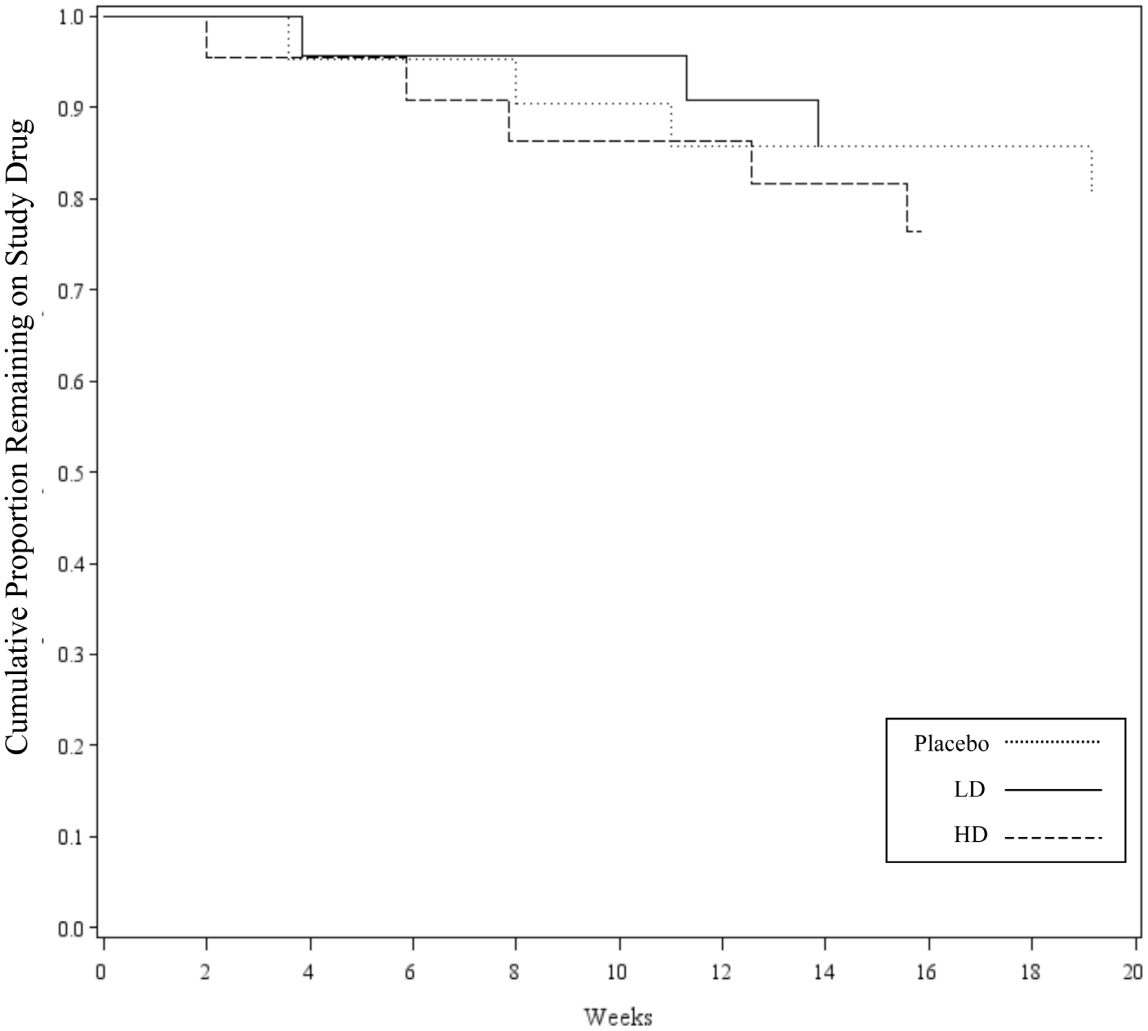
Time to Discontinuation of Study Drug by Treatment Arm (until 20 weeks). Kaplan-Meyer curve showing time to discontinuation of study drug within the first 20 weeks for patients who discontinued study drug within this time frame. Time to discontinuation of study drug was not significantly different between treatment arms (p = 0.90; Log-Rank). LD – Low Dose Ceftriaxone Arm; HD - High Dose Ceftriaxone Arm.

All dosages met the prespecified criterion for tolerability at 20 weeks (Figure 4). There was no dose-dependent effect on time to early study medication discontinuation (p = 0.90; Figure 4). Four participants did not complete the first 20 weeks of treatment in the placebo group, three in the low-dose ceftriaxone group, and five in the high-dose ceftriaxone group. Discontinuation was due to AEs in one subject in the placebo arm, three in the LD arm, and two in the HD arm. One catheter-related adverse event led to discontinuation in the placebo arm. ALS progression accounted for one discontinuation in the placebo arm. Subject choice led to one discontinuation in each treatment arm. Caregiver choice led to one discontinuation in the placebo arm and one in the HD arm. (Table 2; all NS).

**Table 2:** Reasons for Drug Discontinuation in Stages 1 and 2.

	Placebo *(n = 21)*	Ceftriaxone 2 g/day *(n = 23)*	Ceftriaxone 4g/day *(n = 22)*	Pooled Fisher-Exact p-value
Adverse Event	0	3*	2**	0.17
Catheter-Related Adverse Event	1	0	0	0.32
ALS Progression	1	0	0	0.32
Subject Choice	1	1	1	1.00
Caregiver Choice	1	0	1	0.54
*Total*	*4*	*4*	*4*	*1.00*

* Pseudomembranous colitis, Pruritis, Neutropenia.

** Pulmonary edema, Cholelithiasis.

Secondary analysis of dosage reduction and temporary drug suspension was performed using all data collected up to the point that the final participant had completed 20 weeks on study drug (completion of Stage 2). The average duration of follow-up for this analysis was 36 weeks. Drug dosing was temporarily suspended 13 times in 8 patients in the placebo arm, 23 times in 16 patients in the LD arm, and 16 times in 12 patients in the HD arm (p = 0.046). Dosage was reduced in 1 subject in the placebo arm, 8 in the LD arm, and 8 in the HD arm (p = 0.017).

### Safety

**Figure 5:**
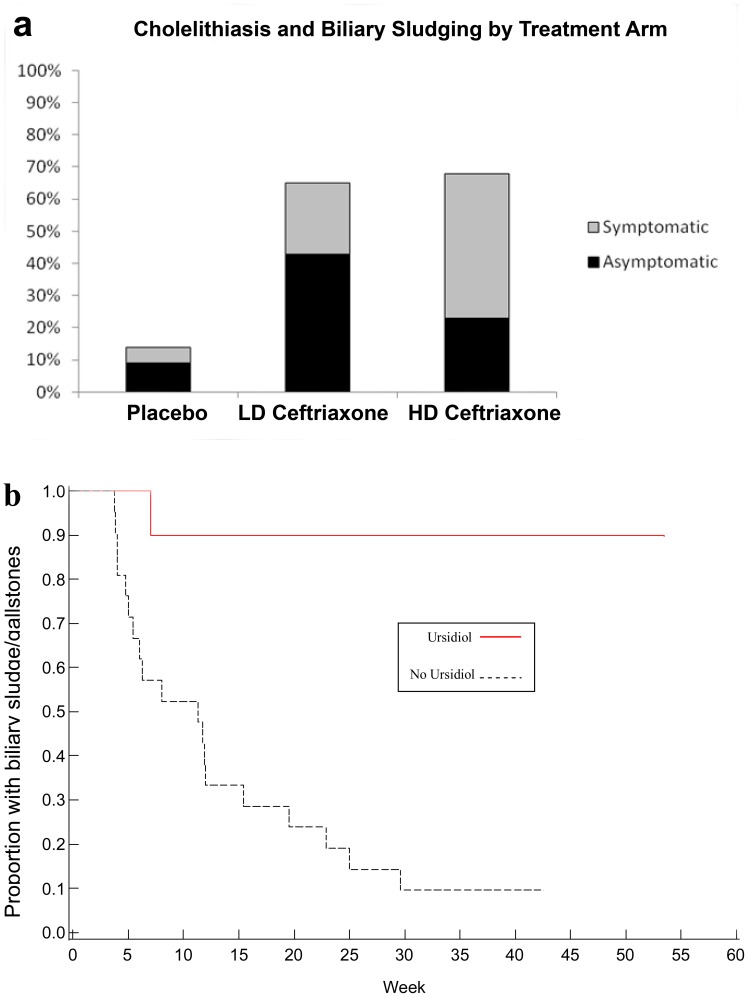
Cholelithiasis and Biliary Sludging by Treatment Arm. (a) Biliary sludging and cholelithiasis were more common in the low dose (LD) and high dose (HD) treatment arms than in the placebo treatment arm (p<0.01; Jonckheere-Terpstra). (b) Treatment with ursodiol significantly shortened event duration when used to treat cholelithiasis and biliary sludging (p,0.001; Log-Rank).

Based on the pre-specified criteria, ceftriaxone was deemed acceptably safe at all dosages tested. Occurrence of abdominal pain and cholelithiasis were more common in ceftriaxone-treated subjects (p = 0.02 and p<0.0001, respectively; Figure 5a). Ursodiol significantly shortened duration of the event (p<0.001; Figure 5b) and was used by four subjects in the placebo arm (19%), 13 in the LD arm (57%), and 13 in the HD arm (59%) (p<0.01). Limb edema was more common in the placebo arm (p = 0.05). No other AEs differed significantly between arms. Forty-four SAEs were reported (Table 3). Percutaneous endoscopic gastrostomy (PEG) tube placement was more common in subjects in the placebo arm (p<0.02; Fischer’s Exact). No other SAEs were statistically significantly more common in any of the treatment groups. There was one death in the HD arm and one in the placebo arm, one of which occurred after catheter placement but before the subject received study drug, and was unrelated to the catheter.

**Table 3:** Serious Adverse Events.

Adverse Event	All*(% (n/total))*	Placebo *(% (n/total))*	LD *(% (n/total))*	HD *(% (n/total))*	JT p-value	Pooled Rx Fisher's p-value
Dyspnea (shortness of breath)	15 (10/66)	19 (4/21)	17 (4/23)	9 (2/22)	0.3665	0.7139
PEG Placement (outpatient)	14 (9/66)	29 (6/21)	9 (2/23)	5 (1/22)	**0.0256**	**0.0242**
Cholelithiasis	11 (7/66)	0 (0/21)	13 (3/23)	18 (4/22)	0.0827	0.0874
Line infection	8 (5/66)	10 (2/21)	13 (3/23)	0 (0/22)	0.1942	0.6499
Thrombosis/thrombus/embolism	3 (2/66)	5 (1/21)	0 (0/23)	5 (1/22)	1.0000	0.5385
Pnuemonia	4 (2/66)	0 (0/21)	8 (2/23)	0 (0/22)	0.7846	1.0000
CNS cerebrovascular ischemia	2 (1/66)	0 (0/21)	4 (1/23)	0 (0/22)	1.0000	1.0000
Clostridium difficile colitis	2 (1/66)	0 (0/21)	4 (1/23)	0 (0/22)	1.0000	1.0000
Constipation	2 (1/66)	5 (1/21)	0 (0/23)	0 (0/22)	0.3182	0.3182
Sudden Death	2 (1/66)	0 (0/21)	0 (0/23)	5 (1/22)	0.6515	1.0000
Fever (in the absence of neutropenia)	2 (1/66)	5 (1/21)	0 (0/23)	0 (0/22)	0.3182	0.3182
Subarachnoid hemorrhage	2 (1/66)	0 (0/21)	4 (1/23)	0 (0/22)	1.0000	1.0000
Abdomen Pain NOS	2 (1/66)	5 (1/21)	0 (0/23)	0 (0/22)	0.3182	0.3182
Pancreatitis	2 (1/66)	0 (0/21)	0 (0/23)	5 (1/22)	0.6515	1.0000
Pulmonary edema:	2 (1/66)	0 (0/21)	0 (0/23)	5 (1/22)	0.6515	1.0000
Hysterectomy:	2 (1/66)	5 (1/21)	0 (0/23)	0 (0/22)	0.3182	0.3182
Thrombosis/embolism (vascular access-related)	2 (1/66)	5 (1/21)	0 (0/23)	0 (0/22)	0.3182	0.3182

Small changes occurred in several of the safety laboratory tests in Stage 1 (<10%). The only statistically significant changes were increased bilirubin with treatment at weeks 2 (p<0.01), 12 (p<0.01), and 16 (p<0.05).

Central venous catheter was well tolerated. Six SAEs were definitely or probably related to the central catheter, including four line infections, one fever, and one thrombotic event – all resolved. Probably or definitely related AEs included skin reaction to tape (2), suture-related problems (2), redness or drainage at the exit site (5), line displacement/dysfunction (6), pain during insertion or removal (2), chills/vomiting (2), and non-SAE infections (2).

## Discussion

Stages 1 and 2 of our three-stage continuous study design led to the efficacy trial without necessitating a pause between study phases. Stages 1 and 2 provided information about the long-term use of ceftriaxone, CSF PK in patients without inflamed meninges, and the dosage required to reach CSF levels of *in vitro* efficacy (1 µM) [10], [11], [12].

Stage 1 met its primary aim, demonstrating that both dosage levels of ceftriaxone (2 grams and 4 grams daily) achieved CSF concentrations of ≥1 µM (0.55 µg/mL). Drug holidays of up to 3 days were possible while maintaining effective CSF drug concentrations ≥1 µM in patients receiving high dose (4 gm/d) ceftriaxone. We confirmed ceftriaxone plasma and CSF kinetics and determined that approximately 50–55% of the variability of between-subject CSF concentration of ceftriaxone was attributable to plasma concentration.

Both dosage levels of ceftriaxone met criteria for tolerability in Stages 1 and 2. As anticipated, the most common drug related AE was biliary sludge/cholelithiasis, which were well-managed with ursodiol and/or temporary drug suspension or reduction, and did not lead to study drug discontinuation in a dose-dependent manner. In Stage 3, all participants receiving ceftriaxone also received ursodiol for the prevention of biliary obstruction.

Other AEs did not occur more commonly with ceftriaxone treatment relative to placebo. The safety of home administration of study drug by trained caregivers was excellent and compared favorably to other studies employing at home catheter access (data not shown).

Based on the pre-established guidelines, the DSMB reviewed pharmacokinetic, and safety and tolerability data and recommended continuing to Stage 3 with the high dosage ceftriaxone (4 g/day divided twice daily). A steering committee subpanel also reviewed the data and came to the same conclusion. Stage 3 data collection is now complete and efficacy data is being analyzed and will be reported in a separate manuscript.

## Supporting Information

Checklist S1 **CONSORT Checklist.**
(DOC)

Protocol S1 **Trial Protocol - Clinical Trial of Ceftriaxone in Subjects with Amyotrophic Lateral Sclerosis.**
(PDF)

File S1 **Author Appendix - Northeast ALS Consortium (NEALS) authors.**
(DOC)
