# Supplementary material for: Design and Initial Results of a Multi-Phase Randomized Trial of Ceftriaxone in Amyotrophic Lateral Sclerosis
Source: PLoS One. 2013 Apr 17;8(4):e61177. doi: 10.1371/journal.pone.0061177 (PMC3629222; doi:10.1371/journal.pone.0061177)
Supplement: Protocol S1 — Trial Protocol - Clinical Trial of Ceftriaxone in Subjects with Amyotrophic Lateral Sclerosis. (PDF) [file pone.0061177.s002.pdf]

Clinical Trial of Ceftriaxone in Subjects with Amyotrophic Lateral Sclerosis

**Coordination Center:**	Neurology Clinical Trials Unit<br>Massachusetts General Hospital<br>13th Street, Building 149, Room 2274<br>Charlestown, MA 02170<br>617 726-9122
**Study Chair:**	**Merit Cudkowicz, MD, MSc**.<br>Associate Professor of Neurology<br>Harvard Medical School<br>Massachusetts General Hospital
**Co-Investigators:**	**Jeremy Shefner, MD, PhD**<br>Professor of Neurology<br>State University of New York, Syracuse
	**Swati Aggarwal, MD**<br>Instructor in Neurology<br>Harvard Medical School<br>Massachusetts General Hospital
**Biostatisticians:**	**David Schoenfeld, PhD**<br>Professor of Medicine<br>Massachusetts General Hospital<br>Harvard Medical School
	**Kerrie Nelson, PhD.**<br>Biostatistician<br>Massachusetts General Hospital<br>Harvard Medical School

**Supported By:**	The National Institute of Neurological Disorders and Stroke (NINDS)<br>**GRANT NUMBER: 1 U01 NS049640**
**Sponsor of IND:**	Merit Cudkowicz, MD<br>**IND NUMBER 68,892**
**Protocol Date:**	**04 December 2008**
**Protocol Version:**	**Version 11**

The information contained herein is confidential and proprietary in nature, and will not be disclosed to any third party without written approval of NEALS or its authorized designee. This document may be disclosed to the appropriate institutional review boards or to duly authorized representatives of the US Food and Drug Administration or a national regulatory authority under the condition that they maintain confidentiality.

TABLE OF CONTENTS

INVESTIGATOR'S AGREEMENT 6

STUDY DESIGN AND OUTCOMES 7

ADMINISTRATION OF INTERVENTION 8

LIST OF ABBREVIATIONS 11

1. STUDY OBJECTIVES 14

1.1 Specific Aims 14

1.2 Study Overview 15

2 BACKGROUND AND SIGNIFICANCE 15

2.1 Background and Rationale 15
2.1.1 Clinical Features and Epidemiology of ALS 15
2.1.2 Overview of ALS Pathogenesis 16
2.1.3 Glutamate Excitotoxicity in ALS 16
2.1.4 NINDS Drug Screening- Identification of Potential ALS Therapeutics 18
2.1.5 Rationale for Choosing Ceftriaxone 19
2.1.6 Experimental Therapeutics in ALS. 21
2.1.7 Significance 22

2.2 Supporting Data 22
2.2.1 Ceftriaxone Protects Against Motor Neuron Degeneration 22
2.2.2 Safety and Clinical Use of Ceftriaxone in Humans 24
2.2.3 Risks of Central Venous Catheters 27
2.2.4 Risks of Pediatric Multivitamin Solution 29
2.2.5 Potential Benefits 30

3. STUDY DESIGN 30

3.1 Study Design Overview (Figure 5) 30

3.2 Study Flow 32

3.3 Setting 32

4. SUBJECT SELECTION AND ENROLLMENT 32

4.1 Inclusion Criteria 32

4.2 Exclusion Criteria 33

4.3 Enrollment Procedures 34
4.3.1 Subject Availability and Recruitment 34
4.3.2 Documentation of Screening and Eligibility 34
4.3.3 Informed Consent 34
4.3.4 Randomization and Randomization Number Assignment 35

5. STUDY INTERVENTIONS 35

5.1 Study Drug – Interventions, Administration, and Duration 35
5.1.1 Dosage 35
5.1.2 Study Drug Administration 36

5.2 Handling of Study Interventions 38
5.2.1 Study Drug Distribution to Sites 38
5.2.2 Study Drug Dispensing, Labeling and Storage 38
5.2.3 Compliance and Return of Study Drug (Adherence Assessment) 39

5.3 Concomitant Medications 39
5.3.1 Exclusionary Medications 39

5.4 Dosage Changes 40
5.4.1 Dosage Reduction 40
5.4.2 Dosage Suspension 40
5.4.3 Dosage Re-challenge 40
5.4.4 Dosage Discontinuation 40

5.5 Code Break Procedures 41

6. CLINICAL AND LABORATORY EVALUATIONS 41

6.1 Schedule of Assessments 41

6.2.1 Timing of Evaluations 46
6.2.1 Screening and Informed Consent 46
6.2.2 On-Study Evaluations 47
6.2.3 Intervention Discontinuation Evaluations 51
6.2.4 Final Evaluations and Post Intervention Phone Call 52

6.3 Special Instructions and Definitions of Evaluations 52
6.3.1 Informed Consent 52
6.3.2 Documentation of ALS 52
6.3.3 Protocol Violations. 52
6.3.4 Clinical Outcome Measures 53

7. MANAGEMENT OF ADVERSE EXPERIENCES 57

7.1 Adverse Experiences Related to Ceftriaxone Use 57

7.2 Adverse Experiences Related to Ursodiol 60

7.3 Adverse Experiences related to Central Venous Catheter 60

8. CRITERIA FOR INTERVENTION SUSPENSION, RECHALLENGE, OR PERMANENT DISCONTINUATION 63

8.1 Intervention Suspension Related to Ceftriaxone Use 63

8.2 Intervention Suspension Due to Catheter Complications 64

9. STATISTICAL CONSIDERATIONS 64

9.1 General Design, Data Analysis and Power 64
9.1.1 STAGE 1 Pharmacokinetic Analysis and Sample Size 65
9.1.2 STAGE 2 Safety and Tolerability Study 66
9.1.3 STAGE 3 Efficacy Trial 67

9.2 Outcomes 72

9.3 Sample Size and Accrual 72

9.4 Data and Safety Monitoring 72
9.4.1 Monitoring and Reporting Process 72
9.4.2 The Medical Monitor 73

9.5 Data Analyses 73

10. DATA COLLECTION, SITE MONITORING, AND ADVERSE EXPERIENCE MONITORING 73

10.1 Records to be Kept and Project Organization 73

10.2 Role of Data Management 74
10.2.1 Data Flow and Management Overview 74
10.2.2 Database Security 76
10.2.3 Data Lock Process 77

10.3 Quality Assurance 77

10.4 Adverse Experiences Monitoring and Reporting 78
10.4.1 Evaluating and Recording of Adverse Events 80

11 HUMAN SUBJECTS 81

11.1 Institutional Review Board (IRB) Review and Informed Consent 81

11.2 Subject Confidentiality 81

11.3 Inclusion of Women 82

11.4 Inclusion of Minorities 82

11.5 Study Modification/Discontinuation 83

12 PUBLICATION OF RESEARCH FINDINGS 83

13 REFERENCES 84

INVESTIGATOR'S AGREEMENT

I have read the attached protocol entitled, "Clinical Trial of Ceftriaxone in Subjects with Amyotrophic Lateral Sclerosis," dated **04 DECEMBER 2008** (**Version 11.0**) and agree to abide by all described protocol procedures. I agree to comply with the World Medical Association Declaration of Helsinki: Ethical Principles for Medical Research Involving Human Subjects, the International Conference on Harmonisation Tripartite Guidelines on Good Clinical Practice, applicable U.S. Food and Drug Administration (FDA) regulations and guidelines identified in 21 CFR Parts 11, 50, 56, and 312.7, the applicable provisions of sections 402(i) and 402(j) of the U.S. Public Health Service Acts (PHS Act) [42 U.S.C. §§ 282 (i) and (j)], amended by Title VII of the FDA Amendments Act of 2007 (Public Law No. 110-85, 121 Stat.904), all Health Canada applicable regulations and guidelines including the Therapeutic Products Directorate's Guideline for Good Clinical Practice, local Institutional Review Board (IRB) guidelines and policies, and the U.S. Health Insurance Portability and Accountability Act (HIPAA).

Site Principal Investigator Signature: ____________________________ Date: __________

Print Site Principal Investigator Name: ____

PRÉCIS

Title

Clinical Trial of Ceftriaxone in Subjects with ALS

Objectives

Primary Objectives

The objectives of this study are to determine the pharmacokinetics and tolerability of long term ceftriaxone treatment and to subsequently determine the efficacy of this treatment in subjects with ALS.

Secondary Objectives

We will measure multiple aspects of decline in subjects with ALS and correlate these outcome measures with the change in survival due to treatment effect.

Background and Rationale

Amyotrophic lateral sclerosis is a uniformly progressive and fatal neurodegenerative disorder for which there is no known cure. The pathogenesis is largely unknown; however, several studies implicate glutamate toxicity, free radical toxicity, toxicity from heavy metals and mitochondrial dysfunction as possible causes of both sporadic and familial ALS. Therapeutic strategies in ALS include those that are targeted at improving glutamate transport and those that protect against cell death from other pathways including apoptosis, metal toxicity and oxidative stress. Cephalosporins are a class of compounds that may have these properties.

Study Design and Outcomes

We propose a double-blind, placebo controlled clinical trial of ceftriaxone in 600 subjects with ALS. The study uses a sequential, non-stop drug development design. This design was chosen as a method to expedite drug development, while safely testing a new therapeutic agent in ALS. We will determine optimal dosage, safety and efficacy of ceftriaxone in ALS. Our goals are to determine the steady state levels and population kinetics of ceftriaxone in the CSF (Aim 1, STAGE 1), define the safety and tolerability of ceftriaxone after a minimum of 20 weeks of daily treatment (Aim 2, STAGE 2) and to ascertain whether chronic treatment with ceftriaxone prolongs survival and slows the rate of decline in function in subjects with ALS (Aim 3, STAGE 3).

In STAGE 1, approximately 60 subjects at up to 12 centers will be enrolled in a pharmacokinetic study. Subjects will receive placebo, 2 or 4 grams ceftriaxone daily. After seven days they will be admitted to the hospital for a plasma and CSF pharmacokinetic study. All STAGE 1 subjects will continue treatment and enter STAGE 2 to determine safety and tolerability after 20 weeks of daily treatment. STAGE 1 results will be used to modify the STAGE 2 design. Based on data from both STAGES 1 and 2, an assessment will be made as to whether a dosage producing sufficient CSF levels to generate a biological effect can be tolerated by subjects with ALS. If the decision is affirmative, the final study dosage will be determined and the study will be expanded

to 600 randomized subjects at approximately 70 centers in US and Canada. Subjects in the STAGE 1 and 2 studies will roll-over into the larger phase, with the two active treatment groups receiving ceftriaxone at the single dosage chosen for further study. Subjects will remain in the study until 52 weeks after the last subject is randomized.

The **co-primary outcome measures** will be the difference in survival and the rate of decline in function as measured by the ALS functional rating scale-revised (ALSFRS-R) between the treatment and placebo groups at the end of study. While change in ALSFRS-R can reliably be determined in a one year study period, detecting changes in survival require a longer duration of measurement. Thus, the survival endpoint accounts for the variable study duration for individual subjects, while employing all available data for each subject. Subjects will remain in the study until 52 weeks after the last subject is randomized, allowing time for events to occur with respect to the survival endpoint. Survival is defined as time to death, tracheostomy or the initiation of permanent assisted ventilation (PAV).

Secondary outcome measures will include vital capacity, evaluation of multiple upper and lower extremity muscles using hand held dynamometry, quality of life measurements for subjects and caregivers, the long-term safety and tolerability of ceftriaxone in this population, the ability to complete the study on assigned dosage and frequency of adverse events.

The use of an electronic data capture system will allow for rapid decision making with regard to study continutation and final dosage selection. For all stages of the study, adverse event monitoring will be performed on a regular basis, in addition to the outcome measures.

Included in this study is one sub-study: Caregiver Burden Inventory (caregiver quality of life measure). Details of this substudy are found in Appendix 4 of the protocol and the informed consent form is found in Appendix 3. Site investigators (or an IRB approved designee at the site) will obtain consent from Caregivers for the Caregiver Burden Inventory.

Interventions and Duration

Administration of Intervention

Ceftriaxone and matching placebo will be supplied frozen to the subjects in pre-filled syringes. Study drug is dispensed at baseline, weeks 4, 8, 12, 16 and every four weeks for the duration of the study.

STAGE 1 and 2 study: The placebo is pediatric multivitamin infusion (MVI) in sterile normal saline. The placebo concentration will be 0.20 ml +/- 0.05 ml of MVI in 20 cc of normal saline. This range exists in order to adjust the color of the placebo, particularly from different lot numbers, for blinding. The change in concentration will not affect the safety of the placebo. Ceftriaxone will be supplied as 2 grams dissolved in 20 ccs of sterile water. Each subject will administer the treatment twice a day, seven days per week. Each dose is 20 ccs. In STAGE 1, the subjects will receive two containers of syringes labeled Container A (or "a.m.") and Container B (or "p.m.") filled with study drug. Caregivers will be instructed to administer to the subject the contents of one syringe from Container A in the morning and one from Container B in the evening as follows:

	Container	
**Group**	**A**	**B**
2 grams per day	X	0
4 grams per day	X	X
Placebo	0	0

("0" = Placebo, "X" = ceftriaxone 2 grams)

	AM	PM
Day 0 – STAGE 2 conclusion	A	B

STAGE 3 study: The final dosage for Stage 3, based upon data obtained in Stage 1 and 2, will be 4 g (2 g bid). Study drug will be administered as follows:

	Time	
**Group**	**AM**	**PM**
4 grams per day	X	X
Placebo	0	0

("0" = Placebo, "X" = ceftriaxone 2 grams)

Duration of Participation

The total study length from first enrolled subject is approximately 59 months: 18 months enrollment for STAGE 1, 20 weeks of follow-up in STAGE 2, 24 months for subject recruitment for STAGE 3 and 52 weeks of additional follow-up. It will take approximately seven months for intermediate analyses.

Subject Recruitment, Sample Size and Population

Participants in this study will be subjects with familial or sporadic ALS. Diagnostic and Inclusionary/Exclusionary criteria will be clearly outlined in the protocol. Approximately 60 subjects will participate in the study at up to 12 sites for STAGE 1 and STAGE 2, and these subjects will continue on to STAGE 3 of the study. STAGE 3 will include an additional 540 subjects, for a total of 600 subjects at approximately 70 sites in the US and Canada.

Randomization

Approximately 60 eligible subjects from up to 12 clinical centers will be randomized (1:1:1) in STAGE 1 to ceftriaxone 2 grams per day, 4 grams per day, or color-matched placebo. All STAGE 1 subjects will participate in a pharmacokinetic study seven days after starting study treatment and then proceed into STAGE 2 in the same treatment arm assignment. Once data from STAGE 2 is analyzed and a decision is made on final dosage and treatment schedule for the STAGE 3 efficacy trial, the STAGE 1subjects assigned to the placebo arm will continue on placebo; and the STAGE 1 subjects assigned to ceftriaxone (2 grams or 4 grams/day) will be

reassigned to the ultimate ceftriaxone dosage chosen for the efficacy trial (STAGE 3). The additional 540 subjects entering STAGE 3 will be randomized (2:1) to ceftriaxone or matched placebo. The randomization will be stratified by clinical site and riluzole use. Randomization will be blocked to ensure that the treatment groups are balanced within a site after a certain number of subjects have been enrolled at that site.

LIST OF ABBREVIATIONS

AAN	American Academy of Neurology
AE	Adverse Event/Experience
AIN	Acute allergic interstitial nephritis
ALS	Amyotrophic Lateral Sclerosis
ALSFRS-R	Amyotrophic Lateral Sclerosis Functional Rating Scores – revised
ALSSQOL	ALS-Specific Quality of Life Measure
ALT	Alanine aminotransferase/serum glutamic oxaloacetic transaminase/SGOT
AMPA	Amino-3-hydroxy-5-methyl-4-isoxazolepropionate
ANC	Absolute neutrophil count
AST	Aspartate aminotransferase
ATN	Acute tubular necrosis
bid	Bis in diem/twice a day
BUN	Blood Urea Nitrogen
C	Plasma ceftriaxone concentration
CAP	College of American Pathologists
CBC	Complete Blood Count
CBI	Caregiver Burden Inventory
CDC	Centers for Disease Control
CE	Clinical evaluator
CRF	Case report form
CFR	Code of Federal Regulations
CLIA	Clinical Laboratory Improvement Amendments
CNS	Central nervous system
CNTF	Ciliary neurotrophic factor
COSTART	Coding Symbol Thesaurus for Adverse Event Reporting
CSF	Cerebrospinal fluid
CTA	Clinical Trial Application
CTAB	Cetyltrimethylammonium bromide
CTCAE	Common Terminology Criteria for Coding Adverse Events
CV	Curriculum vitae
CVC	Central Venous Catheter
DM	Data Management
DSMB	Data Safety Monitoring Board
DVT	Deep vein thrombosis
EAA	Excitatory amino acid
EAAC1	Excitatory amino acid carrier 1
EAAT	Excitatory amino acid transporter
EC_{50}	Effective concentration, 50%
eCRF	Electronic Case Report Form
EDC	Electronic data capture
ELISA	Enzyme-linked immunosorbent assay
FALS	Familial amyotrophic lateral sclerosis
FDA	Food and Drug Administration

FVC	Forced vital capacity
GCP	Good Clinical Practice
GCRC	General Clinical Research Center
GLAST	Glutamate aspartate transporter
GLT-1	Glutamate transporter –1
HC	Canadian Therapeutics Products Division of Health Canada
HD	Huntington's Disease
HHD	Hand held dynamometry
HPLC	High performance liquid chromatography
HRC	Human Resource Committee
ICH	International Conference on Harmonisation of Technical Requirements for Registration of Pharmaceuticals for Human Use
IGF-1	Insulin-like growth factor
IND	Investigational New Drug
IRB	Institutional Review Board
IV	Intravenous
K_a	First-order entry rate constant
K_e	Exit rate constant
LFT	Liver function test
LMW	Low molecular weight
MGH	Massachusetts General Hospital
MMT	Manual muscle testing
MND	Motor neuron disease
MOP	Manual of Operations
MRC	Medical Research Council
MRSA	Methicillin resistant staph aureus
mRNA	Mono ribonucleic acid
MVI	Multi-Vitamin Infusion
MVIC	Maximum voluntary isometric contraction
MVMC-HHD	Maximum voluntary muscle contraction using hand-held dynamometry
N2A	Neuro 2A
NCI	National Cancer Institute
NCTU	Neurology Clinical Trials Unit
NDSC	Neurodegeneration Drug Screening Consortium
NEALS	Northeast Amyotrophic Lateral Sclerosis
NIH	National Institutes of Health
NINDS	National Institute of Neurological Disorders and Stroke
NMDA	N-methyl-D-aspartate
NP	Nurse Practitioner
ODBC	Open Database Connectivity
OHRP	Office for Human Research Protections
PA	Physician's Assistant
PAV	Permanent assisted ventilation
PDF	Portable Document Format
PI	Principal Investigator

PRP	Platelet-rich plasma
PT	Prothrombin time
q	Quaque / once-a-day
Q	Zero-order ceftriaxone infusion rate
RN	Registered Nurse
RS2	Randomization system 2
SAE	Serious adverse event
SALS	Sporadic amyotrophic lateral sclerosis
SDF	Source Document Form
SC	Subcutaneous
SOD1	Superoxide dismutase-1
SSL	Secure sockets layer
SUNY	State University of New York
SWFI	Sterile water for injection
T	Telephone data
t	Time after the start of infusion
TGF	Transforming growth factor
UV	Ultraviolet
V_1	Apparent volume of the central compartment
VC	Vital capacity
Vd or V_d	Volume of distribution
WBC	White blood cells
WHO	World Health Organization

1. STUDY OBJECTIVES

1.1 Specific Aims

The **study objective** is to determine the safety and efficacy of ceftriaxone, a Food and Drug Administration (FDA) approved semi-synthetic third-generation cephalosporin, in subjects with amyotrophic lateral sclerosis (ALS). The **study hypothesis** is that intravenous (IV) ceftriaxone will slow disease course in patients with ALS. In a National Institute of Neurological Disorders and Stroke (NINDS)-led cooperative group *in-vitro* screening program of 1040 FDA approved drugs in 29 assays relevant to various neurodegenerative disorders, several cephalosporins, including ceftriaxone, showed hits in ALS relevant assays. Efficacy was noted in models screening for compounds that increased expression of the astrocytic glutamate transporter, EAAT2 [1], and in models of superoxide dismutase mediated toxicity. Since completion of the original NINDS screen, it has been shown that ceftriaxone increases EAAT2 promotor activation and subsequent EAAT2 activity in rodent brains. Ceftriaxone also protects motor neurons in culture from excitotoxic cell death [2], and has antioxidant and anti-apoptotic activity [3, 4]. We propose a novel study design strategy of nonstop drug development with multiple intermediate analyses to rapidly apply information on cerebrospinal fluid (CSF) penetration and safety of ceftriaxone to the develoment of a phase III efficacy study [5]. An important first step in the proposed study is to determine the CSF pharmacokinetics of ceftriaxone in subjects with ALS (STAGE 1), followed by a safety and tolerability study for a minimum of 20 weeks (STAGE 2), and then a full efficacy trial (STAGE 3).

Specific aim one (STAGE 1) is to determine the steady state levels and population pharmacokinetics of ceftriaxone in the CSF in approximately 60 subjects with ALS treated with either 2 or 4 grams per day of ceftriaxone or placebo. This information will be used to modify the dosage for STAGE 2 study if necessary, and to determine the final dosage and treatment schedule for the efficacy trial (STAGE 3). Subjects in STAGE 1 will continue into STAGE 2.

Specific aim two (**STAGE 2)** is to assess the safety and tolerability of 2 and 4 grams per day of ceftriaxone in the same subjects with ALS after a minimum of 20 weeks of daily treatment as measured by adverse events, laboratory data and ability to remain on assigned treatment. Information from STAGE 2 will be used to decide whether to continue to a phase III efficacy study (STAGE 3) and to determine the final dosage. Subjects will continue into STAGE 3.

Specific aim three (STAGE 3) is to determine whether chronic treatment with ceftriaxone prolongs survival free of tracheotomy or permanent assisted ventilation and slows rate of decline in function as measured by the ALSFRS-R. The final dosage of 4g (2g bid) per day, for the efficacy study was selected based on the data obtained in STAGE 1 and 2. An additional 540 subjects will be enrolled in the study in STAGE 3.

1.2 Study Overview

Approximately 60 eligible subjects from up to 12 clinical centers will be randomized (1:1:1) in STAGE 1 to ceftriaxone 2 grams per day, 4 grams per day, or color-matched placebo. All 60 subjects will participate in a pharmacokinetic study seven days after starting study treatment and then proceed into STAGE 2 in the same treatment arm assignment. The first intermediate analysis will occur after all 60 subjects complete the pharmacokinetic study. Following this analysis, adjustments in dosage may be made, if necessary. The second intermediate analysis will occur after all 60 subjects have completed a minimum of 20 weeks of treatment. If no dosage adjustments are deemed necessary following the first intermediate analysis, the subjects will continue to receive study drug in their assigned treatment arm until a decision is made on final dosage and treatment schedule for the STAGE 3 efficacy trial. At that time, the subjects assigned to the placebo arm will continue on placebo; and the subjects assigned to ceftriaxone (2 grams or 4 grams/day) will be reassigned to the ultimate ceftriaxone dosage chosen for the efficacy trial (STAGE 3). The additional 540 subjects entering STAGE 3 study will be randomized (2:1) to ceftriaxone or matched placebo.

The co-primary outcome measures for the efficacy study **(**STAGE 3) are survival, measured by time to death, tracheostomy or permanent assisted ventilation, and a change in function, measured by ALS functional rating scale, revised (ALSFRS-R)[6],. Secondary outcome measures include change in vital capacity (VC), evaluation of upper and lower extremity muscle strength using hand-held dynamometry (HHD), quality of life scales (ALS-Specific Quality of Life Scale and Caregiver Burden Inventory), and the safety and tolerability of ceftriaxone in this population. Given the length and size of this study, the study will be conducted with simple outcome measures that can be obtained in the home if needed. This approach will enhance the ease of subject participation and minimize missing data and study resource utilization, but at the same time be sufficient to ensure subject safety and study integrity. The study is designed to identify a modest and clinically significant impact on ALS disease course.

2 BACKGROUND AND SIGNIFICANCE

2.1 Background and Rationale

2.1.1 Clinical Features and Epidemiology of ALS

Amyotrophic lateral sclerosis is a rare degenerative disorder of large motor neurons of the cerebral cortex, brain stem and spinal cord that results in progressive wasting and paralysis of voluntary muscles [7]. The incidence of ALS is currently approximately 2/100,000/year [8, 9] and may be increasing [10]. The lifetime ALS risk is 1 in 600 to 1 in 1000. Even though the incidence of ALS is similar to that of multiple sclerosis [11], the prevalence is only 4-6/100,000 (about 25,000 patients in the United States), due to the higher mortality rate. Fifty percent of ALS cases die within three years of onset of symptoms and 90% die within five years [11]. The median age of onset is 55 years. The cause in most cases is unknown. Age and gender are the only risk factors repeatedly documented in epidemiological studies [12]. There is a slight male predominance (3:2 male to female ratio) in sporadic ALS. No treatment

prevents, halts or reverses the disease, although riluzole use is associated with a slight prolongation of survival [13, 14].

The majority of ALS cases are sporadic (SALS); 10% are familial (FALS). More than 100 point mutations in the gene encoding cytosolic copper-zinc superoxide dismutase (SOD1) have been demonstrated to cause typical FALS [15]. Essential features of ALS are progressive signs and symptoms of lower motor neuron dysfunction (atrophy, cramps, and fasciculations) associated with corticospinal tract signs (spasticity, enhanced and pathological reflexes) in the absence of sensory findings [9]. There is relative sparing of muscles of eye movement and the urinary sphincters. The course is relentless with decline in strength, respiratory function and overall function with time during the active phase of the disease [16]. Natural history studies have determined that age at onset, site of onset, delay from first symptom to entering ALS clinic, and rate of change in respiratory function are significant covariates of survival [17-20]. No study has correlated activity of any surrogate marker with the extent of motor neuron loss as detected by post-mortem examination.

2.1.2 Overview of ALS Pathogenesis

Many causes of ALS have been proposed including toxicity from excess excitation of the motor neuron by transmitters such as glutamate, free radical-mediated oxidative cytotoxicity, neuroinflammation, mitochondrial dysfunction, autoimmune processes, cytoskeletal abnormalities, and aberrant activation of cyclo-oxygenase [21]. It has also been suggested that atypical viral infections may trigger this disease (e.g. enteroviruses or atypical retroviruses) [22-24]. Whatever the cause, it is evident that there are multiple levels of cellular dysfunction as the disease progresses and that programmed cell death is activated in this disease [25-29]. Mutations in the gene encoding SOD1 account for about 25% of cases of FALS or 2-3% of all ALS cases [15]. Forced expression of high levels of a mutant SOD1 transgene causes progressive motor neuron disease in mice and rats [30]. Recent elegant studies in chimeric mice have clearly demonstrated that cell death in motor neuron disease in the mutant SOD1 transgenic mouse model is non-cell autonomous and that the surrounding cells influence survival of motor neurons [31]. Additional genes implicated in ALS-like syndromes include ALS2 which codes for a guanine-nucleotide exchange-like factor [32, 33] and the dynactin gene [34, 35]. Five genetic defects have now been reported to cause FALS [15, 32-37].

2.1.3 Glutamate Excitotoxicity in ALS

Glutamate and aspartate are the predominant excitatory neurotransmitters in the mammalian CNS. These excitatory amino acids (EAAs) activate ligand-gated ion channels that are named for the agonists N-methyl-D-aspartate (NMDA), amino-3-hydroxy-5-methyl-4-isoxazolepropionate (AMPA), and kainate and G-protein-coupled metabotropic receptors. There is substantial evidence that extracellular accumulation of EAAs and excessive activation of EAA receptors contribute to the neuronal cell death observed in acute insults to the CNS. The process known as, ‘excitotoxicity’, also contributes to neuronal loss in chronic neurodegenerative diseases, including ALS [38, 39]. Excitotoxicity is based on altered extracellular concentrations of the EAA, since it is this pool that can be toxic to neurons. The intracellular concentrations of Glu (5-10 mM) and aspartate (1-5 mM) are 1000-fold to

10,000-fold greater than the extracellular concentrations (<1-10 μM). Low extracellular levels of glutamate are maintained by transport into neurons and astrocytes. If glutamate accumulates in the synaptic space, it can exert neurotoxic effects possibly mediated by entry of calcium intracellularly in the motor neurons. This triggers activation of a variety of enzymes including phospholipases and proteases that can lead to cell death [40, 41]. In both *in vitro* and *in vivo* models, excessive concentrations of glutamate lead to neuronal death [42].

Five distinct glutamate transporters have been cloned that express sodium-dependent high-affinity Glu transport, designated GLT-1/EAAT2, EAAC1/EAAT3, GLAST/EAAT1 [43], EAAT4 [44, 45], and EAAT5 [46]. The EAAT (excitatory amino acid transporter) nomenclature refers to the human transporter species while GLT refers to the rodent transporter. There is also evidence for heterogeneity of GLT-1 and GLAST mRNA that originates from alternate mRNA splicing [47, 48]. Expression of EAAT1/GLAST and EAAT2/GLT-1 is generally restricted to astroglia. Expression of EAAT3/EAAC1 and EAAT4 is restricted to neurons, while EAAT5 is restricted to retina [49]. The astroglial transporter EAAT2/GLT-1 is responsible for the bulk of glutamate transport from the synaptic cleft. [50-55].

The expression of EAAT2/GLT-1 is dynamically regulated both *in vivo* and *in vitro*. Although EAAT2/GLT-1 is the predominant transporter in the adult CNS, expression is low early in development and increases during synaptogenesis in both rats and humans [56, 57]. Lesions of neurons, especially loss of presynaptic terminals lead to selective, but transient, down regulation of EAAT2/GLT-1 and GLAST expression [58, 59]. These data suggest that the presence of neurons induces and/or maintains expression of the glial transporters *in vivo*. Several groups have demonstrated decreased expression of EAAT2/GLT-1 and/or GLAST in animal models of acute insults to the CNS, including stroke and traumatic brain injury [49]. Decreases in EAAT2/GLT-1 expression in patients with ALS [60] and in the mutant SOD1 mouse [61] and rat models [62] have been observed. Fisher has recently confirmed that EAAT2 expression is inhibited by TNFα [63], and is modestly stimulated by TGF family members. Importantly, pathways and genes that regulate the EAAT2 promoter may also be an important target for EAAT2 protein expression. Genes have been identified [64] that inhibit EAAT2 expression and are responsible for the inhibition of EAAT2 expression in pure astrocyte cultures. Preliminary data show that increasing expression of EAAT2 protein in experimental systems can clearly result in altered clearance and neuroprotection [64, 65].

There is substantial evidence to indicate that EAA transport plays a significant role in protecting the brain from the accumulation of extracellular EAAs. Selective knockdown of transporter subtypes induces excitotoxicity, pointing to a prominent role of GLAST and GLT-1 in excitotoxicity. Finally, mice deleted of GLT-1 display a unique increased sensitivity to excitotoxic insults and do not survive beyond 20 days of age [51]. Conversely, over expression of glutamate transporters can be neuroprotective. For example, overexpression of EAAT2/GLT-1 can delay onset and prolong survival in ALS mice [65, 66] and prevent motor neuron degeneration *in vitro*. Recent work suggests that over expression of any of the transporters may be neuroprotective either *in vitro* or *in vivo*. Agents that upregulate the EAAT2 transporter might have the potential for a therapeutic effect in ALS by increasing astrocytic uptake of glutamate. This approach to anti-glutamatergic takes a different approach

to current therapeutic strategies that have been directed toward inhibition of neuronal glutamate release, for example by riluzole [13]. We have found that ceftriaxone increases by three to four-fold EAAT2 activity in rodent brains, due to its ability to increase EAAT2 promotor activation [1].

2.1.4 NINDS Drug Screening- Identification of Potential ALS Therapeutics

Screening many small molecules for activity in biological assays is a commonly used drug discovery tool, but has recently been employed to understand biological mechanisms. The Neurodegeneration Drug Screening Consortium (NDSC) was initiated by NINDS to identify compounds that might have therapeutic effects in a variety of neurodegenerative diseases, including ALS. The NDSC involved twenty-six laboratories that screened the same collection of 1040 compounds in 29 different assays relevant to one or more neurodegenerative disorders. Of the compounds, 75% were FDA approved drugs, with the remaining compounds representing various nutritionals and natural products. The assays developed by NDSC members included biochemical assays, cell-free assays for mitochondrial function, cell-based assays, and *in vivo* assays in *Drosophila* and *C. elegans*. Each assay examined a target relevant to neuropathogenesis, including protein aggregation, protein toxicity, excitotoxicity, and apoptosis. At least seven of the assays were relevant to ALS postulated pathways of neuronal degeneration and toxicity. The screening of compounds was investigator blinded, and all compounds were screened at concentrations ranging from 1 to 100μM, depending on the assay. Compounds with greatest potency, usually 10-20 per assay, were further analyzed over a 100-fold range of concentration to test dose-response relationships and determine estimated EC_{50}s. From this screening paradigm, and after normalization and ranking of data, cephalosporin antibiotics were the only class of compounds active in the majority of ALS-relevant assays.

The NDSC chemical library contained 16 cephalosporins. The NDSC data analysis revealed that these drugs were active in assays related to two distinct mechanisms of ALS pathogenesis: glutamate excitotoxicity and mutant SOD1 activity (see Section 2.2 Supporting Data). As described above, the molecular pathogenesis of sporadic and familial ALS is not known. Overexpression of several of the mutant SOD1 proteins results in a mouse and rat model of the disease [30, 67].

The mechanisms of neurotoxicity of the mutant protein which causes cell death is not fully delineated but appears to involve protein aggregation, mitochondrial dysfunction, and heightened excitotoxicity associated with loss of expression of EAAT2/GLT1. In fact, both sporadic and familial forms of the human disease, as well as the transgenic mutant SOD1 models demonstrate loss of the glutamate transporter protein, and reduced functional glutamate transport. Despite the incomplete understanding of the mechanism of motor neuron death in ALS, it was intriguing that multiple members of the cephalosporin class showed strong activity in at least 2 ALS-related assays, measuring protection from SOD1 toxicity and EAAT2 expression [1, 68]. In addition, ceftriaxone when administered to transgenic mutant SOD1 mice prolongs survival [1]. This suggests that glutamate removal may mitigate SOD1 cytotoxicity, or that cephalosporin activity may involve a more general protective mechanism that concurrently affects both pathways. This shared activity of cephalosporins provides a unique pharmacological tool for understanding the

basis of this relationship as well as providing a potential therapeutic option aimed at several pathways important in ALS pathogenesis.

2.1.5 Rationale for Choosing Ceftriaxone

Ceftriaxone was effective in the *in vitro* assays that measured glutamate transport [1] and protection from SOD1 toxicity. Ceftriaxone has also been reported to protect against radiation-induced neurodegeneration in an *in vitro* model [3]. The mechanism for neuroprotection in these systems remains unknown. A literature review and discussions with infectious disease consultants has confirmed that none of the orally administered cephalosporins are likely to achieve CNS penetration or significant cerebrospinal (CSF) levels [1]. Ceftriaxone has the longest serum half-life of all currently available cephalosporins and is thought to have effective penetration into the CSF [69] (Appendix 1). It is likely that we can achieve CSF levels in the range of effective concentrations found in the EAAT2 gene expression assay (>1 μM).

Ceftriaxone General Properties. Cephalosporins are beta-lactam antibiotics that differ from the penicillins in that the beta ring is a six-membered dihydrothiazine ring. They are typically used for the treatment of septicemia, pneumonia, meningitis, and urinary tract infections. Some cephalosporin antibiotics, including ceftriaxone, can penetrate through the blood brain barrier under normal conditions; this is enhanced in the case of bacterial meningitis. The usual adult daily dosage of ceftriaxone is 1 to 2 grams given once a day or in equally divided doses twice a day. The total daily dosage should not exceed 4 grams per day (Appendix 1). Ceftriaxone may be administered intravenously or intramuscularly.

Ceftriaxone Pharmacokinetics. Following a single intravenous (IV) dose of 2 grams of ceftriaxone in healthy subjects, the serum half-life is approximately 8.5 hours. The half-life is independent of dose. Ceftriaxone is highly protein bound (83-96%), primarily to plasma albumin, and has an apparent volume of distribution (Vd) of 9-14 liters. Nonlinear protein binding has been demonstrated for ceftriaxone, resulting in an increase in the free fraction of ceftriaxone in plasma with increasing drug dosage [70]. Therefore an increase in its free fraction in plasma was found when a higher dose was given. As a result, higher CSF levels after once daily dosing as compared to twice daily dosing (with the same total dose) are expected [71]. Steady state levels are reached within three days. Thirty-three to sixty-seven percent of a dose is excreted in the urine as unchanged drug, and the remainder is excreted in the bile [72]. The total clearance is 20 ml/min, with renal clearance contributing 7 ml/min. The clearance of ceftriaxone is lower in patients with mild renal insufficiency than in patients with normal renal function and the half-life increases with declining renal function (to 13.1 hours) [72-75]. Dosage adjustments in patients with renal or hepatic dysfunction are not necessary with ceftriaxone up to 2 grams per day [72]. Plasma ceftriaxone concentration measures are not influenced by prior administration of probenecid [74, 76, 77].

Antibiotics enter CSF predominantly via passive diffusion down a concentration gradient. The major determinant of CSF penetration is lipid solubility. Beta lactam antibiotics enter CSF through paracellular pathways. Their transport depends on the opening of tight junctions and peak CSF concentrations are relatively delayed. Animal studies suggest that ceftriaxone can

be transferred from blood to the extracellular space of the brain in part by a low-capacity facilitated diffusion system. Transport from the CSF back into blood by the choroid plexus transport system is relatively inefficient compared to other antibiotics, resulting in a decreased rate of clearance from the CNS [78]. Delayed entry into the CSF and the slow clearance from CSF results in a delayed peak CSF concentration in comparison with plasma. This also results in a longer CSF half-life in the CSF. Antibiotic concentrations in the brains of humans and animals have usually been estimated by measurement of CSF levels. For certain drugs, including ceftriaxone, CSF is pharmacokinetically indistinguishable from other brain regions. Using *in vivo* micro dialysis in rats, extracellular CNS concentrations of ceftriaxone were comparable to CSF levels [79].

Animal Studies. Rodent studies suggest that ceftriaxone enters the CNS after IV administration. In rats, detectable levels were found in the brain after IV administration of C14 labeled ceftriaxone [80]. Mice treated with 25 mg/kg of ceftriaxone for at least four days had brain mean peak levels of 83 μg/ml [81]. Ceftriaxone levels in CSF of dogs with healthy meninges 90 and 240 minutes after IV injection of 50 and 100 mg/kg were on average 0.37 and 1.22 μg/ml respectively. The peak CSF concentration was reached 150 minutes after the beginning of the antibiotic administration (60 minute infusion) and the peak plasma concentration after 45-60 minutes. High levels in CSF persisted more than four hours after the dose was administered [82].

Human Studies. Most CSF studies of ceftriaxone were performed in patients with meningitis; and often after patients received only one to a few doses of ceftriaxone. There have been very few studies of CSF penetration of ceftriaxone in humans without inflamed meninges. Available data suggest that at 2 grams per day, levels in CSF can be achieved that compare favorably to those effective in *in vitro* models (Appendix 1). Cerebrospinal fluid ceftriaxone levels in subjects without meningitis range from 0.18 to 3.5 μg/ml (0.33 μM to 6.33 μM) depending on study, dose, and timing of CSF sampling [83-87]. The molecular weight of ceftriaxone (free acid) is 552.5. Therefore, 1 μM is equivalent to 0.5525 μg/ml and 1 μg/ml is equivalent to 1.81 μM. Once present in CSF, concentrations of ceftriaxone are well maintained over time. The half-life of ceftriaxone in CSF is approximately twice that seen in blood in both rabbits (7-8 hours in CSF, three hours in blood), and humans (16.8 hours in CSF, 8.5 hours in blood) [78, 84]. Steady state CSF levels of ceftriaxone after 4 grams/day treatment are not known for subjects without meningitis. Ceftriaxone is not metabolized in CSF and its concentration and half-life in CSF depend on the balance between drug penetration and elimination through the blood brain barrier. Ceftriaxone is not readily transported out of the CSF [80]. This was demonstrated clinically in a single patient who erroneously received an intrathecal injection of 800 mg of ceftriaxone [88]. Once injected into the CNS, exceptionally high levels persisted for prolonged periods of time, even after attempts to remove it by CSF exchange. The pharmacokinetic data from STAGE 1 was reviewed by the study Data Safety and Monitoring Board (DSMB). Both dosages (2 and 4 g per day) achieved the target trough CSF level of 1 uM. At a dosage of 4 grams/day, pharmacokinetic study showed that CSF levels were maintained above our criterion level for at least 60 hours. This supports our plan to allow up to 2 day drug holidays depending on patient burden without CSF levels dropping below 1 uM.

Dosage and Therapeutic Range. The maximum daily dosage allowed by the FDA is 4 grams per day in divided doses. It is not known if the mechanism by which the drug crosses the blood brain barrier is saturable or if 4 grams per day exceeds the saturable point. One study assessed CSF levels in seven patients with bacterial meningitis who received 4 grams of ceftriaxone once a day. The average CSF level three to five hours post dose was 27.8 μg/ml; 18-24 hours post-dose average CSF levels was 7.5 μg/ml [89].

2.1.6 Experimental Therapeutics in ALS.

Riluzole, a drug that has multiple mechanisms of action including inhibition of release of glutamate at pre-synaptic terminals, was reported in two controlled studies to extend survival by three months (about 11%) in ALS although without a concomitant improvement in strength [13]. This is currently the only FDA approved agent for use in ALS. Trials of CNTF [90, 91], gabapentin [92, 93], BDNF [94], Xaliproden (Sanofi), topiramate [95], Celebrex [96]and creatine [97, 98] were shown to be ineffective in treating ALS. Talampanel and lithium are currently being tested in clinical trials.

There are many approaches to choosing therapies to develop in ALS. To better understand the biology of ALS, various laboratory-based models have been employed. Models of disease initially consisted of pure motor neurons cultured *in vitro*, and slices of the spinal cord – a more complex, yet still *in vitro* paradigm to study motor neurons [2]. These systems identified and/or validated the initial approaches to ALS therapies, including most of the trophic factors considered in ALS and riluzole. The development of transgenic mice and rats expressing mutant forms of SOD1 [30, 99, 100] has provided a valuable animal model of the disease for understanding pathways that can lead to motor neuron cell death. However, the predictive value of the model with regard to the identification of drugs that are efficacious in human ALS is not known [39]. In hopes of finding agents/approaches capable of altering the disease, both academic and pharmaceutical industry labs are now actively pursing drug therapies, gene therapies and even stem cell therapies. These efforts have already resulted in the study of more than 60 different therapies in ALS mice. Most, at least from the published literature have extremely marginal effects. However, in spite of their potential, the transgenic mice have not been entirely predictive of human trial outcomes. Creatine chronically administered before clinical disease onset to the transgenic ALS mouse substantially delayed onset of disease and prolonged survival by 18% in the mice. Unfortunately, two independent clinical trials of creatine at different doses – one in Europe [97] and another that we conducted in the US - have failed to demonstrate a clinical benefit [98]. These data clearly demonstrate an important point: all pre-clinical models have inherent limitations. Dose equivalency is one area in which the relationship between effects seen in the transgenic mouse are not necessarily predictive of a similar effect in human disease. The transgenic mouse was engineered to have early disease onset and rapid course by greatly overexpressing the human mutant SOD1 gene. Therapeutic efficacy in this model is therefore not directly translatable to human disease, where the mutant gene product is not artificially overexpressed. In addition, although FALS is similar to sporadic ALS with regard to a wide range of cytotoxic events, including evidence for excitotoxicity (e.g. loss of glutamate transport protein), oxidative injury, markers/genes reflecting programmed cell death, and neuroinflammation, total equivalency between sporadic and familial disease has not been demonstrated.

The lack of a clear predictive relationship between efficacy demonstrated in the transgenic mouse model and human disease raises the question of whether every drug considered for human trial should first be evaluated in the mouse. The predictive power of the multiple pathway assays performed in the NINDS neurodegeneration screen described above may have equal or greater predictive power than a single mouse study. Most drugs that come from the pharmaceutical industry, in fact, are derived and decided upon through multiple *in vitro* assays. A review of previous and currently planned studies in ALS by large pharmaceutical companies show that few rely on the transgenic ALS mice to make decisions regarding clinical trials (e.g. xaliproden, riluzole, Novartis TCH386, Exon-Hit, IGF-1) but instead rely on pre-clinical assays for trial decisions. Most importantly, to date, no company has used the large battery of ALS –relevant screening assays, present in the NINDS screen, to evaluate their products. For this reason, the assay results may reflect one of the most complete pre-clinical ALS-relevant measures.

2.1.7 Significance

Despite recent critical advances in understanding the pathogenesis of ALS, this remains an untreatable and uniformly lethal disease. The novel and multiple activities of cephalosporins against a variety of potential disease mechanisms provide a unique opportunity to evaluate a single agent aimed at several pathways important in ALS pathogenesis. The preliminary data demonstrating beneficial effects of cephalosporins in ALS related assays provide strong incentives to undertake a trial in humans with ALS. Any compound proven to slow the course of the illness will be of immediate clinical importance; moreover, a positive outcome will enhance our understanding of the underlying biology of motor neuron diseases and may lead to further development of effective treatments.

2.2 Supporting Data

2.2.1 Ceftriaxone Protects Against Motor Neuron Degeneration

Ceftriaxone was originally reported to be neuroprotective in an organotypic slice of postnatal rat spinal cord model of motor neuron degeneration [2]. In this model, the blockade of glutamate transport with the competitive inhibitor threo-hydroxyspartate leads to a dramatic loss of motor neurons over a 4-week period (Figure 1). When ceftriaxone 10 or 100 µM was added to the culture medium, there was clear neuroprotection against motor neuron degeneration. As part of the NDSC, the compound library was screened in an organotypic spinal cord culture for agents that increase EAAT2/GLT-1 protein expression (Figure 2). Four of the 8 drugs found to increase EAAT2/GLT-1 expression were cephalosporins. These cephalosporins showed a 5-7-fold increase in EAAT2 protein with EC_{50}s of 3.5-5 µM. The shared activity of these drugs suggests that the common structural elements of cephalosporins are responsible for the upregulation of EAAT2 expression. In three additional assays that were based on mutant SOD1 cytotoxicity in various *in vitro* neuronal cultures, 10 cephalosporin antibiotics, including ceftriaxone, were found to be highly cytoprotective, with EC_{50}s of 50-75 µM.

Two of the *in vitro* assays based on mutant SOD1cytotoxicity expressed mutant SOD1 by replication-deficient adenovirus, one in SHSY5Y human neuroblastoma cells [101], and the

other in Pc12 cells [102]. In both, the mutant SOD1 sufficed to kill without an additional oxidative stress. A third SOD1 mediated toxicity assay employs undifferentiated mouse neuroblastoma neuro 2A (N2A) cells that have been stably transfected with human SOD1 that is either wild type or mutant (G37R, G41D and G85R) plated in a miniaturized format (96 and 384 well microtiter plates). Cyclosporin A induces cell death in this cell line; and the assay screens for compounds that rescue the undifferentiated cells. The mechanism of action of the screened cephalosporins is not known. The cell toxicity pathways that are "activated" in these assays are many- including oxidative injury, protein aggregation, mitochondrial injury, excitotoxicity and programmed cell death.

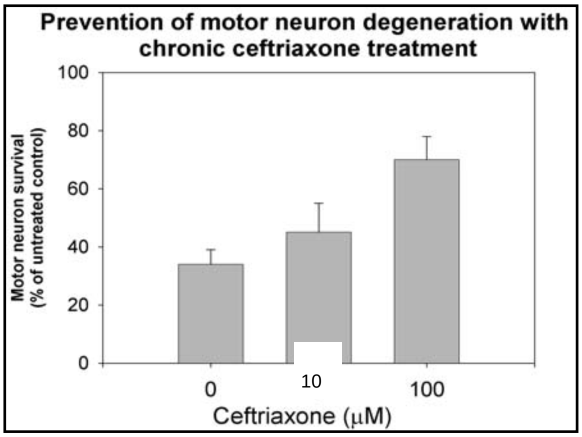

Figure 1. Ceftriaxone prevents motor neuron degeneration in vitro. Chronic treatment of spinal cord organotypic cultures with the glutamate transport inhibitor threo-hydroxyaspartate leads to loss of >50% motor neurons (point 0, above). Co-treatment with ceftriaxone prevents this excitotoxic loss of motor neurons.

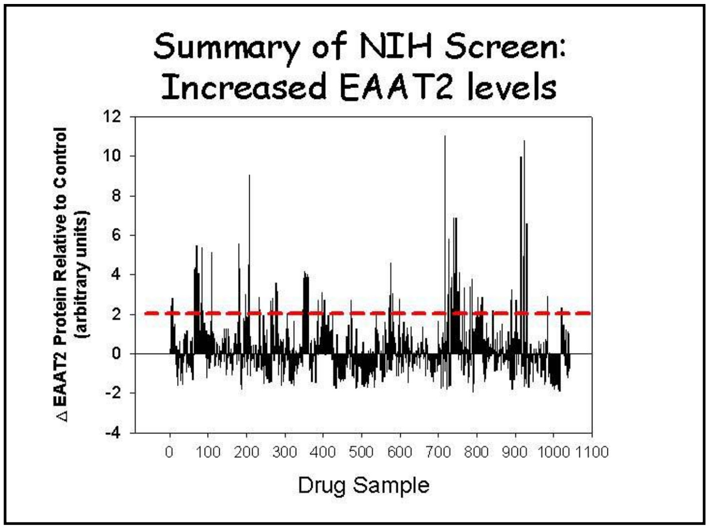

Figure 2. Screening results from the NIH-NINDS Custom Collection screen of 1040 FDA approved compounds. The height of the bars reflects increased EAAT2 protein expression relative to untreated controls. Each drug measurement included at least one untreated control and one known positive control.

As in any high throughput-screening assay, the outcomes of the hit may often involve a chemical/biochemical action of a compound heretofore unknown. One possible mechanism for protecting against glutamate toxicity would be an increase in synaptic clearance of glutamate and a subsequent diminution of excitotoxicity. In fact, additional preliminary data now strongly suggest that cephalosporins increase EAAT2 activity and thereby enhance synaptic glutamate clearance. As shown in Figure 3, ceftriaxone can activate the EAAT2 promoter in astroglial cells lines. Furthermore, ceftriaxone also increases glutamate transporter EAAT2 protein expression *in vivo* (Figure 4). When administered peripherally to rodents at clinically relevant doses (200 mg/kg), ceftriaxone increases brain expression of the glutamate transporter EAAT2, three to four fold. Finally, preliminary studies suggest that ceftriaxone, by increasing glutamate transport, can protect against neuronal injury in an *in vitro* model of ischemic injury.

The cephalosporins were effective at much lower concentrations in the EAAT2 protein expression assay, compared to the cell toxicity assays. Accordingly, it is difficult to estimate

the target concentration that might be effective in humans. The EC_{50} for ceftriaxone and other cephalosporins from the NINDS drug screening initiative was 3.5 to 5µM in the EAAt2 expression assay. Ceftriaxone was effective in increasing EAAT2 promoter in primary human astrocyte cultures at concentration of 1.0 µM, but not at 0.1 µM. It may be that the EAAT2 assay is more physiological than the SOD1-mediate cell toxicity screens because the EAAT2 assay screens for compounds that assess promoter activity in an otherwise normal setting. The mutant SOD1 mediated assays screen for neuroprotection from acute insults (high level SOD1 expression or high level glutamate) and therefore may be less relevant to a slowly progressive human disease state; the acuteness and intensity of the toxic stress in these assays may require higher concentrations of drug to show an effect. Administration of ceftriaxone to a transgenic mouse model of ALS prolonged survival by two weeks [1].

Figure 3

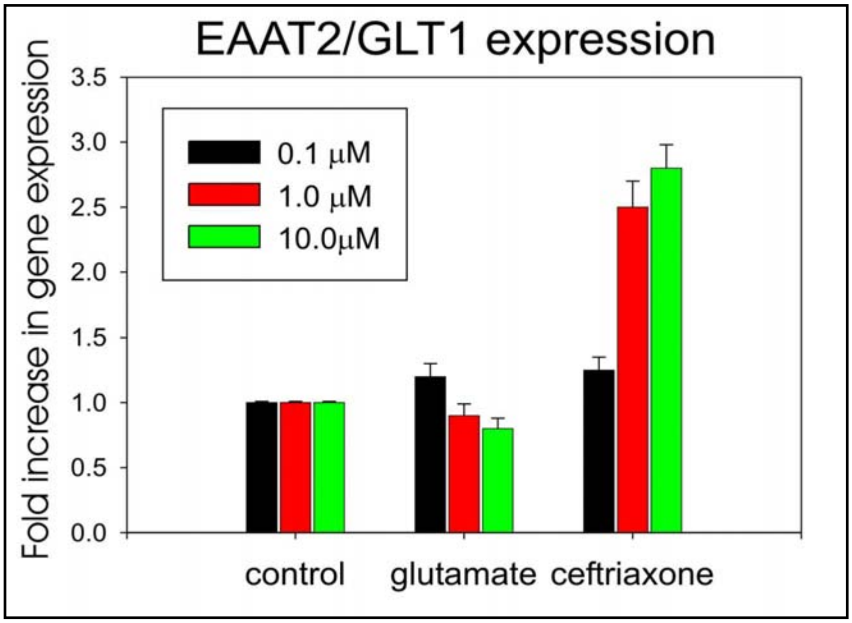

Figure 4

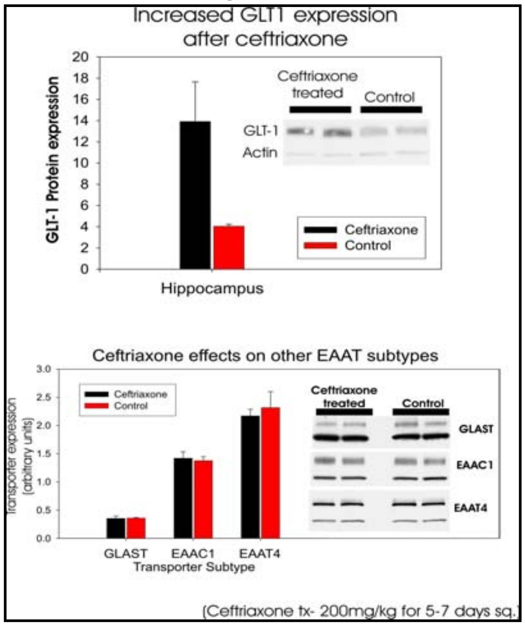

Figure 3. Ceftriaxone treatment of primary human astrocytes cultures leads to a 3-fold increase in EAAT2 promoter – luciferase reporter activity. Glutamate and other amino acids (not shown) had no effect.

Figure 4. Ceftriaxone administration to rats (200mg/kg ip, x 5 days) leads to a 3 to 4-fold increase in GLT1 protein expression in brain, but has no effect on the astroglial glutamate transporter GLAST, or the neuronal glutamate transporters EAAC1 or EAAT4.

2.2.2 Safety and Clinical Use of Ceftriaxone in Humans

Ceftriaxone is generally well tolerated and severe adverse events have only rarely been reported. Over 100 million people have received ceftriaxone for the treatment of a variety of infections. Few patients have received ceftriaxone for more than six weeks. Ceftriaxone is not approved by the Food and Drug Administration (FDA) for treatment longer than 4-6 weeks. Toxicities of ceftriaxone are similar to those reported with other cephalosporins and include pseudomembranous colitis, super-infections, local pain on administration, phlebitis, fever,

pruritis, rashes, anorexia, nausea, vomiting, diarrhea, and abdominal pain. There is no evidence of teratogenicity in rodent study or in primates. There are no adequate studies in pregnant women.

Across many studies, the most frequently observed adverse events during treatment with ceftriaxone are hematological, including eosinophilia (6%), thrombocytosis (5.1%), leukopenia (2.1%), and thrombocytopenia (1%); however these adverse reactions have been reversed when drug administration was stopped [103]. Gastrointestinal adverse events during ceftriaxone therapy, primarily diarrhea (2.7%), are common. Ceftriaxone is also excreted via the bile leading to high biliary concentrations of the drug. Biliary adverse effects include the formation of biliary sludge leading to biliary obstruction, cholecystitis, choledocholithiasis, and pseudolithiasis. Prolonged use of ceftriaxone may result in overgrowth of nonsusceptible organisms. Neutropenia has been reported with ceftriaxone use. In one series, the mean time to develop neutropenia was 21 days (range 8-25 days). It was reversible upon discontinuation of the medication. The frequency of hypersensitivity skin reactions to ceftriaxone is between 1 and 3%; anaphylaxis is rare. The risk of anaphylaxis may be increased with a history of allergy to penicillin and is certainly increased with a history of allergic reaction to cephalosporin. Ceftriaxone is contraindicated in patients with known hypersensitivity to penicillins or cephalosporins. Nonconvulsive status epilepticus has been rarely reported with ceftriaxone use and is more common in patients with renal failure [104].

All cephalosporins are potentially nephrotoxic, especially at high doses [105]. It is recommended that renal function and electrolytes should be monitored following very large doses of cephalosporins, or if administered for an extended period of time. The most common nephrotoxic effects of cephalosporins are acute allergic interstitial nephritis (AIN) and acute tubular necrosis (ATN) [106]. Clinically, acute allergic intersitial nephritis can present with rash, fever, arthralgias, eosinophilia, or just isolated acute renal dysfunction with a rising BUN and creatinine. Proteinuria is generally less than 2 grams/day. Urinalysis may be remarkable for white blood cells and white blood cell casts, (with a negative urine culture) and the presence of eosinophils by Wright stain. Renal biopsy typically shows a marked interstitial inflammatory infiltrate with lymphocytes, plasma cells, and eosinophils, as well as edema with focal tubular damage. On occasion, epitheliod granulomas with giant cells may be seen within the interstitium. Generally, symptoms occur 2 weeks after initiation of therapy, although this has been reported to vary from 2 to 60 days [107]. Discontinuing the offending agent plus the addition of steroids may be indicated if renal dysfunction is severe or oliguria occurs. The second pattern of injury that can be seen with cephalosporins is acute tubular necrosis (ATN). This can be manifested by the presence of pigmented granular casts on microscopic examination of the urinary sediment, as well as a rising blood urea nitrogen and serum creatinine. This process is generally reversible if the drug is discontinued [106].

There are only a few published studies of ceftriaxone administered at 4 grams per day [108, 109]. Treatment duration was between 10 and 14 days. No serious adverse events were reported. Twenty-three patients with clinically active late Lyme disease were randomized to intravenous treatment with either penicillin or ceftriaxone (2 grams bid; N=13) for 14 days. After 23 patients were treated, they prospectively treated an additional 31 patients with ceftriaxone; 17 with 4 grams per day, and 14 with 2 grams per day for 14 days. Ceftriaxone was generally well

tolerated. None of the patients experience serious side effects, but mild to moderate diarrhea developed in about half of those receiving 4 grams per day and in about 10% of those treated with 2 grams per day [110].

Long Term Treatment with IV Ceftriaxone

Human Studies. Several studies have been published on the use of ceftriaxone for longer than six weeks of treatment [111-118]. Frequently, patients receive multiple (up to five) courses of treatment. These published reports are specific to the treatment of Lyme disease and osteomyelitis. However, a growing number of patients with ALS are currently receiving chronic ceftriaxone treatment. Neither the published reports nor the direct communication with physicians treating patients with long-term ceftriaxone have reported any adverse events. The results of the published studies can be reviewed in the Clinical Investigator Brochure, section 5. Correspondence with physicians who treat Lyme disease has revealed gallstones and biliary sludging to occur in approximately 10% of treated patients (personal communication with S. Donta, MD, R Stricker, MD, M. Cichon, MD, A. Leonetti, MD, and G. Bach, MD from May to July, 2004). Those physicians that treat the cholelithiasis with deoxycholic acids have seen favorable results. No case of renal failure has been reported by these physicians.

The study DSMB reviewed unblinded safety and tolerability data from STAGE 1 and 2. Both 2 and 4 grams/day of ceftriaxone were tolerable at 20 weeks. In STAGEs 1 and 2, the risk of symptomatic biliary events overall was 26%. Data from STAGES 1 and 2 suggested that ursodiol was helpful in management of cholelithiasis and biliary sludge events and was safe and well tolerated. Ursodiol did not have an effect on ceftriaxone blood levels.

26-Week Chronic Toxicology in Baboons. A 26-week study of IV ceftriaxone in baboons was performed by Roche Pharmaceuticals. Thirty juvenile baboons (3/sex/group) received IV ceftriaxone at doses of 0 (saline, Group 1), 30 (Group 2), 150 (Group 3), 400 (Group 4) and 700 mg/kg (Group 5). No renal or biliary adverse events were noted in groups 1 and 2. Minimal nephropathy was observed in one baboon in the group that received 150 mg/kg/day (5 times the human dosage of 2000 mg/day or approximately 30 mg/kg/day). Moderately severe nephropathy was observed in group 4 (four of six baboons) and group 5 (six of six baboons). The nephropathy was characterized by necrosis of the tubular epithelium, inflammatory infiltrate and fibrosis of the interstitium. A treatment related increase in absolute kidney weight was noted in groups 4 and 5. Treatment related gross pathology changes, which included enlarged, roughened and diffusely pale kidneys; these changes happened sporadically in groups 4 and 5. Renal failure contributed to the death of one baboon in group 5. Baboons treated at the two highest dosages (400 and 700 mg/kg) also had gall bladder sediment [119].

As a result of the nephrotoxic findings with long-term high dose treatment in baboons, Dr. Nina Rubin, a renal consultant and expert, is a member of the steering committee, aggressive renal safety monitoring will occur for each subject, and each site is required to have a renal consultant associated with the study. In addition, because only one animal chronic toxicology study has been conducted, the FDA has required that we conduct a six-month IV rodent chronic toxicology study that was run concurrently with the human study. Further details are found in the Clinical Investigator's Brochure for the study.

6-Month Subcutaneous Toxicology Study in Rats. A 6-month subcutaneous toxicology study was performed to evaluate the potential toxicity and toxicokinetics of ceftriaxone following once or twice daily subcutaneous injection to rats for 6 months, followed by a 1-month recovery period.

Dosage levels were selected based on the toxicity data from the previous 2-week study in rats using the same formulation and route of injection. Following dosage levels were used:

1.0 g/kg s.c.	twice a day, 8 hours apart
1.0 g/kg s.c.	once a day
0.5 g/kg s.c.	once a day
0.25 g/kg s.c.	once a day
Control Water	twice a day, 8 hours apart

No ceftriaxone-treated animals died during the study. However animals administered 1 g/kg twice daily appeared to be in distress due to severe irritation at the injections sites. Dosing was stopped at this level on day 49. Swelling and thickened skin was observed at all dose levels but the discoloration at the injection sites resolved during the recovery period. The irritation at the injection sites led to blood loss and iron deficiency anemia in Groups 4 and 5. There were decreases in red cell parameters and increases in reticulocyte counts that trended towards more normal values at the end of the recovery period. As expected, there were increases in cecal size that returned to normal during the recovery period. There was no evidence of ceftriaxone remaining at the injection sites or being precipitated in the liver or kidneys.

Based on the evaluation of this study the no-observed-adverse-effect dose level was 0.5 g/kg/day [120].

2.2.3 Risks of Central Venous Catheters

In the United States, an estimated five million central venous catheters (CVC) are placed every year [121]. Risks associated with their use vary by catheter type, location of insertion site, hospital size, service and duration of placement [121]. Potential adverse events include local and systemic infection, catheter thrombosis, deep vein thrombosis, pulmonary embolus from either air or thrombosis, anatomical compression of the catheter, pneumothorax, accidental arterial perforation, bleeding, mechanical breakage and accidental removal of the catheter. Some of these complications are more likely to occur at either the time of placement, immediately post placement or with long term placement.

Tunneled catheters, like the Hickman catheter, have a low incidence of serious complications when they are used in the community setting. Rates of systemic infection in the community are 0.34 with each 1000 catheter days [122]. This compares favorably with the CVC bloodstream infection for the inpatient settings, where the incidence is between 2.9 to 5.9 for every 1000 catheter days [123]. Bloodstream infections carry an estimated mortality risk of 10% with each occurrence [124]. Systemic infections that occur early after placement, while the wound remains unhealed, are frequently the result of cutaneous organisms that have migrated along the tunneled

catheter, while infections resulting from contamination of the catheter hub are more likely to occur with prolonged catheter use [125-129].

Tunneled catheters in the homecare setting have a low incidence of thrombosis when compared to other CVCs. They occur at a rate of 0.6 for every 1000 hours of use [122]. Thrombosis of the catheter, though not necessarily a serious complication in itself, poses an increased risk if it results in a deep vein thrombosis or a pulmonary embolus. The potential for developing a pulmonary embolus after the formation of an upper extremity deep vein thrombosis is between 12 – 36% [130]. The risk of an air embolus has been assessed at between 0.6-0.8%, with most being small and asymptomatic. Serious adverse effects from air embolus are infrequent [131]. Thrombosis, in general, has an increased risk of developing during the first week after placement and in catheters that have been in place for greater than 30 days [122, 132]. Though the formation of thrombin sheaths within the catheter occur as high as 66% of cases, only 6% of these develop obstructive symptoms. Most instances of thrombosis do not carry significant morbidity and can be relived with the use of a thrombolytic agent [133, 134]. However, the likelihood of thrombosis redeveloping is as high as 79% following the first de novo incident [135].

Other adverse effects directly related to the use of a tunneled catheter include mechanical problems that can result in rupture, cracking or accidental withdrawal of the catheter. In addition, catheters that are placed in the subclavian vein can encounter “pinch off syndrome,” due to anatomical compression. The risk of pneumothorax is primarily a risk related to catheter introduction and occurs in 0% to 3.3% of radiological placements [136]. This lower rate of occurrence may be associated with the use of the jugular vein [131]. The risk of accidental arterial puncture during placement ranges from 1% to 8.4%. Again, the lower rate may be associated with using the jugular vein [131]. Overall, bleeding incidences with the use of tunneled catheter occur 0 to 1.7% of the time [136]. Stenosis of the vein used for catheter access, occurs at a higher rate of 50% for the subclavian vein, compared to 10% when the jugular vein is used [137].

With the widespread use of CVCs, the Centers for Disease Control (CDC) has published guidelines for the prevention of catheter related infection since 1996. These guidelines are supported by evidence-based studies. A working group of professional organizations representing disciplines in critical care medicine, infectious disease, health-care infection control, surgery, anesthesiology, interventional radiology, pulmonary medicine, pediatric medicine and nursing reported their findings in Guidelines for the Prevention of Intravascular Catheter-Related Infections in the Morbidity and Mortality Weekly Report in 2002 [123]. Those recommendations in catheter placement, education and maintenance form the basis of maintaining and monitoring safety throughout this study.

All the recommendations to minimize cutaneous organism contamination proposed by the CDC related to catheter material, catheter cuff, hand washing, aseptic technique, skin antisepsis and exit site dressing will be observed. These include the use of alcohol based hand hygiene, the use of sterile and non-sterile gloves in catheter and exit site care, maintaining aseptic technique, disinfection and exit site care, the use of transparent or gauze dressing and following prescribed frequency of dressing and catheter cap changes. The Hickman catheter to be used has a single

lumen and is constructed of polyurethane with a silver ion antibacterial cuff. In catheter filters, the use of cutdowns, over the wire catheter replacement, routine catheter replacement and the routine use of antiseptic ointment will be avoided, as the CDC recommends. The use of an antibiotic lock prophylaxis or of a prophylactic anticoagulant are measures that will not be included in this study. The insertion process and dressing change frequency will be carefully documented. Training will be provided for all personnel who will be involved in catheter care and their educational process documented. Competency in catheter care will be demonstrated by all personnel involved in this process.

The recommendation to avoid the use of the jugular vein in favor of the subclavian vein was weighed against the anticipated long-term use of the catheter. Risks of vein stenosis, pneumothorax, arterial puncture, and “pinch off syndrome” are increased with the use of the subclavian vein. There are also fewer procedure complications with the use of the jugular vein [131, 136, 137]. These factors were weighed against the higher likelihood of infection risk with the use of the jugular vein site [123]. Jugular vein access, both overall and long term, poses fewer risks and will be the site used for this study.

The risk of infection will be reduced as much as possible by following the safety recommendations of the CDC. Education of the family and patient is key to reducing adverse events. Through early recognition and intervention, developing problems can be mitigated. Each subject and caregiver will be instructed in catheter care and maintenance upon acceptance into the study and throughout the study. This will include a didactic video presentation, individual personal instruction, individual practice sessions on a Hickman catheter and patient model, testing for competency in knowledge content and for the physical skills required for catheter management. On a quarterly basis, subjects and caregivers will be re-educated on the care of the catheter. Procedures will be reviewed and documentation of correct understanding or the procedures will occur. These educational objectives will be reinforced through a personal home instructional video, written instructions and illustrations, availability of a visiting nurse visit and continued education and reinforcement with each site visit. Subjects and their caregivers will have instruction in what constitutes an emergent situation and the course of action to take. Study nurses and physicians will encourage phone contact and be available on call to answer questions and concerns, and if need be, to order visiting nursing visits, site visits or emergent visits. Each site will be required to have an infectious disease specialist available for clinical consultation, and every subject who shows signs of infection will be referred to an infectious disease specialist for clinical consultation.

2.2.4 Risks of Pediatric Multivitamin Solution

There have been rare reports of the following side effects associated with the pediatric multivitamin (MVI) solution that will be used as the placebo treatment: rash, erythema (redness of the skin), pruritis (itching), headache, dizziness, agitation, anxiety, diplopia (double vision), urticaria (hives), periorbital and digital edema (swelling around the eyes and swelling of the fingers). Multivitamin solution was used as the placebo for a prior placebo-controlled clinical trial of ceftriaxone in Lyme disease [138].

2.2.5 Potential Benefits

If ceftriaxone is shown to have clinical efficacy with minimal to moderate risks, then the potential benefit from this study is large and outweighs the risks. Many measures will be in place to minimize risks of both the study drug and the mode of delivery. These include education of clinical trial staff, subjects and their caregivers on the potential risks of study drug and central venous access and how to minimize these risks, inclusion of experts in nephrology, infection disease and central venous catheters on the Steering Committee, and requirement for each site to have a nephrologist and infectious disease specialist available for consultation.

3. STUDY DESIGN

3.1 Study Design Overview (Figure 5)

Using a three-stage sequential, non-stop drug development design, we will determine optimal dosage, safety and efficacy of ceftriaxone in ALS. This design was chosen to expedite drug development while safely testing a new therapeutic agent in ALS. In STAGE 1, approximately 60 subjects will be randomized to one of three groups: ceftriaxone 2 grams daily, 2 grams twice daily or placebo. After seven days of treatment, they will be admitted to institutional GCRC or clinical research centers for a plasma and CSF pharmacokinetic study. All subjects will continue treatment after the STAGE 1 study and enter STAGE 2 to determine safety and tolerability after 20 weeks of daily treatment. If necessary, dosage adjustments may be made based on the analysis of data from the pharmacokinetic study during STAGE 1. After 60 subjects have completed at least 20 weeks of treatment, a decision will be made whether to continue to a phase III efficacy study (STAGE 3), and the single dosage to be used. An assessment will be made as to whether subjects can be given a safe dosage that will produce CSF levels reasonably expected to produce a biologic effect. If the decision is affirmative, the study will be expanded to a total of 600 randomized subjects at approximately 70 centers in US and Canada. Subjects in the STAGE 1 and 2 studies will continue on their assigned treatment arms until a decision on the STAGE 3 trial is made. They then will roll over into the larger phase (STAGE 3) study with subjects on active treatment in STAGE 1 and 2 being combined into the STAGE 3 portion of the study. New subjects will be randomized in a 2:1 fashion to receive treatment of ceftriaxone or placebo. Subjects will remain in study until 52 weeks after the last subject is randomized. The co-primary outcome measures for the phase III study will be survival free of tracheostomy or permanent assisted ventilation and the ALSFRS-R. Secondary outcome measures will include change in VC, evaluation of upper and lower extremity muscles using HHD, evaluation of quality of life, and the long-term safety and tolerability of ceftriaxone in this population.

The rationale for combining phase I-II-III for this study is that ceftriaxone is the most promising human drug from a large drug-screening program conducted by the NINDS. Since ceftriaxone is already an approved drug the only major phase I issue is its concentration in the CSF. The phase I-II-III study that we propose will speed the testing of this drug by at least two years as compared to separately funded and conducted phase I/II and III studies. Given that ALS is currently a uniformly fatal disease the rapid testing of such a promising agent is necessary. AIDS researchers came to a similar conclusion in 1990 in designing trials for AIDS [139].

Survival and ALSFRS-R were chosen as co-primary outcome measures. The two measures assess complementary and critical aspects of ALS progression. Survival remains the gold standard measure for an intervention whose goal is to modify disease progression. While several functional outcomes (including ALSFRS-R and pulmonary function) correlate with long term survival, they remain unproven surrogates for survival and for disease modification in general. Ceftriaxone has the potential to be a disease modifying treatment, and thus we believe it is appropriate to study sufficient numbers of patients for prolonged periods to provide a realistic chance to achieve a meaningful difference in survival. Survival is also the most robust of endpoints; it can be reliably determined in all subjects even if active participation in the study is terminated. As this study is designed as intent to treat, the uniform ability to obtain a survival outcome reduces the possibility of missing data.

Survival is not the only meaningful outcome for subjects with ALS, however. Functional measures have the potential to show meaningful differences with shorter study duration and fewer patients than what is needed to demonstrate a survival difference. The ALSFRS-R is a validated, reproducible, and reliable functional measure that assesses subject function across four domains: bulbar, fine motor, gross motor, and respiratory. It can be performed both in person and by telephone, and is highly correlated with survival. We have chosen ALSFRS-R as a co-primary outcome because it is s a more sensitive measure of change in patient function than survival, and because meaningful differences can be detected in a shorter time period than is necessary to evaluate survival. The ideal outcome in this study is that significant differences between groups will be obtained for both primary outcome measures; however, a positive result on either measure would be clinically meaningful.

There are no data as yet available defining how sensitive any available marker is in predicting a specific survival effect. Some measures have been shown to correlate with survival, but the lack of a study with a clear survival effect during which these measures were obtained makes their relevance still a matter of conjecture. The pivotal studies demonstrating the efficacy of riluzole did not include any other outcome measures in current use. Studies employing functional markers may employ smaller sample sizes and still achieve reasonable power, but their utility is based on as yet untested assumptions about both the relation of such measures to survival and the effect that an effective treatment would be likely to have on these outcomes. We anticipate that by performing a study adequately powered for survival and ALSFRS-R and obtaining multiple secondary outcome measures as well, we will be able to validate these other measures for use in the future [140, 141]. This will be very important in facilitating future trials of different drugs and combinations that might be possible with smaller sample size.

3.2 Study Flow

Figure 5: Study Flow

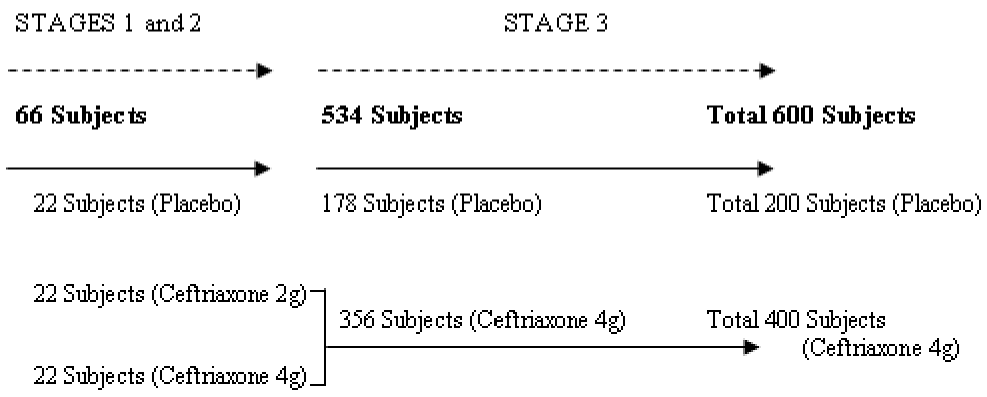

3.3 Setting

The STAGE 1 and 2 studies will be conducted at up to 12 sites, selected because of their excellent record of recruitment, excellent compliance with study protocols and regulations, clinical research expertise and resources. The STAGE 3 study will be expanded to include approximately 70 sites in the United States and Canada.

Each site will employ a site investigator, an alternate physician-investigator who will be available at all times when the site PI is unavailable, a Research Nurse, Nurse Practitioner (NP) or Physician's Assistant (PA), or other qualified health professional, who will perform catheter training and catheter checks, a study coordinator and a clinical evaluator. A nephrology consultant, a gastroenterology consultant, a catheter specialist and an infectious disease specialist consultant will also be required at each site that will be available for clinical consultation for individual subjects.

4. SUBJECT SELECTION AND ENROLLMENT

4.1 Inclusion Criteria

1. Participants with familial or sporadic ALS diagnosed as laboratory supported probable, probable or definite according to the World Federation of Neurology El Escorial criteria [142], Appendix 2
2. Age 18 years or older
3. Capable of providing informed consent and complying with trial procedures.
4. Vital capacity (VC) at least 60% predicted value for gender, height and age at screening
5. Women must not be able to become pregnant (e.g. post menopausal, surgically sterile, or using adequate birth control methods) for the duration of the study. Adequate

contraception includes: abstinence, hormonal contraception (oral contraception, implanted contraception, injected contraception or other hormonal (patch or contraceptive ring, for example) contraception), intrauterine device (IUD) in place for ≥ 3 months, barrier method in conjunction with spermicide, or another adequate method (as determined by steering committee member review). Women of childbearing potential must have a negative pregnancy test at screening and be non-lactating.

6. First ALS symptoms occurred no more than 3 years prior to screening visit
7. Not taking riluzole, or on a stable dosage for at least thirty days prior to the screening visit
8. Subject has a competent caregiver who can and will be responsible for administration of study drug. If there is no caregiver, another qualified individual must be available to administer the study drug.
9. Geographic accessibility to the study site
10. Subjects medically able to undergo placement of central venous catheter as determined by the investigator (to include absence of systemic infection, a medical disorder which precludes catheter placement)

4.2 Exclusion Criteria

1. Dependence on mechanical ventilation (invasive or non-invasive, including Continuous Positive Airway Pressure (CPAP) or Bilevel Positive Airway Pressure (BiPap) for any part of the day or night prior to the screening visit.
2. Exposure to ceftriaxone or any cephalosporin within 30 days prior to the screening visit.
3. History of known sensitivity or intolerability to ceftriaxone or to any other cephalosporin.
4. History of known sensitivity or intolerability to penicillin or any beta lactam (including mild rash).
5. Exposure to any other investigational agent within 30 days prior to screening visit.
6. Known immune compromising illness or therapy
7. Active gastrointestinal disease within 30 days of the screening visit
8. History of antibiotic-induced colitis
9. Active biliary disease, including gallstones
10. Presence of any of the following clinical conditions
    a. Drug abuse or alcoholism
    b. Unstable cardiac, pulmonary, renal, hepatic, endocrine, hematologic, or active infectious disease, including current malignancy
    c. AIDS or AIDS-related complex
    d. Unstable psychiatric illness defined as psychosis or untreated major depression within 90 days of the Screening Visit
11. Laboratory values: Screening alanine aminotransferase (ALT) greater than 3.0 times the upper limit of normal or, total bilirubin greater than 1.5 times the upper limit of normal, absolute neutrophil count of ≤ or 1000/ul, platelet concentration of <100,000/ul, hematocrit level of <33 for female or <35 for male, or coagulation tests ≥ 1.5 times upper limit of normal.
12. Women of childbearing potential not practicing adequate contraception
13. History of known sensitivity to bile acids or ursodiol

Riluzole. The use of riluzole will be permitted during the study. Subjects taking riluzole must be on a stable dosage for 30 days prior to screening. Subjects are not allowed to start taking riluzole during the trial. About 60 percent of patients with ALS in the United States are currently taking riluzole [95, 143]. Allowing subjects the choice of taking riluzole reduces dropout rate and ensures that the investigators know which subjects are taking riluzole.

4.3 Enrollment Procedures

4.3.1 Subject Availability and Recruitment

Each of the up to 12 centers participating in the STAGE 1 and 2 will randomize approximately five to eight subjects. Each site in the STAGE 3 efficacy study will randomize approximately 15 subjects in 24 months. Enrollment will be closed as soon as the 600 subjects have been randomized to treatment assignment. It is anticipated that more than 600 subjects will have to be enrolled to randomize 600 subjects to treatment. The clinical trial will be advertised in local and national ALS newsletters and by letters to local neuromuscular physicians and general neurologists. Based on the increase in number of participating sites and changes in the protocol, we do not anticipate difficulty in recruitment. Each of the participating sites is an active ALS center. Should there still be a lag in accrual of subjects, we would first consider the addition of more sites, followed by lengthening the time for accrual.

4.3.2 Documentation of Screening and Eligibility

Please refer to section *6.2.1 Screening and Informed Consent* for details regarding screening procedures and documentation of ineligibility and nonparticipation of eligible subjects.

4.3.3 Informed Consent

The Coordination Center must receive written confirmation of IRB approval and a copy of the IRB approved consent forms from each site prior to initiation of enrollment at that center. The site investigator or IRB and Sponsor approved designee at the site will explain the protocol and obtain informed consent from all subjects prior to initiation of any research evaluations. The site investigator will determine study eligibility as determined by the inclusion and exclusion criteria. If the study subject agrees, provides informed consent, and signs the IRB approved informed consent form, the study visits are scheduled. Informed consent will also be obtained from the caregiver, by the site investigator or IRB and Sponsor approved designee at the site, with a separate consent form specific to their participation, as they will be asked to fill out a questionnaire periodically throughout the duration of the study. One copy of the signed informed consent form will be given to the subject, and another copy may be maintained in the subject's medical record. The caregiver will receive a copy of the consent they have signed as well. The informed consent details the potential benefits of participating in the research as well as the potential risks of the experimental interventions. Copies of all informed consent forms are found in Appendix 3.

When the final dosage is chosen for the last STAGE of the trial, subjects who entered the trial in STAGE 1 will be asked to sign another consent form or an addendum to the existing consent form. This consent (or addendum) will inform them of the dosage chosen and any changes to the study for this final STAGE of the trial.

4.3.4 Randomization and Randomization Number Assignment

Detailed information on the randomization process is found in the Coordination Center Manual of Operations. The randomization will be stratified by riluzole use and blocked by site in a manner that will allow for a balanced distribution of treated and placebo patients at each site. The randomization scheme will be independently developed by the Biostatistics Center at MGH and will indicate the treatment assignment for each subject (randomization) ID number. For each site, a randomization schedule will be provided to the pharmacy that will indicate the treatment assignment. The Randomization ID will also be used to identify the subject's Source Documents, electronic case report forms (eCRFs), laboratory tests, and all communications. The team of biostatisticians in the Biostatistics Center will develop the randomization plan and randomization sheets to be distributed to each of the research pharmacies under the guidance of the chief biostatistician. All Coordination Center clinical trial staff, with the exception of the Pharmacy Monitor, will remain blinded to all treatment assignments. Subjects, investigators, study monitors, site coordinators, and site clinical evaluators will also be blinded to treatment group assignment throughout the study. For STAGE 1 there will be an additional randomization to the time of the second lumbar puncture for CSF sampling. This randomization will be sent via the electronic data management system to the sites prior to the subject visit.

If a STAGE 1 subject who has been randomized decides to withdraw from the trial within seven days of the randomization date, the subject may be replaced. Subjects enrolled in STAGE 1 who do not successfully complete their pharmacokinetic visit will continue in the study, but will also be replaced as STAGE 1 subjects, so that complete pharmacokinetic data can be obtained on 60 subjects. Successfully completing the pharmacokinetic visit is defined as having CSF collected at the two predefined time points. In this case, the randomization parameters (treatment code, LP time for STAGE 1 subjects) will be returned to the pool of available combinations. The EDC system will be programmed to perform this procedure with manual input from the Data Manager. This replacement will not occur with STAGE 3 subjects.

5. STUDY INTERVENTIONS

5.1 Study Drug – Interventions, Administration, and Duration

5.1.1 Dosage

The treatment arms in STAGES 1 and 2 are ceftriaxone 2 grams per day, ceftriaxone 4 grams (2 g bid) or placebo administered intravenously. All subjects in STAGES 1 and 2 will be instructed to take one syringe (20 ml) in am and one in pm (see Section 5.1.5). The results from the pharmacokinetic study in STAGE 1 and the safety study in STAGE 2 will guide decisions on the final dosage for the STAGE 3 study. The results from the STAGE 1 pharmacokinetic study may also necessitate an adjustment of dosage during STAGE 2. Based on review of unblinded data

by the DSMB and a few members of the Steering Committee, 4 grams/day was chosen as the dosage for STAGE 3. Since the study is an on-going adaptive design study and the current STAGE 1 and 2 subjects are included in the STAGE 3 analyses, no unblinded data will be available until the study is completed.

5.1.2 Study Drug Administration

Ceftriaxone or placebo will be administered via the single lumen catheter using an electronic pump and microbore tubing. Ceftriaxone or placebo (20 ml) is administered intravenously over 20 minutes +/- 10 minutes. The subject will receive the first dose of ceftriaxone at the site on Day 0 and will be instructed on use of pump and how to administer the study drug. Subjects will only take one dose on Day 0. They will start taking the full dosage the following day. All subsequent doses will be administered in the home by the caregiver, with the exception of the doses given at the hospital during study visits.

Prior to receiving the study drug, all subjects and caregivers will participate in training regarding care and use of central venous catheters. Training will include use of the pump, administration of study drug, drug storage and handling, catheter care, and exit site care. The subject and caregiver will be required to pass a competency assessment to demonstrate their knowledge and skills in catheter care and use. This training and competency evaluation will occur prior to catheter placement, so that the possibility of subjects receiving the catheter but not being allowed to start study drug is eliminated. Following discharge, a home visit by a visiting nurse will be available, if needed, to evaluate the exit site, catheter function and the caregiver's ability to care for the catheter and exit site. Throughout the study, trained healthcare personnel will be available to answer questions and offer ongoing instruction as needed. A training video will be provided to each subject for further training at home. Written instructions for storage, thawing, and administration of study drug, as well as for catheter care and prevention of infection, will also be provided to all subjects.

The study drug should be kept frozen and thawed to room temperature just prior to use. Syringes should not be re-frozen. If subjects do not have any adverse reactions with study drug administration, they may be able to administer the study drug over a shorter time period (10 minutes). This decision will be made by the site investigator only after a subject has been on treatment for at least three months.

In STAGEs 1 and 2, the overall rate of biliary events was 26%. This is an expected risk from Ceftriaxone. Symptoms were improved with use of Ursodiol and dosage reduction. Therefore, the study DSMB approved the use of Ursodiol (250 mg po b.i.d.) for subjects in STAGE 3 randomized to ceftriaxone. To maintain study blind, subjects randomized to placebo (MVI) will receive a placebo Ursodiol. Ursodiol and Placebo Ursodiol will be in the form of identical tablets.

5.1.3 Dosing Schedule

The first dose of study drug for each subject will be administered in the clinic and the subject will be observed for at least three hours to assess for any immediate hypersensitivity reaction.

The day that a subject starts treatment with study drug will be designated as DAY 0. All visits must be scheduled from Day 0, not the last assessment. The subjects in STAGE 1 will be randomized (1:1:1) into one of three cohorts; 2 grams ceftriaxone q day, 2 grams ceftriaxone bid, or placebo. Each subject will receive study drug twice a day. The subjects will receive two containers of syringes labeled Container A (or “a.m.”) and Container B (or “p.m.”) filled with study drug. Caregivers will be instructed to administer to the subject the contents of one syringe from Container A in the morning and one from Container B in the evening as follows for STAGE 1:

	**Container**	
**Group**	**A**	**B**
2 grams per day	X	0
4 grams per day	X	X
Placebo	0	0

("0" = Placebo, "X" = ceftriaxone 2 grams)

	**AM**	**PM**
Day 0 – STAGE 2 conclusion	A	B

STAGE 3: The final dosage for STAGE 3 will be 4 g (2 g bid). Study drug will be administered twice daily, once in the morning and once in the evening.

Subjects will receive the study drug until the last enrolled subject completes 52 weeks of treatment.

Subjects will also be instructed to take one tablet (Ursodiol or Ursodiol Placebo) twice daily (AM and PM) with food while on study drug (Ceftriaxone or Placebo).

At a dosage of 4 grams/day, pharmacokinetic study showed that CSF ceftriaxone levels were maintained above our criterion level for at least 60 hours. Therefore, subjects will be allowed to take “drug holidays” of up to 2 days per week on an as needed basis. Participants who are tolerating a daily regimen well will not be encouraged to take drug holidays. However, for those that find the dosing regimen arduous, this option may help with subject retention. The overall requirement will be that participants receive at least five days of drug dosing every seven days, and that at least 4 days must elapse between drug holidays. Either one or two day holidays will be permitted. The numbers of dosages used between visits will be tracked. The relationship of total dosage to outcome will be analyzed as a secondary analysis.

5.2 Handling of Study Interventions

5.2.1 Study Drug Distribution to Sites

Ceftriaxone, Pediatric Multivitamin Solution, Ursodiol and Ursodiol Placebo will be distributed by the MGH central research pharmacy to all US sites. A Canadian central research pharmacy will be used for distribution to all Canadian sites. Both the MGH central pharmacy and the Canadian central research pharmacy will keep detailed documentation of the amount and dates that study drug is shipped to the clinical sites. The individual site pharmacies will keep a log of dispensed drug/placebo identified by subject randomization ID, subject initials and date dispensed. Ceftriaxone will be provided to site pharmacies as a sterile crystalline powder for reconstitution with sterile water. The site research pharmacists will be unblinded to treatment assignment and will make the placebo and ceftriaxone solutions. Ceftriaxone is a commonly made solution and site pharmacists are familiar with how to reconstitute. Written instructions will be provided to all site research pharmacists on study drug preparation and dispensing. The Study Monitors are blinded, so a separate Pharmacy Monitor will meet with the pharmacists to ensure that study procedures are followed. Ceftriaxone can be dissolved and administered in sterile water, 5 % dextrose or normal saline. It is stable frozen for up to 180 days when reconstituted with sterile water. For this reason, we elected to use sterile water to reconstitute ceftriaxone [144].

Ceftriaxone is a white to yellowish-orange crystalline powder. The color of ceftriaxone in solution is light yellow. The placebo will consist of standard pediatric multivitamin (MVI) formulation mixed with 20 cc of sterile normal saline (Appendix 1). The placebo concentration will be 0.20 ml +/- 0.05 ml of pediatric MVI in 20cc of normal saline. This range exists in order to adjust the color of the placebo, particularly from different lot numbers, for blinding. The change in concentation will not affect the safety of the placebo. The concentration of pediatric MVI was chosen to match the color of ceftriaxone in solution.

5.2.2 Study Drug Dispensing, Labeling and Storage

The site research pharmacists will be unblinded to treatment assignment. Each site research pharmacist will check the condition of the study drug upon receipt and enter this data into the Proof of Receipt letter (packing slip) and accountability record. All study drug dispensed by the investigator will be accounted for throughout the study and drug will be maintained in a secure area at each site. Ceftriaxone sterile powder should be stored at room temperature protected from light. After reconstitution, protection from normal light is not necessary. Study drug syringes as well as bottles of Ursodiol and Ursodiol Placebo will be labeled in a double blind manner without reference to content of active drug or placebo. Information presented on the labels for all study drug will comply with FDA or Health Canada requirements, as appropriate. Study drug will be dispensed at the Baseline visit and every four weeks following the Baseline visit. Supply of study drug shall be dispensed for administration of a sufficient number of doses to allow for some delay between scheduled and actual follow up visits. Syringes with frozen study drug and bottles of Ursodiol (or placebo Ursodiol)tablets will be dispensed to each participant with instructions to return all unused syringes of study drug and empty syringes every four weeks following the Baseline visit. At each visit, each subject will be provided with

syringes of study drug, a supply of Ursodiol, catheter care supplies, and suitable container for disposal of used syringes. An instruction manual with details on procedures for storage, thawing, and administration of study drug and recording of dosing and adverse events will be given to each subject for reference.

5.2.3 Compliance and Return of Study Drug (Adherence Assessment)

The primary method to check compliance for study drug will be to record the number of syringes dispensed and the number of unused syringes returned at each visit in the eCRF. Similarly, the number of Ursodiol (or placebo) tablets dispensed and returned will be counted at each visit and recorded in the eCRF. Subjects will be instructed to return all unused study drug every four weeks following the Baseline visit. Subjects will be instructed to keep a drug log (Subject Diary) to return at each visit. Historically, subjects with ALS have a very high compliance rate (over 95%) with medications. During each visit, a site staff member, known as the "Independent Drug Counter," will count and record the number of unused (full) syringes returned. The Independent Drug Counter will dispose of the full syringes along with the empties in the container. The number of syringes will be monitored, and compared to expected numbers, to determine adherence to the protocol. To maintain study blind, neither the site coordinator nor clinical evaluator can serve as the Independent Drug Counter.

Study drug will be distributed as needed for the next four-week interval. In addition, blood samples will be obtained at week four and every 16 weeks thereafter to determine plasma concentrations of ceftriaxone. Blood samples for plasma concentrations will be drawn for all subjects. The results will not be available to the Coordination Center or site staff until study completion. These determinations will enhance our monitoring of compliance as ceftriaxone is available by prescription and we will need to assess for potential unassigned use of ceftriaxone in subjects assigned to the placebo arm.

5.3 Concomitant Medications

Throughout the study, investigators may prescribe any other concomitant medications or treatments deemed necessary to provide adequate supportive care providing that they are licensed in the US or Canada, as applicable. All concomitant medications received by a subject will be recorded on the appropriate source documents and in the Electronic Data Capture (EDC) System.

5.3.1 Exclusionary Medications

Subjects may not take open-label ceftriaxone, other cephalosporins, or beta lactam antibiotics or other experimental agents during the study. Other medications that are prohibited during the course of the study include cyclosporins, certain chemotherapy therapeutics, and medications currently in use as experimental treatments for ALS. A list of exclusionary and precautionary medications will be included on the web portal and may be given as a handout to subjects. If necessary to treat a clinical condition, and if a suitable alternative does not exist, beta lactams and/or cephalosporins may be prescribed for short-term use.

Medications to use with caution include IV aminoglycosides, as concomitant use with cephalosporins may increase the risk of nephrotoxicity. The dosage of Warfarin may need to be altered in subjects receiving ceftriaxone. Subjects on anticoagulation therapy will need to have their blood tests (PT/INR) checked on a regular basis, as determined by the physician prescribing warfarin. Subjects will be instructed to use the CVC only for administration of study drug and will not be permitted to utilize the catheter for other purposes.

5.4 Dosage Changes

Instructions for dosage changes are contained in the Manual of Operations (MOP). These instructions may be modified as indicated throughout the course of the study. As instructions are updated, the MOP will be modified accordingly.

5.4.1 Dosage Reduction

The investigator may temporarily reduce or stop the study drug for adverse events thought related to study drug. If the adverse event is mild or moderate, the dosage may be reduced by half, until the event improves, or the dosage may be further reduced as needed. Subjects may remain on the reduced dosage until the condition improves. The site investigator may then choose to resume the higher dosage or maintain the subject at the reduced dosage. If the event is serious or life threatening, the study drug should be suspended immediately. The site will contact the Coordination Center within 24 hours of any dosage reduction.

5.4.2 Dosage Suspension

If an adverse event is serious or life threatening, the study drug should be suspended. Additionally, a "drug suspension" may occur at the Site Investigator's discretion. The site will notify the Coordination Center within 24 hours of any dosage suspension.

5.4.3 Dosage Re-challenge

The Site Investigator may choose to re-challenge a subject if the adverse event resolves. If the study drug was suspended for another reason (such as travel), the study drug can be re-started at the discretion of the site investigator. The site will notify the Coordination Center prior to re-challenging a subject.

5.4.4 Dosage Discontinuation

Certain conditions require that study drug be permanently discontinued for safety reasons. Additionally, a subject may choose to permanently discontinue study drug but still be followed for outcome measures. The site will notify the Coordination Center within 24 hours of learning about a serious adverse event and/or study drug discontinuation.

5.5 Code Break Procedures

An emergency unblinding procedure will allow site investigators the option of disclosing the treatment assignment for an individual subject if clinical circumstances require such an unblinding. The site pharmacists will have randomization numbers and treatment assignments and can release this information to the site investigators if unblinding becomes necessary. Rarely is such an extreme action taken. Experimental medications can usually be suspended in a subject experiencing adverse effects without the need for unblinding. In the event that emergency disclosure of treatment assignment is required, the site investigator should contact the Coordination Center prior to unblinding. The site pharmacist will also notify the Coordination Center of any emergency unblinding within 24 hours of occurrence.

6. CLINICAL AND LABORATORY EVALUATIONS

6.1 Schedule of Assessments

The Schedule of Assessments outlines all study procedures (Figure 6).

Procedures highlighted in dark gray were performed in Stages 1 and 2 but will not be performed during Stage 3.

Week Number	Screening[1]	Catheter placement	Pre-Random. CVC Check	Baseline (Day 0)	1	2	4	8	12	16	20	24	28	32	36	40	44
**EVALUATIONS***																	
Informed Consent	X																
Inclusion/Exclusion Criteria	X	X															
Screening Visit Registration	X																
Medical History	X																
Complete Physical Exam	X									X							
Central Venous Catheter Placement		X															
Chest X-ray		X															
Randomization (Assignment of subject #)				X													
Pharmacokinetic Study[2] (plasma & CSF)					X												
Vital Signs	X	X	X o r status	X	X	X	X	X	X	X	X	X	X	X	X	X	X
Weight	X			X			X			X				X			
Central Venous Catheter Check[1]			Site or phone	X	X	X	X	X	X	X	X	X	X	X	X	X	X
Vital Capacity	X						X			X				X			
Muscle Strength Testing	X						X			X				X			
ALSFRS-R	X						X			X		X		X		X	
ALSSQOL Questionnaire	X						X			X				X			
Caregiver Burden Inventory	X						X			X				X			
Concomitant Medications	X	X		X	X	X	X	X	X	X	X	X	X	X	X	X	X
Adverse Events		X	X	X	X	X	X	X	X	X	X	X	X	X	X	X	X
Safety Labs (blood draw)[2]	X				X	X	X	X	X	X	X	X	X	X	X	X	X
Ceftriaxone Plasma Level (blood draw)							X			X				X			
Blood draw for Biomarkers												X					
Urinalysis/Spot Urine for protein and creatinine[3]	X				X	X	X	X	X	X	X	X	X	X	X	X	X
Dispense Study Drug				X			X	X	X	X	X	X	X	X	X	X	X
Drug Accountability/Compliance							X	X	X	X	X	X	X	X	X	X	X
Abdominal Ultrasound	X						X	X	X			X			X		
Abdominal Ultrasound	X						X	X			X						
Pregnancy Test (Serum)	X	As needed for duration of trial															

*** Procedures highlighted in dark gray were performed in Stages 1 and 2 but will not be performed during Stage 3.**

1. Central Venous Catheter may be checked in between visits by home infusion company as needed
2. Safety labs will be sent to a central laboratory and will include complete blood count and differential, serum electrolytes, BUN, Creatinine, ALT, AST, serum glucose and total Bilirubin. PT, PTT and platelets are only performed at screening visit, unless otherwise indicated (at discretion of site Investigator)
3. 24-hour urine collection was also performed in Stage 1 (Screening and every 12 weeks), but will not be performed for Stage 3

Week Number	48	52	56	60	64	68	72	76	80	84	88	92	96
**EVALUATIONS**													
Informed Consent													
Inclusion/Exclusion Criteria													
Screening Visit Registration													
Medical History													
Complete Physical Exam		X											
Central Venous Catheter Placement													
Chest X-ray													
Randomization (Assignment of subject #)													
Pharmacokinetic Study[2](plasma & CSF)													
Vital Signs	X	X	X	X	X	X	X	X	X	X	X	X	X
Weight	X				X				X				X
Central Venous Catheter Check[1]	X	X	X	X	X	X	X	X	X	X	X	X	X
Vital Capacity	X				X				X				X
Muscle Strength Testing	X				X				X				X
ALSFRS-R	X		X		X		X		X		X		X
ALSSQOL Questionnaire	X				X				X				X
Caregiver Burden Inventory	X				X				X				X
Concomitant Medications	X	X	X	X	X	X	X	X	X	X	X	X	X
Adverse Events	X	X	X	X	X	X	X	X	X	X	X	X	X
Safety Labs (blood draw)[2]	X	X	X	X	X	X	X	X	X	X	X	X	X
Ceftriaxone Plasma Level (blood draw)	X				X				X				X
Blood draw for Biomarkers		X											
Urinalysis/Spot Urine for protein and creatinine[3]	X	X	X	X	X	X	X	X	X	X	X	X	X
Dispense Study Drug	X	X	X	X	X	X	X	X	X	X	X	X	X
Drug Accountability/Compliance	X	X	X	X	X	X	X	X	X	X	X	X	X
Abdominal Ultrasound	X			X			X			X			X
Abdominal Ultrasound													
Pregnancy Test (Serum)	As needed for duration of trial												

Week Number	100	104	108	112	116	120	124	128	132	136	140	144	148
**EVALUATIONS**													
Informed Consent													
Inclusion/Exclusion Criteria													
Screening Visit Registration													
Medical History													
Complete Physical Exam													
Central Venous Catheter Placement													
Chest X-ray													
Randomization (Assignment of subject #)													
Pharmacokinetic Study[2](plasma & CSF)													
Vital Signs	X	X	X	X	X	X	X	X	X	X	X	X	X
Weight				X				X				X	
Central Venous Catheter Check[1]	X	X	X	X	X	X	X	X	X	X	X	X	X
Vital Capacity				X				X				X	
Muscle Strength Testing				X				X				X	
ALSFRS-R		X		X		X		X		X		X	
ALSSQOL Questionnaire				X				X				X	
Caregiver Burden Inventory				X				X				X	
Concomitant Medications	X	X	X	X	X	X	X	X	X	X	X	X	X
Adverse Events	X	X	X	X	X	X	X	X	X	X	X	X	X
Safety Labs (blood draw)[2]	X	X	X	X	X	X	X	X	X	X	X	X	X
Ceftriaxone Plasma Level (blood draw)				X				X				X	
Blood draw for Biomarkers													
Urinalysis/Spot Urine for protein and creatinine[3]	X	X	X	X	X	X	X	X	X	X	X	X	X
Dispense Study Drug	X	X	X	X	X	X	X	X	X	X	X	X	X
Drug Accountability/Compliance	X	X	X	X	X	X	X	X	X	X	X	X	X
Abdominal Ultrasound			X			X			X			X	
Abdominal Ultrasound													
Pregnancy Test (Serum)	As needed												

Week Number	152	156	FINAL STUDY VISIT[4]	30-day Follow-up Phone Call
**EVALUATIONS**				
Informed Consent				
Inclusion/Exclusion Criteria				
Screening Visit Registration				
Medical History				
Complete Physical Exam			X	
Central Venous Catheter Placement				
Chest X-ray				
Randomization (Assignment of subject #)				
Pharmacokinetic Study[2](plasma & CSF)				
Vital Signs	X	X	X	Vital Status
Weight			X	
Central Venous Catheter Check[1]	X	X	X	
Vital Capacity			X	
Muscle Strength Testing			X	
ALSFRS-R	X		X	
ALSSQOL Questionnaire			X	
Caregiver Burden Inventory			X	
Concomitant Medications	X	X	X	
Adverse Events	X	X	X	X
Safety Labs (blood draw)[2]	X	X	X	
Ceftriaxone Plasma Level (blood draw)				
Blood draw for Biomarkers				
Urinalysis/Spot Urine for protein and creatinine[3]	X	X	X	
Dispense Study Drug	X	X		
Drug Accountability/Compliance	X	X	X	
Abdominal Ultrasound		X	X	
Abdominal Ultrasound				
Pregnancy Test (Serum)	As needed			

4. Final Study Visit: performed when subject completes trial

6.2.1 Timing of Evaluations

The Coordination Center will require a copy of each site's written Institutional Review Board (IRB) approval of the protocol and approved consent form prior to site initiation. Subjects enroll in the study either at STAGE 1, or at STAGE 3 when the study expands to an efficacy study. The study procedures are identical for subjects enrolling in STAGE 1 as those enrolling in STAGE 3 with the following two exceptions:

1. Only subjects enrolling in STAGE 1 will undergo the pharmacokinetic study at Week one (Day 7).
2. Subjects entering study at STAGE 1 are randomized to one of three treatment arms (placebo, 2 grams/day or 4 grams/day of ceftriaxone), while subjects entering the study at STAGE 3 are randomized into one of two arms (placebo or ceftriaxone; 1 to 2 randomization). The final study dosage of ceftriaxone for STAGE 3 for all subjects will be 4 g (2 g bid) per day.

6.2.1 Screening and Informed Consent

At the screening visit, potential subjects will be informed about study procedures and will then sign an informed consent form (Appendix 3). Screening procedures will take place within 28 days of the baseline (randomization) visit. The inclusion/exclusion criteria will be reviewed and a medical history and full physical examination completed. The VC will be determined. Vital signs, weight and concomitant medications will be recorded. Safety laboratory tests will be performed including complete blood count (CBC) with differential, prothrombin time (PT), partial thromboplastin time (PTT), platelets, electrolytes (including phosphorous and calcium), BUN, creatinine, ALT, AST, total bilirubin, albumin, urinalysis including microanalysis (with culture and sensitivity when WBCs are present), and a pregnancy test for women of child-bearing potential. Pregnancy tests will be repeated as necessary throughout the trial.

An abdominal ultrasound will be performed at the screening visit to monitor for biliary disease. Hand held dynamometry for muscle strength will be performed, as well as the ALSFRS-R and Quality of Life questionnaires. The outcome measures performed at this visit will be the pre-treatment measures.

As part of the screening visit, the site investigator will determine the competence of caregiver to administer the study drug at home. Teaching will begin on the method of study drug administration and catheter care. All inclusion and exclusion criteria and safety laboratory tests will be reviewed by the site investigator prior to scheduling the catheter placement visit. If the subject passes all screening procedures and is confirmed as eligible, the catheter placement visit can be scheduled. The site study staff will electronically register the subject to the study and the subject will be assigned a screening identification number. Only registered subjects can go on to have catheter placement and randomization. This process will ensure that only subjects who are eligible for the study will proceed to have placement of a central venous catheter. A log will be kept at the site to record all subjects screened for entry into the study. This information will also be captured electronically. Demographic characteristics of all subjects who are screened will be recorded whether or not they qualify for entry into the study. The reason for non-qualification

will be recorded for all subjects who are not eligible. The reason for non-participation will also be recorded for subjects who are eligible but choose not to participate in the trial. If a subject fails screening for the study, the subject can be re-screened if the Site Investigator determines it is appropriate to do so. At any time during the study, repeat laboratory tests can be obtained if the Site Investigator or central laboratory thinks that a laboratory test result is in error. Additional laboratory tests, including coagulation tests, may also be obtained at any time during the study, at the discretion of the Site Investigator.

Between screening and the catheter placement visit, the subject and caregiver may return to the site for teaching visits. At these visits, the study Nurse, Physician Assistant or Physician will teach use and care of central venous catheters. The caregiver will need to pass a competency test prior to catheter placement. As many visits for training as are needed will be permitted.

6.2.2 On-Study Evaluations

Central Venous Catheter Placement Visit. The catheter for venous access will be placed within 26 days of the screening visit. The entry criteria will be reviewed again and the subjects will have vital signs checked. A single-lumen tunneled Hickman catheter will be placed in the outpatient setting by a trained operator in the Interventional Radiology lab or other appropriate setting within the institution. Placement will follow institutional guidelines at each site, which may include placement under fluoroscopy and/or the administration of an antibiotic as part of the procedure. Prior to study initiation, all sites will provide written verification of experience with percutaneous catheter placements. Full details of catheter implantation and subsequent use and care will be provided to the sites in the Manual of Operations. Each subject will have a radiographic evaluation (such as x-rays or fluoroscopy) following catheter placement to assess for pneumothorax and for evaluation of placement of the tip of catheter.

If possible, the baseline (day 0) visit and randomization will occur on the same day as the catheter placement. At this time, the study drug will be dispensed and the first dose will be administered. Due to differences in site organization, some subjects may not be able to be randomized and provided with study drug at the catheter placement visit. These subjects will be randomized and receive their study drug (Baseline visit, day 0) no more than 28 days after the screening visit. These subjects will flush the catheter daily with normal saline until randomization occurs. Every effort should be made to randomize and have subjects begin study drug as soon as possible following catheter placement.

Following screening and prior to the placement of the catheter, subjects and their families/caregiver will be taught maintenance and care of the central venous catheter, warning signs and symptoms, storage of study drug, aseptic technique, infusion technique, and exit site and dressing care. Additionally, the subject and/or caregiver will demonstrate their proficiency and pass a competency assessment. This instruction will occur in the outpatient and/or home setting. Upon discharge, following catheter placement, the subject may have their exit site and catheter evaluated at home by a visiting nurse for one initial visit, if this is felt to be necessary by the site investigator. The subjects' and caregivers' understanding and technique will be further evaluated during site visits, and the educational process will be reinforced. Standards of the Visiting Nurses of America regarding catheter care will serve as guidelines for home care nurses.

Hickman catheter care including sterile technique, dressing changes, signs/symptoms of infection, clamping, cap changes and administration of study drug will be reviewed during home and site visits. This will be performed by the site research nurse/PA (or other qualified medical professional) during each site visit. The homecare service will remain available throughout the study for problems and catheter checks as needed. Clinical study staff will be available on 24-hour call for early intervention regarding possible problems and to be available to answer subject/family questions.

At catheter placement visit and all subsequent visits, subjects will be provided with supplies for flushing the catheter, catheter care and dressing changes. Subjects will also receive written and DVD instructions on catheter care.

Pre –Randomization Central Venous Catheter Check. Prior to randomization, the catheter and the exit site for the catheter will be evaluated to ensure the catheter is functioning properly and that there are no signs of fever or significant catheter complications, such as infection. Subjects will not be randomized if there is evidence of fever or any significant catheter complications. This visit will occur only if the Catheter Placement and Baseline Visits do not occur on the same day, and may occur in person in the clinic or at a home visit.

Baseline and Randomization (DAY 0). The baseline visit (Day 0), including randomization, study drug dispensing, and the first dose of study drug, may occur on the same day as the catheter placement visit. If this is not possible, subjects will return for a baseline visit within 28 days of the screening visit. The catheter and exit site will be evaluated by a qualified medical provider. Vital signs and weight will be checked. Subjects will not be randomized if there is evidence of fever or any significant catheter complications.

STAGE 1: At the time that eligibility into the study is established, each subject will be randomly assigned to ceftriaxone 2 grams per day, ceftriaxone 2 grams twice a day, or placebo using a 1:1:1 allocation.

STAGE 3: Eligible subjects entering the study at STAGE 3 (efficacy) will be randomized to one of two treatment arms, ceftriaxone 4 g (2 g bid) per day or placebo using a 2:1 active: placebo allocation.

Pharmacokinetic Study (STAGE 1 only, Week 1 Visit). The study will provide information on steady state levels and population pharmacokinetics of ceftriaxone in the CSF in subjects with ALS and address whether CSF penetration is dose dependent. The results will be used to determine whether adequate CSF levels can be achieved in subjects with ALS without inflamed meninges, and determine the frequency of administration of ceftriaxone to maintain a biologically meaningful CSF levels. We will target the trough level rather than the mean, since the actual level will always be greater than or equal to the trough. The target level is based on *in vitro* data that suggests that CSF level greater than 1 μM (0.5525 μg/ml) might be a reasonable CSF level. Ceftriaxone levels will be determined in plasma and CSF from 60 randomized subjects with ALS.

After seven days of treatment all subjects from STAGE 1 will be admitted in the morning to the hospital for a 12-hour pharmacokinetic study. The visit cannot be performed sooner than day seven. If it is not possible to perform the visit by day eight, the site will contact the Coordination Center immediately. Subjects may eat a light breakfast prior to administration of the infusion and may then follow a house diet. No other restrictions are needed. Subjects will not self-administer their morning dose. This dose will be given to them as the infusion for the pharmacokinetic study. The Coordination Center will provide sample collection tubes, cryovials, labels, and instructions for sample collections.

Predose. Vital signs and safety laboratory samples will be obtained prior to study drug infusion. Cerebrospinal fluid (approximately 3 ccs) and plasma trough levels will be collected for determination of ceftriaxone levels prior to administration of study drug. Protein concentration and white blood count in the CSF will be determined.

At this time, subjects will be given the option for collection of an additional 5 ccs of CSF for repository in a tissue bank at MGH. This additional CSF will be obtained during the predose lumbar puncture, and will be sent to the MGH repository. The sample will be used for future studies in ALS.

Infusion. Immediately after the trough samples have been drawn, the subject's assigned study drug (20 ml volume) will be administered by intravenous infusion over 20 $\pm$ 10 minutes. Plasma samples will be collected for ceftriaxone levels from a heparin lock inserted into the arm at the following times after the start of the infusion: 0.5, 1, 2, 3, 4, 6, 8, 10 and 12 hours. The blood will be collected in a pre-heparinized tube. The plasma will be separated by centrifugation at 2500 rpm for 10 minutes. Immediately after centrifugation, the plasma will be aliquoted into polypropylene screw cap tubes and immediately frozen at –80ºC until analysis.

In each cohort, all subjects will have a second CSF sample obtained by lumbar puncture at one of the following time points: 2, 4, 6, 8, or 10 hours post infusion. At each of the five time points, CSF will be obtained by lumbar puncture from 4 subjects per cohort. Cerebrospinal fluid samples will be centrifuged at 1000 rpm for 5 minutes, aliquoted, and stored frozen at -80ºC. These levels will be used to model the CSF population kinetics. The subjects can then receive their evening dose in the hospital and be discharged home. The following day the subjects will continue on their treatment schedule. Trough blood samples will be drawn for ceftriaxone levels after 4 weeks of treatment, after 16 weeks of treatment, and then every 16 weeks until study completion. The sites will record the date and time of last study drug administration and the date and time of each sample collection.

Subjects who enter the study in STAGE 3 will return to the site for the Week 1 Visit, but will not complete the pharmacokinetic (CSF and blood for plasma levels) portion of that visit. For STAGE 3 subjects, the Week 1 visit will be a safety visit only.

Measurement of Ceftriaxone Concentrations. A number of HPLC-based assay methods have been published for detection of ceftriaxone in plasma and CSF [84, 145-147]. The procedures utilize an isocratic system with UV detection for analysis of deproteinized samples. Trained personnel who will remain blinded to treatment assignment will perform all measurements. The

validity of the HPLC assay will be determined prior to running study samples. Ceftriaxone is obtained from the pharmaceutical manufacturer (Hoffmann-LaRoche, Nutley, NJ) and the internal standard lidocaine is commercially available (Sigma; St. Louis, MO). Acetonitrile and methanol (Burdick and Jackson; Muskegon, MI) are HPLC reagent grade. Monobasic potassium phosphate (Sigma), dibasic sodium phosphate (Sigma) and cetyltrimethylammonium bromide (CTAB) (Fisher, Fairlawn, NJ) are analytical reagent grade. The HPLC system consists of a pump (Beckman; Berkeley, CA), a Wisp autosampler (Waters), a variable wavelength ultraviolet detector (Waters) set at 270 nm, and a reversed-phase C_{18} column (Waters). CTAB is used an ion-pairing agent. The mobile phase consist of methanol-acetonitrile-phosphate buffer, pH7.4 (30:40:30, v/v/v). Samples are prepared by addition of acetonitrile followed by vortexing and centrifugation. The supernatant is collected and injected. Millennium software (Waters) is used to acquire and handle all spectra data and chromatograms.

Because the plasma range may exceed the expected boundaries of 1-300 mg/liter, reinjections of outlying samples with standard curves verified over the extended region may be necessary. Similarly, the CSF range is expected to be approximately 0.1-10.0 mg/liter, and samples outside the range will need to be reanalyzed with an appropriate standard curve. The estimation of pharmacokinetic parameters is described in the data analysis section (Section 9.5, below). The measurements for the STAGE 1 study will be performed when all 60 subjects complete the pharmacokinetic study. All ceftriaxone level results will be kept at Tufts until time for analysis; at which time they will be provided to the MGH Biostatistics Center for reports to the NINDS Data Safety Monitoring Board (DSMB).

Study Visits (Figure 6). After randomization, all subjects will return once a week for the first two weeks, then at week 4. From this point forward, subjects will be seen every 4 weeks until study completion. The visit window is plus or minus 3 days, except for the day 7 visit (+1) for STAGE 1 participants, calculated from the Baseline visit (Day 0). At every study visit participants will be seen for assessment of vital signs, adverse events, and concomitant medications. Urine will be collected and safety laboratory studies will be drawn at screening, weeks 1, 2, 4, and at every study visit following week 4. Study drug accountability and compliance will be assessed at every study visit. The ALSFRS-R will be completed at the screening visit, Week 4 and every 8 weeks starting at week 16. Subjects will have an abdominal ultrasound performed at weeks 4, 8, and 20 to monitor for biliary disease. If there is evidence of biliary disease, the site will repeat the ultrasound as clinically necessary until this condition has resolved. Muscle strength testing, VC, quality of life questionnaires, weight and plasma samples for trough ceftriaxone levels will be obtained every 16 weeks for study duration.

Blood for ceftriaxone levels will be drawn in the morning, prior to first dose. The subjects may receive their first dose at the clinic. This will serve as another opportunity to monitor how the caregiver administers the study drug. At each visit, subjects will be monitored for problems with self-administration of study drug as well as for evidence of drug reaction or skin infections at the central venous catheter site.

Missed Visits. When a subject fails to appear for a scheduled visit, the site should contact the subject and a partial evaluation may be performed over the telephone, or if possible by a visit to the subject's home. If a subject is evaluated by telephone, the following information should be

obtained: vital status, adverse events, ALSFRS-R, review of ventilator status and mechanical ventilation record and study drug compliance check. The site should stress that safety labs are critical, and should strongly encourage the subject to report to a clinical laboratory designated by the central clinical laboratory to have safety laboratory studies performed. If either a telephone contact or a home visit is made, an additional supply of study drug may be dispensed. The subject should be strongly encouraged to appear for the next scheduled evaluation. Site staff are encouraged to be willing to travel to the participant if needed.

6.2.3 Intervention Discontinuation Evaluations

In clinical trials in ALS, the primary reasons for stopping intervention early include burden to travel to the site, caregiver burden, disease progression, and adverse events were the reasons. The following are measures to optimize study participation retention:

- Increase the number of sites to reduce the travel burden for study subjects
- Provide reimbursement for subjects to travel to sites for visits.
- Provide reimbursement for study staff to travel to subjects' homes for visits.
- Train site investigators and coordinators to recruit subjects who live within a reasonable distance to the site, and to be willing to travel to the subject if needed.
- Train site investigators at the orientation meeting and quarterly on the adverse event management plan. This is to ensure that all site staff are familiar with the potential adverse events and how to manage them to minimize subject discontinuation from the study.

Treatment Withdrawals and Loss to Follow-up. A subject has the right to refuse study treatment and study visits at any time and for any reason. A subject can also be withdrawn from treatment for intolerable adverse events. All efforts should be made to follow the subject for resolution of the adverse event and for survival. The site investigator must notify the Coordination Center within 24 hours of any subject permanently discontinuing study drug, documenting the reasons for discontinuation in the EDC and on the appropriate source documents.

If a subject discontinues study drug at any time, outcome measures will be performed and documented at the study visits following discontinuation of study drug. Follow-up of subjects who have discontinued study drug will continue in the intention to treat analysis. All attempts will be made to follow these subjects for all outcome measures. These assessments include ALSFRS-R and vital status every 8 weeks. In addition, every effort will be made to encourage subjects to return to the study center every 16 weeks for the full battery of outcome measures. The analysis of data from subjects who stopped treatment and/or refused study visits is discussed in the data analysis section.

The subject can agree or decline to return for these study visits at any time during the course of the trial. However, given the intent to treat primary analysis, the importance of participating in outcomes measure assessments even after drug discontinuation will be stressed. In all circumstances, whether or not the subject agrees to continue 16-week visits, the subject will be asked for permission for them to be contacted every 8 weeks via telephone and 12 months after

their Baseline (Day 0) visit and at the end of the study, for follow up on ALSFRS-R and survival status. At the very least, the time that each subject died or began PAV will be determined during this phone call, for the intent to treat analysis of survival. Within the limits of consent and participation, every effort will be made to collect survival and ALSFRS-R data on subjects.

Replacement of Subjects. If a STAGE 1 subject withdraws from the trial at any point prior to the day 7 (week 1) visit and Pharmacokinetic study, that subject will be replaced in order to ensure adequate data for pharmacokinetic and safety analysis for the STAGE 1 portion of the trial. Subjects enrolled in STAGE 1 who do not successfully complete their pharmacokinetic visit will be replaced so that complete pharmacokinetic data can be obtained on 60 subjects. Successfully completing the pharmacokinetic visit is defined as having CSF collected at the two predefined time points. Having complete pharmacokinetic data is important for the decisions on dosage and whether to go forward to STAGE 3 of the study. The replaced subjects whose CSF could not be obtained will continue in the study on treatment. There will be no replacement of subjects in STAGE 3.

6.2.4 Final Evaluations and Post Intervention Phone Call

Once the estimated date of final dose for the last enrolled subjects is known, each site will contact their subjects to schedule the final study visit. Study drug will be discontinued at the final study visit; the CVC will be removed. The site staff will contact the subject or caregiver 30 days following the final visit. The purpose of this telephone call will be to ascertain the subject's medical status, including pregnancy status, to determine whether any adverse events that were ongoing at the last clinic visit have resolved, and to collect any other adverse events occurring since the last visit.

6.3 Special Instructions and Definitions of Evaluations

6.3.1 Informed Consent

The informed consent process is described in Sections 4.3.3 and section 6.6.1.

6.3.2 Documentation of ALS

To be eligible for participation in this trial, participants must have familial or sporadic ALS diagnosed as laboratory supported probable, probable or definite according to the World Federation of Neurology El Escorial criteria [142]. Each Site Investigator will be responsible for documenting this diagnosis, based on the specific El Escorial criteria. This criteria will be clearly designated on source documentation and in the electronic case report forms.

6.3.3 Protocol Violations.

Missed visits and any procedures not performed (not attempted) for reasons other than illness or progressive disability will be reported as protocol violations. Procedures or visits not performed due to illness or disability, procedures that were attempted but failed will not be reported as protocol violations. Study drug compliance that is outside the limits set in the Manual of

Operations will be reported as a protocol violation. Details and specific instructions regarding protocol violations, including any exceptions to this standard procedure, are found in the *Protocol Compliance* section of the Manual of Operations.

6.3.4 Clinical Outcome Measures

STAGE 1

The primary outcome measure for STAGE 1 is the trough steady state ceftriaxone level in the CSF. Our criterion for an effective dosage is a dosage at which at least 80% of the subjects achieve a trough level of 1μM. The secondary outcome measures are the population pharmacokinetics of ceftriaxone in CSF and plasma and the relationship between CSF and plasma levels. Since obtaining CSF levels require a procedure with a side effect risk, we will only obtain CSF levels before dosing and then one additional level that will vary by individual. The second level will be different for different subjects at 2, 4, 6, 8, or 10 hours after the dose based on an optimal sampling plan to be determined by simulations. With these data we will be able to estimate the population pharmacokinetics and determine the effect of other dose regimens. Possible covariates in the population pharmacokinetic model will include riluzole use, weight and height. It is possible that the study could ultimately allow plasma level to serve as a predictive surrogate for CSF level.

STAGE 2

The primary outcome measure for STAGE 2 is the subject's ability to continue taking the study drug without significant side effects. Of particular interest would be side effects that would not be tolerable in a drug that might have to be administered life long. Given the severity of the disease fairly severe side effects might be tolerated. The side effect profile combined with pharmacokinetics will be used to determine the dosage of the drug used in STAGE 3.

STAGE 3

Primary Outcome measures. Co-primary outcome measures include survival, defined as time to death, tracheostomy or the initiation of permanent assisted ventilation (PAV) and change in function as measured by ALSFRS-R. Non-invasive ventilation techniques utilized for more than 22 hours daily for more than one week will be the criterion for determining PAV. The primary analysis will be by "intention to treat." We will ascertain the status of all subjects at the end of the study and every subject will be included in the analysis whether or not they elected to stop treatment before the end of the study. The analysis of survival will use a log-rank test stratified by riluzole use. The use of the log-rank test and a design where everyone is treated until the end of the study has several advantages over treating everyone for only 52 weeks and using the 52-week mortality. By enrolling subjects at a constant rate in over an approximate 24-month enrollment period, we will be able to follow subjects anywhere from 12-36 months, with an average of 18 months of on-treatment follow-up. This is close to estimated median survival time found in previous ALS studies [95]. The power of tests comparing survival distributions depends on the number of events during the trial. Thus there are substantial power advantages

for treating people for a longer duration. To examine the co-primary endpoints jointly, our primary analysis strategy is to use the "shared parameter" model described in Vonesh, Greene and Schluchter[148]. This is described in more detail below in Section 9.1.3.The data analysis will be based on the ALSFRS-R during the first 12 months of treatment.

ALSFRS-R. The ALSFRS-R is a quickly administered (five minute) ordinal rating scale (ratings 0-4) used to determine patients' assessment of their capability and independence in 12 functional activities/questions. All 12 activities are relevant in ALS. Initial validity was established by documenting that in ALS patients, change in ALSFRS-R scores correlated with change in strength over time, was closely associated with quality of life measures, and predicted survival [149-151]. The test-retest reliability is greater than 0.88 for all test items. The advantages of the ALSFRS-R are that the categories are relevant to ALS, it is a sensitive and reliable tool for assessing activities of daily living function in patients with ALS, and it is quickly administered. In a recent trial employing the ALSFRS as a secondary outcome measure, placebo treated patients showed a decline of 0.92 units per month, with a standard error of 0.08 [95]. With appropriate training the ALSFRS-R can be administered with high inter-rater reliability and test-retest reliability. The ALSFRS-R can be administered by phone, again with good inter-rater and test-retest reliability [152]. The equivalency of phone vs. in person testing, and the equivalency of subject vs. caregiver responses have also recently been established [153]. Therefore, if necessary, the ALSFRS-R may be given to the subject over the phone.

Survival: An alternative to time to death or PAV would be to time to death alone. In our experience these outcome measures will be similar because less than 10% of our patients elect PAV to prolong their lives. Both measures could be biased; the former because PAV prolongs peoples lives, independently of a treatment effect, and the latter because the time that PAV starts has a subjective element. A bias could result from an effect of treatment on these decisions. Since the subject won't know which treatment they are receiving the decision whether and when to initiate PAV is not likely to be affected by treatment. If the blinding of the treatment is ineffective, or the fraction of subjects with PAV among those with PAV or death is different between treatments then we will explore the sensitivity of our analysis to a treatment effect on PAV. In the absence of bias the outcome time to death or PAV is more powerful than using survival alone since there will be more events.

Survival in patients with ALS is affected by many factors in addition to disease modifying therapy. Nutritional issues, including timing and acceptance of gastrostomy tube are clearly important. No clear data currently exists regarding the extent to which gastrostomy tube placement prolongs survival, although current practice parameters suggest that gastrostomy should be considered before pulmonary function is clearly compromised [154]. Studies to determine the effect of gastrostomy on survival are in progress; however, it seems clear that quality of life is improved with appropriate placement [155-158]. Lacking data on the beneficial effect of gastrostomy tube placement on survival, site investigators will be asked to follow the AAN Practice Parameters. Similarly, although no large prospective trial has been performed, several small prospective and retrospective studies suggest that use of non invasive positive airway pressure ventilatory support prolongs survival in ALS patients [159-161]. For this reason, investigators will be asked to follow the practice parameter referenced above for non-invasive ventilatory support as well. Other factors influence survival in patients with ALS,

including age at diagnosis and site of onset [162-165]. In this study, we will not formally stratify for such variables, but will expect that randomization will result in equal distributions of sex and site of distribution, as has occurred in prior studies. We expect that the randomization will balance ancillary treatments among the treatment groups.

There will be two primary endpoints, survival and ALSFRS-R. There are several reasons for supporting two primary endpoints. First, survival requires long duration of study, and we are concerned with potential drop out with a long study. ALSFRS-R potentially is amenable to alteration over a significantly shorter period, so that late dropouts would have much less of an adverse effect on this measure. Secondly, we are powered to detect a smaller difference in disease progression for ALSFRS-R as compared to survival. We wish to maintain survival as a co-primary endpoint as we believe that ultimately this is the most meaningful endpoint for patients. Subjects who discontinue drug late in the study may still provide valuable data in a survival analysis as long as their survival is tracked. Using co-primary endpoints does not come at a great cost of power for either endpoint. Equal emphasis on both measures is appropriate.

Secondary Outcome Measures

Quantitative Strength Measurement Using Hand Held Dynamometry. In a recent study evaluating the efficacy of topiramate in ALS, maximum voluntary isometric contraction (MVIC) of 8 upper extremity muscles was employed as the primary outcome measure [95]. In this study, MVIC was performed after rigorous training and reliability testing. A significant change was noted in rate of change of muscle strength measured over 52 weeks; unfortunately, placebo treated patients performed better than those on active treatment. Arm megascore fell by 0.075 (± .0081) units per month in placebo treated patients, and 0.1 (± .0057) units per month in topiramate treated patients, declines that are similar to other studies. In the clinical trial of topiramate in ALS, a significant difference in MVIC was not associated with excess mortality over the treatment period, raising the question of how this measure is related to survival. Nevertheless, it is likely that muscle strength is an important determinant of both function and ultimate survival in ALS. Problems unique to MVIC are that it takes approximately 45 minutes to perform testing, requires expensive equipment, is not applicable for home visits and as patients get weaker data can not be obtained from very weak muscles. The time required for testing and the fact that visits to the study site are required likely contributed to the high subject dropout rate in the topiramate study, which was 30%. A faster, less fatiguing evaluation of strength is therefore desired.

Other more rapid and more portable methods of strength measurements are available. Manual muscle testing (MMT) involves measurement of muscle strength by a trained evaluator using standardized patient positioning, grading each muscle according to the MRC grading scaled used by most neurologists. If enough muscles are tested, a decline in average grade can be determined early in the disease, and the variability of measurement approximates that of MVIC [93]. The disadvantages of this technique are that the grading is qualitative, and, for any muscle, small changes are undetectable. A more promising technique uses hand held dynamometry (HHD) to test isometric strength of multiple muscles, again with standard patient positioning and rigorous training. This technique has been used previously in ALS clinical trials, as well as in trials in patients with inflammatory neuropathy and muscular dystrophy [166-169]. It has been directly

validated against MVIC in patients with ALS, and shown to change at a similar rate with variability that is only slightly greater than MVIC [170]. For both upper and lower extremity muscles, correlations between MVIC and HHD measurements ranged between 0.84 and 0.92, with test retest variability that was extremely similar as well. The only time where correlation between HHD and MVIC broke down was at high strength levels, an area not likely to be a problem in an ALS clinical trial. Hand held dynamometry takes less than 30 minutes to complete a test of both upper and lower extremities.

We therefore elected to employ this quantitative measure of muscle strength as a secondary outcome measure for this study. Six proximal muscle groups will be examined bilaterally in both upper and lower extremities (shoulder flexion, elbow flexion, elbow extension, hip flexion, knee flexion, and knee extension), all of which have been validated against MVIC testing [170]. In addition, wrist extension, first dorsal interosseuos contraction and ankle dorsiflexion will be measured bilaterally; while these muscles have not been directly compared using MVIC and HHD, they are muscles often affected in ALS, and HHD can be performed with low variability (Pestronk, personal communication). Mean and standard deviation for each muscle group will be established from the initial values for each subject in this trial, so that strength determinations can be converted to Z scores and averaged to provide an HHD megascore for both upper and lower extremities, similar to what is done for MVIC.

Hand Grip. Using the Jamar grip dynamometer, bilateral hand grip strength will be measured.

Vital Capacity (VC). The VC (percent predicted) will be determined. The rate of decline will be determined for each subject and treatment group. All clinical evaluators will be trained and perform reliability testing on patients with ALS prior to site initiation.

Quality of Life Scales (QOL). Previous studies using multiple instruments have shown that QOL does not correlate with disease severity, duration, or progression in patients with ALS. Therefore, it is evident that QOL in subjects with ALS is not easily determined by standard scales, such as the SF-36, that rely mainly on physical function as an indicator of quality of life. The scale we will be using is the ALS-Specific Quality of Life Scale (ALSSQOL). The ALSSQOL was developed, tested, and validated in subjects with ALS, and is not a health-related quality of life scale. The scale consists of 59 questions, each rated on a 1 to 10 scale, that ask about severity of the symptoms of ALS, mood and affect, intimacy, and social issues. In addition, the caregiver will also fill out a questionnaire, the Caregiver Burden Inventory (CBI). The CBI is a scale designed to rate the QOL of the caregiver, based on social, emotional, physical, time, and developmental aspects of the caregiver's relationship to the subject. The scale consists of 24 questions, rated 0 to 4 based on the caregiver's subjective feelings about their role as a caregiver and the effect of that role on the caregiver's quality of life.

Training and Validation. Training for all outcome measures will be accomplished at a centralized evaluator's meeting, or via individualized training from the outcomes trainer. In addition, training videos will be generated for secondary outcome measurements and written instructions will be provided to all evaluators. All evaluators must reach criterion levels of

intra rater reliability for each measure, as determined by the Outcome Measures Center. It is strongly preferred that a single evaluator perform all measures throughout the study; however if more than one evaluator is used at a single site, inter-rater reliability criteria will need to be met.

Safety and Tolerability Measures. The safety and tolerability of ceftriaxone were examined in STAGES 1 and 2 and will also be evaluated with longer treatment duration in the STAGE 3 trial. Safety is evaluated using vital signs, clinical laboratory determinations, reporting of adverse events, deaths and other serious adverse events, and treatment discontinuations due to adverse events. Information on adverse effects and on inter-current events will be determined at each visit by direct questioning of the subjects, clinical examination, and laboratory tests. Tolerability will be determined by the ability to complete the study on the assigned experimental drugs.

7. MANAGEMENT OF ADVERSE EXPERIENCES

Descriptions, monitoring and management plans for the most frequent and anticipated adverse events will be reviewed at the Investigator Meeting and during subsequent training sessions for new sites. This information will also be provided to all sites in the Manual of Operations (MOP). Adverse event monitoring and management plans will be updated in the MOP as needed throughout the course of the study. Expected adverse events from ceftriaxone are listed alphabetical order below in section 7.1. Expected adverse events from ursodiol are listed in alphabetical order in section 7.2. Expected adverse events from the Central Venous Catheter are listed in alphabetical order in section 7.3. . Adverse Events that require discontinuation of study drug are described in section 8: *Criteria for Intervention Discontinuation.*

7.1 Adverse Experiences Related to Ceftriaxone Use

Monitoring and management plans for each of the following, potentially serious, adverse experiences related to ceftriaxone use are contained in the *Adverse Event Management* section of the MOP. If any of the following adverse events are suspected, the appropriate management plan should be reviewed immediately.

Acute Allergic Interstitial Nephritis

Acute allergic interstitial nephritis (AIN) occurs when inflammatory cells infiltrate the interstitial compartment of the kidney without affecting the glomeruli. Delayed treatment of AIN may result in renal failure. Acute allergic interstitial nephritis occurs in less than 1% of patients treated with ceftriaxone [103]. Diagnostic workup may reveal eosinophilia and elevated serum creatinine. Urine studies may demonstrate hematuria, proteinuria, white cell casts in the urine, eosinophiluria, fractional excretion of Na < 1%, and oliguria. By ultrasound, the kidneys may be increased in size [171]. If acute allergic interstitial nephritis is suspected, the site nephrologist should be consulted.

Acute Tubular Necrosis

Acute tubular necrosis (ATN) results from ischemia of the renal tubules or from exposure to nephrotoxic drugs. Pathologically, this causes a combination of intrarenal vasoconstriction, direct tubule toxicity, and/or intratubular obstruction [171]. Delayed treatment of ATN may result in renal failure. Acute tubular necrosis occurs in less than 1% of patients treated with ceftriaxone [103]. Acute tubular necrosis is characterized diagnostically by muddy brown granular or tubular epithelial casts in the urine, fractional excretion of sodium (Na) > 1%, urine Na>20 mEq/L, and specific gravity = 1.010. Serum studies may show an acute rise in serum creatinine (daily rate of rise from 0.5 to 1.5 mg/dL), hyperkalemia and hyperphosphatemia [171]. If acute tubular necrosis is suspected, the site nephrologist should be consulted.

Biliary Sludge

Biliary sludge may occur with long-term ceftriaxone use, but is rare with short-term use and occurs in less than 0.1% of patients treated [103]. It may occur more frequently with long-term use. Often, patients are asymptomatic. Symptoms of biliary tract disease, such as acute cholecystitis, are rare. When symptoms of biliary tract disease do manifest, they can present as right upper quadrant abdominal pain, tenderness, fever, jaundice and elevated bilirubin and alkaline phosphatase [172]. Biliary sludge can be diagnosed by abdominal ultrasound. Subjects with cholelithiasis diagnosed by abdominal ultrasound can be referred to a site gastroenterologist. All subjects will undergo abdominal ultrasounds at Week 4, Week 8, and Week 20. A detailed management plan is provided to the sites in the manual of operations.

Candidiasis

Candidiasis is associated with concurrent antibiotic use. This may present as mucocutaneous or rarely invasive disease (e.g. tissue dissemination, skin lesions, ocular disease, or osteomyelitis) [173]. Genital candidiasis occurs in less than 1% of patients treated with ceftriaxone [103]. Diagnosis of fungal infection involves visual inspection for diagnosis of thrush. If not entirely clear, microscopic examination of mouth scrapings or culture can be done. Genital candidiasis is diagnosed by KOH preparation or wet prep of vaginal secretions**.** Invasive candidiasis/sepsis involves more extensive monitoring of vital signs and serum white blood cell count. Complete blood count with differential, blood culture, blood pressure, temperature should all be evaluated [173].

Clostridium difficile Infection and Diarrhea

Diarrhea occurs in 2.7% of patients treated with ceftriaxone [103]. Subjects who note onset of diarrhea should be fully evaluated for the underlying etiology. Twenty to 30% of all cases of antibiotic-associated diarrhea are the result of *Clostridium difficile* infection [174].
If *C. difficile* infection is suspected, ELISA (enzyme-linked immunosorbent assay) test of stool should be checked. Stool culture is the most sensitive method of detecting *C. difficile* infection. However, false positive results are common due to the presence of non-toxigenic strains in the stool. While ELISA testing is less sensitive (80%), it is a same day assay, less expensive, and can be performed more easily [174].

Cholelithiasis

Cholelithiasis occurs in less than 0.1% of patients undergoing short-term treatment with ceftriaxone [103]. Diagnosis can be made by abdominal ultrasound. Gallbladder stones may be accompanied by gallbladder wall thickening or edema. Gallstone pancreatitis has been reported with ceftriaxone therapy in case reports [175-177]. In a study by Lopez et al, pancreatitis was caused by gallstones [178]. These stones consisted of 80% ceftriaxone and 20% bilirubin.

Subjects with cholelithiasis diagnosed by abdominal ultrasound can be referred to a site gastroenterologist. All subjects will undergo abdominal ultrasounds at Week 4, Week 8, and Week 20. A detailed management plan is provide in the site manual of operations.

Dehydration

Dehydration may occur while on the study drug, most often as a consequence of diarrhea.

Mild dehydration is characterized by less than 5% body weight loss, pallor, and normal blood pressure. Moderate dehydration is defined by 5 to 9% body weight loss, dry mucosa, orthostasis and reduced skin turgor. Severe dehydration is characterized by more than 10% body weight loss, cool extremities, hypotension, tachycardia and mottled skin [179].

Subjects will be asked about hydration at each study visit. Subjects will be asked if they have experienced any vomiting or diarrhea, the number of episodes, and the duration of the symptoms. They will also be asked if they have had symptoms of dry mouth, skin pallor, weight loss, cool extremities, dizziness, rapid heartbeat or infrequent urination.

Leukopenia, Thrombocytopenia, and Prolonged Prothrombin Time

Leukopenia can occur with ceftriaxone use [103]. Complete blood counts will be monitored during study drug treatment. If the neutrophil count falls below 1200, the study drug will be held. Re-initiation of the study drug will be considered in consultation with the site infectious disease consultant.

Thrombocytopenia and Prolonged Prothrombin Time (PT) are rarely reported (less than 1%) side effects that may lead to bleeding (hemorrhage).

Super Infection

Prolonged use of ceftriaxone may result in overgrowth of nonsusceptible organisms. Careful observation of the subject is essential. If superinfection occurs, appropriate measures should be taken.

Interaction with Calcium-Containing Products

There are no reports of intravascular or pulmonary precipitations in patients, other than neonates, treated with ceftriaxone and calcium-containing IV solutions. However, the theoretical possibility exists for an interaction between ceftriaxone and IV calcium-containing solutions in patients other than neonates. Therefore, Ceftriaxone injection, USP and calcium containing solutions, including continuous calcium-containing infusions such as parenteral nutrition, should not be mixed or co-administered to any patient irrespective of age, even via different infusion lines at different sites. As a further theoretical consideration and based on 5 half-lives of ceftriaxone, Ceftriaxone Injection, USP and IV calcium-containing solutions should not be administered within 48 hours of each other in any patient.

7.2 Adverse Experiences Related to Ursodiol

Ursodiol is FDA approved for the chemodissolution of bile duct stones, gallstones, prophylaxis of gallstones during rapid weight loss and primary biliary cirrhosis. Contraindications include hypersensitivity to bile acids or ursodiol. Adverse events occurring more frequently with ursodiol than placebo include diarrhea (1.3%), elevated creatinine (1.3%), elevated blood glucose (1.2-1.3%), leukopenia (2.6%), peptic ulcer (1.3%) and skin rash (2.6%)[180].

7.3 Adverse Experiences related to Central Venous Catheter

Arterial Puncture and Bleeding

During the insertion of the CVC there is a potential for inadvertent arterial puncture. This occurs in 8.4% of radiologically placed catheters. Bleeding occurs in 0.4% of placements of tunneled catheters [131].

Hematomas and bleeding are generally treated conservatively with firm pressure. Most percutaneous arterial punctures pose few complications and most are often not reported [131].

Deep Vein Thrombosis

The mechanism of catheter thrombosis is poorly understood, but is thought to be related to vascular injury from the catheter. The incidence of symptomatic thrombosis of a tunneled catheter occurs at a rate of 0.06 per 1000 days use for homecare patients [122]. The risk of a symptomatic thrombosis developing is more likely to occur within the first week of placement of the catheter, and again after it has been in place for more than 30 days [122, 132]. Patients who have developed one catheter thrombosis are at greater risk of developing future thrombosis [135].

Upper extremity deep vein thrombosis often present with upper extremity, neck and facial edema. The extremity may be cool and a low-grade fever or tachycardia may be present. Half of all patients with deep vein thrombosis present with no symptoms. Ultrasonography is highly predictive of detecting distal deep vein thrombosis and is more reliable than V/Q scan or impedance plethysmography. Contrast venography may be diagnostic for more central lesions [106].

Central venous catheters should be removed in the event of deep vein thrombosis. The incidence of pulmonary embolus is increased if left in place, even when therapeutic anticoagulation is obtained [181].

Exit Site Infections

Infections of the tunneled catheter exit site occurs 0.36 incidences per 1000 days of use in the homecare setting [122]. These infections may result in cellulitis, abscess or fasciitis.

Wound infections are generally diagnosed clinically, with the presentation of tenderness, warmth, induration, erythema, drainage and fever. Wound drainage should be cultured.

The study drug can be maintained and the central venous catheter kept in place if the subject develops a local exit site infection, unless otherwise determined by an infectious disease specialist.

Each site will be required to have an Infectious Disease specialist available as a consultant and for clinical consultation. Any subject who presents with clinical signs and symptoms of infection, either at exit site or intravascular infection, will be referred to an infectious disease specialist for clinical evaluation.

Functional Problems

With prolonged use, or through accident, the catheter may crack, break, infiltrate or become partially or completely withdrawn. Using metal clamps, repeated clamping over the same site, using sharp objects around the catheter and failing to properly secure the catheter can lead to these problems.

The use of the study drug should be held until the issue of the catheter's integrity and sterility is resolved. If the catheter is removed, the study drug can be continued through a peripheral line or by intramuscular injection temporarily.

Intravascular Related Infections

Systemic infections related to CVCs result from both tracking of bacteria along the tunneled aspect of the catheter and by contamination of the catheter hub [125-129]. Organisms that typically cause these infections are Staphylococcus aureus, S. epidermidis, streptococcus species, gram-negative bacilli and Candida spp. [127-129]. The incidence of catheter-related bloodstream infections was identified as 0.34 for every 1000 days of use in patients with catheters being cared for at home [122].

When a systemic bloodstream infection is present, patients may present a clinical picture of fever, rigors, hyperventilation, malaise and a general toxic appearance. A leukocytosis is often present and there may be pain or drainage at the catheter insertion site. A new heart murmur may be evident on physical exam. Multiple blood cultures obtained peripherally and centrally

via the catheter may confirm the diagnosis. Other sources of infection are ruled out through physical exam, chest x-ray, blood and urine laboratory data, including urine culture, and through imaging studies that are clinically indicated.

If a systemic infection is suspected, the study drug may be continued or discontinued as recommended by an infectious disease expert. Study drug can be given either through the central catheter or through a peripheral line. Antibiotics can be empirically initiated, but should not include another cephalosporin. Treatment should be guided by the organism or organisms identified in the blood cultures. The catheter should be removed when there is evidence that the subject is hemodynamically unstable or if S. aureus or a fungemia is present. The catheter may need to be removed if coagulase-negative staphylococcus or a gram-negative organism is identified, based on the clinical judgment of infectious disease consultant. Once the infection has resolved, the study drug may be resumed.

Any subject who presents with signs and symptoms of intravascular infection will be referred to an infectious disease specialist for clinical consultation.

Catheter Thrombosis

The formation of thrombin sheaths occurs in as high as 66% of central catheters. However, only 6% of these identified cases go on to develop and symptoms [133]. Typically, dysfunction in the ability to aspirate or infuse into the catheter is initially seen, and may lead to complete obstruction if not treated [135].

The patient is asymptomatic when thrombosis is confined to the catheter. The only signs may be the inability of the catheter to freely infuse or aspirate. Upper extremity edema may indicate a deep vein thrombosis and this should be ruled out, if present.

The catheter should not be removed, unless recommended by interventional radiology. If the catheter is removed, the study drug may be temporarily suspended or given peripherally.

Chest Wall Discomfort

As with any surgical procedure, some chest wall discomfort from the placement of the catheter is to be expected. Discomfort or tenderness that exceeds the expected severity or time involved for healing should be investigated further.

Pneumothorax

Pneumothorax is a problem that can develop while placing the catheter. It occurs at a rate of 0-3.3% when radiologically placed. Incidence of pneumothorax appears more frequent with the use of the subclavian vein and rare with the use of the jugular vein [131].

Placement of a tunneled catheter is followed by radiographic evaluation to rule out any pneumothorax, symptomatic or not. Intervention is determined by the degree of pneumothorax.

Pulmonary Embolus

Catheter-related incidence of pulmonary embolus occurs in 12-36% of all cases of upper extremity deep vein thromboses related to catheter use [130]. The risk of air embolus has been assessed as between 0.6-0.8%, with most of these being small and asymptomatic. Serious adverse effects from air embolus are infrequent [131].

Pulmonary embolus is often difficult to recognize clinically, which contributes to its high mortality. Diagnostic symptoms may include anxiety, dyspnea, chest pain, tachycardia, diaphoresis and tachypnea.

In the event of a pulmonary embolus, the central catheter should be removed because of the higher incidence of the catheter causing additional emboli [181]. If the subject is clinically stable, the study drug can continue or it can be resumed via a peripheral line temporarily.

8. CRITERIA FOR INTERVENTION SUSPENSION, RECHALLENGE, OR PERMANENT DISCONTINUATION

8.1 Intervention Suspension Related to Ceftriaxone Use

Study drug will be suspended under the following circumstances:
(Please refer to the Manual of Operations for details regarding management of AEs)

- If the subject has *C. difficile* infection and is concurrently systemically ill with fever, leukocytosis or if the symptoms are refractory to treatment
- If the *C. difficile* infection results in pseudomembranous colitis
- If the subject is being treated for severe dehydration
- If the subject is symptomatic from biliary sludge
- If the subject is diagnosed with symptomatic cholelithiasis
- If the subject is diagnosed with invasive candidiasis
- If the subject is diagnosed with acute interstitial nephritis
- If the subject is diagnosed with acute tubular necrosis
- If the subject is diagnosed with renal failure
- If the serum creatinine doubles as compared to the screening visit value, the test will be repeated. If the value is confirmed (doubled as compared to screening visit value), study drug will be suspended while subject is evaluated.
- If the urinalysis is positive for Wright’s or Hansel’s stain as this is suggestive of eosinophiluria, or allergic interstitial nephritis
- If the spot urine protein is greater than 1000 mg. This is likely an indication of severe renal toxicity
- If the subject becomes oliguric, which is defined as urine output less than 500 mL/day. This can be an indication of severe renal toxicity
- If the Absolute Neutrophil Count (ANC) is less than 1.2 th/cmm

Subjects may be rechallenged with study drug under the following circumstances:

- Upon resolution of pseudomembranous colitis at the discretion of the site investigator
- Upon resolution of dehydration at the discretion of the site investigator
- Upon resolution of symptomatic biliary sludge
- Upon resolution of symptomatic cholelithiasis
- Upon resolution of *C. difficile* infection
- If absolute neutrophil count (ANC) returns to normal range

Subjects can not be rechallenged with study drug under the following circumstances:

- If the subject is diagnosed with invasive candidiasis
- If the subject is diagnosed with AIN or ATN
- If the subject is diagnosed with renal failure
- If the spot urine protein is greater than 1000 mg

8.2 Intervention Suspension Due to Catheter Complications

Study drug will be suspended temporarily under the following circumstances:

- If the subject is hemodynamically unstable
- If suspension is recommended by Infectious Disease
- If the catheter is unable to infuse
- If the catheter is accidentally removed or if its integrity is in question
- If the subject and family need further education to conform to the standards of safety and sterility

The catheter will be removed under the following circumstances:

- If there is a proven sepsis from Staphylococcus aureus, the presence of a fungemia or if the subject is hemodynamically unstable due to, or presumed to be due to, catheter sepsis.
- If the catheter is accidentally withdrawn to the point that it is no longer in a large vein which can sustain long standing intravenous therapy
- If the care provided by the subject and caregiver, in spite of attempts at re-education, does not meet the basic standards of safety and sterility
- If there is a demonstrated deep vein thrombosis
- If there is a high clinical probability of, or, demonstrated pulmonary embolus felt to be thrombosis related
- If removal is recommended by Infectious Disease or Interventional Radiology

9. STATISTICAL CONSIDERATIONS

9.1 General Design, Data Analysis and Power

The senior biostatistician at Massachusetts General Hospital will be primarily responsible for the biostatistical component of this study. The pharmacology consultant at Tufts University School of Medicine is primarily responsible for the pharmacokinetic analysis in STAGE 1.

9.1.1 STAGE 1 Pharmacokinetic Analysis and Sample Size

The primary purpose of the STAGE 1 study will be to determine if the 20th percentile of the trough level is significantly greater than 1μM. This would imply that at least 80% of the subjects would receive a minimum dose of 1μM. We intend to use a log-normal model for this calculation. Using a normal approximation, with 20 subjects, we would have an 80% chance of rejecting the null hypothesis that the 20th percentile of the trough level is 1μM or less at a two sided p = 0.05 significance level if the true trough level was 1.44μM or more, based on a 50% coefficient of variation in trough levels. Based on a preliminary analysis of ceftriaxone in CSF [84], we were able to project the steady state trough levels by fitting the t-max, cmax, and $AUC_{0-\infty\infty}$, and half life given in Appendix 1 (Table 1) to a model where CSF ceftriaxone concentrations are assumed to be consistent with a one-compartment model, with first-order entry (rate constant: K_a) and exit (rate constant: K_e) from the CSF compartment. Based on that data the trough levels for a once a day dose would be 1.44 μM and for a twice a day dose would be 3.22 μM.

The secondary purpose of the Pharmacokinetic study is to determine the population pharmacokinetics of ceftriaxone in plasma and CSF. Knowledge of the distribution of the pharmacokinetic parameters will allow us to estimate the effect of a weekend drug holiday for the subjects, other alterations in the dosing schedule and the mean and standard deviation of the average exposure to the drug. Since this estimation may be difficult given data on trough levels and only one other time point, we are prepared to use three different estimation techniques. We plan to model CSF concentrations alone and jointly with plasma levels [182]. Ideally, a joint model will allow us to predict CSF levels using plasma levels. The three methods are NONMEM, which would be performed by our pharmacology consultants, who have experience using this program and the R function NLME [183] and PKBugs [184], which would be performed by our senior statistician . NONMEM and NLME both fit a random effects model to the data. The program PKBugs uses a Bayesean framework and should lead to more stable estimates because prior information from a full pharmacokinetic study will be utilized. The prior distribution of the parameters will be based on the data in Nau and colleagues [84]. In the final model we will consider covariates such as subject gender, height, weight and extent of disease.

Plasma Pharmacokinetics. Based on existing data, it is assumed that plasma kinetics of ceftriaxone are consistent with a two-compartment model. Accordingly, plasma concentrations are analyzed by derivative-free weighted nonlinear least-squares regression analysis (SAS PROC NLIN). The following equation is fitted to the data points:

$$C = [Q/V_1][(k_{21}-\alpha)(1-e^{\alpha T})(e^{-\alpha t})/\alpha(\alpha-\beta)] + [Q/V_1][(k_{21}-\beta)(1-e^{\beta T})(e^{-\beta t})/\beta(\beta-\alpha)]$$

with appropriate adjustment for the acquisition of data at steady-state in the course of multiple-dose treatment.

In this equation, C is the plasma ceftriaxone concentration and t is the time after the start of the infusion. T is an additional variable dependent on the infusion duration. T = t at all points in

time during the infusion; after the infusion is complete, T becomes fixed at a value equal to the duration of the infusion. Q is the zero-order ceftriaxone infusion rate, and V_1 is the apparent volume of the central compartment. The exponents α and β, having units of reciprocal time, are hybrid rate constants (derived from the parameters of the two compartment model) representing the apparent distribution and elimination phases of the plasma concentration curve, respectively. K_{21} is the apparent first-order rate constant of return from peripheral to central compartments. The fixed values in the analysis are Q and the infusion duration. The iterated variables are: V_1, α, β and K_{21}. These are used to calculate the following: total volume of distribution using the area method (V_d), apparent half-life of distribution and elimination, and total clearance [185].

CSF Pharmacokinetics. Initially we model CSF and plasma concentrations separately. The CSF parameters below will therefore have different values than the plasma parameters above. From the study by Nau, CSF ceftriaxone concentrations are assumed to be well approximated with a one-compartment model, with first-order entry (rate constant: K_a) and exit (rate constant: K_e) from the CSF compartment C= (D/V)*ka*(Exp(-ke*t)-Exp(-ka*t))/(ka-ke). For population models, we would model V, and ke as having a log normal between subject distribution. To obtain a preliminary estimate of the variability in our estimates of pharmacokinetic parameters and to determine the best time points for the second sample, we plan to conduct simulation studies. From Nau et al. we estimate ka=0.116/hr, ke=0.04/hr. (1/V)=0.00044305/L. We would generate 100 data sets of 20 subjects on once a day 2 gram dosing and 20 subjects on twice a day 2 gram dosing. Population pharmacokinetics would be generated from each data set. The between subject coefficient's of variation, and the measurement error would be assumed to be similar to other studies. We would perform simulations for the combined doses and for each dose separately with various parameter choices. We conducted a pilot simulation to see if the proposed model was estimable. We generated data based on parameters estimated from the Nau (ka=0.116/hr, ke=0.04/hr. (1/V)=0.00044305/L) and picked the time points at random during the first 12 hours. We found that all of the parameters, V, ke, ka, the population standard deviations and the error variance were estimable. The value of tmax calculated from these parameters had a standard error of 2 hours and the cmax had a standard error of 0.06. We conclude that our analysis plan is reasonable and will estimate the parameters with enough precision for our purposes.

Joint Pharmacokinetic Model. Our initial model would be to use the plasma model described above and assume that there are two additional parameters representing diffusion from the plasma into the CSF and from the CSF back into the plasma.

9.1.2 STAGE 2 Safety and Tolerability Study

In accordance with the intent-to-treat principle, all subjects randomized will be kept in their originally assigned treatment group for analysis. All randomized subjects will be considered evaluable for tolerability and safety. In the tolerability analyses, subjects will be regarded as a treatment failure if they fail to complete Week 20 of the study on the originally assigned treatment for any reason. The decision will also involve the result of the pharmacokinetic study, since we wish to choose a dosage where the trough levels are greater than 1μM. We consider several possible scenarios, and the decision rules that might be used. The data will be reviewed

by the Steering Committee, using the considerations here as guidelines. Their recommendation would then be reviewed by the DSMB.

We will consider a dosage tolerable if the proportion of treatment failures for a dosage is less than 40% with 80% confidence. With 20 subjects this would occur if 5 or fewer subjects fail to complete the 20-week study. With 20 subjects we will have more than an 80% chance of declaring a dosage tolerable if the true treatment failure rate is 20%. If both dose regimens are tolerable and both achieve 1μM and there are no serious dose related adverse events on the higher dosage and no differences at the one-sided p=0.10 level between adverse event rates that are deemed important than we will use the twice a day dose for the remainder of the study. This would also be the regimen of choice if the one a day regimen did not achieve 1μM and the twice a day regimen was tolerable. Otherwise, we would choose the once a day regimen if it were tolerable and achieved 1μM. If the regimen that achieved 1μM was not tolerable or no regimen achieved 1μM we would consider ending the trial. The decision at this point might involve knowledge of the other pharmacokinetic parameters since if the average dose is high enough it may be worthwhile to continue. In addition we may find that we have equivocal data on the tolerability of whatever regimen achieves 1μM. This would require us to continue the second stage of the study beyond 60 subjects. For instance this might happen if 5-10 subjects in the dosage that achieved 1μM did not tolerate therapy or we had to make substantial changes in the protocol during the first 20 weeks.

An additional purpose of the STAGE 2 study is to determine serious adverse effects of treatment while the study is being conducted by only a few centers. With 20 subjects we will have an 80% chance of seeing at least one occurrence of any adverse event that occurs with a frequency of 8% or more.

9.1.3 STAGE 3 Efficacy Trial

There will be two co-primary endpoints, survival and ALSFRS-R. Both survival and ALSFRS-R endpoints will be tested using a p-value of 0.05, as discussed in O'Brien [186].. We have chosen not to use an explicit p-value adjustment for these two co-primary endpoints. We are considering the question of whether Ceftriaxone preserves functional outcome (ALSFRS-R) and whether it improves survival as separate questions.

Analysis of Survival

The primary analysis will be by "intention to treat." We will ascertain the status of all subjects at the end of the study and every subject will be included in the analysis whether or not they elected to stop treatment before the end of the study. The analysis of survival will use a log-rank test stratified by riluzole use.

A sample size of 600 subjects will provide us with over an 80% power to detect a 50% increase in median time to death, tracheostomy or PAV, from 29 months to 44 months for subjects taking the drug. In terms of the one-year survival rate this would be a change from a 75% one-year rate to an 83% one-year rate, or a change from a 24% five-year rate to a 38% five-year rate for patients taking the drug.

The trial will be monitored every six months for efficacy and futility. Stopping for efficacy will only occur if the survival comparison exceeds boundaries described below. Decisions on futility will depend on both primary endpoints, as well as the realized accrual of subjects into the study and the frequency of subjects discontinuing study medication.

Since there are a very large number of sites that will be enrolling subjects, there will not be sufficient power in the study to examine the effects of each site individually.

Stopping for Efficacy

Efficacy stopping will use the alpha spending rule approach which allows flexible choice of monitoring times. The basic idea is that the trial can “spend” (or allocate) a one-sided Type one error of 0.025(equivalent to a two-sided p=0.05 test) over the course of the trial. Thus one can pre-define a “spending” function which gives the amount of alpha spent as a function of the proportion of the total number of events that have occurred by the time of the interim analysis. We will schedule the interim analyses as defined below with an alpha spending function which depends on the proportion of events, denoted by t (so called information time), that has occurred by that analysis. The alpha spending function we will use is $0.025\ t^3$. We will assume that the total number of events on the trial will be 293, this is the number expected based on a yearly survival rate of 75%, for the purposes of computing t although this may be adjusted using the guidelines in Proschan, Lan and Wittes [187]. The table below gives the efficacy stopping guidelines for the trial based on our assumptions about the accrual and mortality rate. The two event rate estimates are calculated under the alternative and null hypothesis assuming a one year survival of 75%. We assume that the accrual will take five months to ramp up to 25 patients per month. The p-value to stop is one-sided. The first meeting three months after the start of accrual for STAGE 3 (Study Month 3) will have the purpose of reviewing the safety of Ursodiol use and any other new procedures of the STAGE 3 portion of the study.

Study Month	Accrual	Number of Events	p-value to stop
3	NA	NA	NA
9	235	31-40	0.000064
15	385	61-75	0.000374
21	535	102-125	0.001686
27	600	153-187	0.005537
33	600	201-244	0.011807
39	600	243-293	0.019872

For example, by month 15, the study should have accrued 385 subjects, and between 61-75 events (death/PAV) will have occurred. A log-rank test will be carried out to compare survival between the placebo group and group of treated subjects. If this log-rank test leads to a one-sided p-value of less than 0.000374 then the study can be stopped for efficacy.

Analysis of ALSFRS-R

Our primary analysis strategy is to use the “shared parameter” model described in Vonesh, Greene and Schluchter, 2006 [148]. They propose a random effects analysis of variance to model

the change in ALSFRS-R over time. The basic idea of this model is that each subject has his or her own trajectory with a random slope, intercept and curvature, the average value of which may depend on treatment. The primary analysis of ALSFRS-R will include measurements made during the first 12 months only, to minimize the effect of drop out. They extend this model to include a parametric model for mortality during the 12 month period that we are analyzing. This corrects the estimate of the treatment effect for loss of data which might be dependent on the subject's ALSFRS-R trajectory. We will model survival by assuming that the mortality distribution is Weibull with a scale parameter that depends on the random slope and intercept of ALSFRS-R. We plan to continue follow up of subjects who discontinue treatment so that we should have ALSFRS-R at all of the time-points on the vast majority of subjects except for those that die. As a sensitivity analysis, we will also correct for subjects who are missing their final ALSFRS-R measurements using a modification of the model described above. This model can be modified by having two causes of "failure", death and "missing further data". Then both of these outcomes can be modeled using a Weibull model as above and the difference in the slopes due to treatment can be calculated, corrected for dropouts and deaths which may be associated with ALSFRS-R.

The primary analysis will use a p-value of 0.05. There will be no efficacy stopping boundaries for ALSFRS-R. We don't include an efficacy stopping boundary for ALSFRS-R because if ALSFRS-R is strongly positive it will indicate that with enough follow up and subject accrual we may eventually see a survival benefit. Since measuring the survival benefit, if there is one, is a primary goal of the trial we would not want to stop the trial if there is a good chance of seeing a survival benefit. Futility boundaries are described below. With the sample size of 600 subjects and a 2:1 randomization we will have 80% power to see a 25% decrease in the rate of ALSFRS-R decline in either treatment group.

Stopping for Futility in STAGE III

Stopping for futility could occur if the trial doesn't reach accrual targets or if both ALSFRS-R and survival do not show sufficient trends towards efficacy. In addition if the dropout rate is so high that the power to show a survival benefit is minimal, then the primary endpoint of the trial will be changed to compare 12 month changes in ALSFRS-R and subjects will be given the option of discontinuing study medication at 12 months.

Accrual Targets: The study should take five months to begin fully accruing at a rate of 25 subjects per month. If the study does not reach at least 60% of its accrual goal at any interim analysis then the study will have reached an accrual futility boundary. At this point the DSMB can stop the study for accrual.

Efficacy Targets: Both Survival and the slope of ALSFRS-R will be used to determine a futility stopping boundary. The trial will be considered futile if both ALSFRS-R and fail to cross the futility boundary specified by a beta spending function .05*t. The table below shows the p-value targets (one sided p-values) for these measures. For an endpoint such as ALSFRS-R, it cannot be defined as the proportion of subjects that have entered the trial because some subjects may not have more than a few ALSFRS-R measurements. Its formal definition is the ratio of the variance of the treatment time interaction at the final analysis over the variance at the interim analysis.

The information time for the ALSFRS-R is assumed to be proportional to .75 times the number of subjects plus .25 times the number of subject years of follow up. This was calculated by a linear regression of the variance of the efficacy parameter on various combinations of sample size and follow up period.

Treatment Discontinuation Rate Targets: All analyses will be by "intent to treat", but the effect of subjects' discontinuation will be to reduce the power of the study as the treatment effect will be diminished by subjects in the treatment group who don't continue treatment. This effect will be largest in the survival comparison and will have a minor effect on ALSFRS-R which is only used for the first year to judge efficacy. The hazard ratio used in the power calculation is an "intent to treat" hazard ratio, it reflects the hazard ratio achieved by an effective treatment on all participants combined whether or not they are completely compliant and are treated for the whole period. The "as treated" hazard ratio, the hazard ratio achieved by an effective treatment if all patients are completely compliant, would need to be higher than the "intent to treat" hazard ratio. The relationship of the two hazard ratios does not only depend on the number of subjects that discontinue treatment but on when they discontinue. In the simplest model, described below, it depends on the proportion of time that subjects are treated. If the dropout rate does not meet the treatment discontinuation target that subjects are treated significantly less than 2/3 of the time the recommendation would be to use ALSFRS-R as the primary endpoint and only follow each subject for one year. Survival data would still be collected but would not be expected to be impacted significantly by treatment. The following paragraphs give a justification of this target.

Under a Poisson event model, the primary effect of treatment discontinuation can be measured by the ratio, p, of the follow up that a patient is on treatment divided by their total follow up**.** . If p is the proportion of time that a subjects is on treatment divided by the total time they are included in the study, then the "intent to treat" hazard ratio will be approximate 1/(p/R+(1-p)) where R is the on-treatment hazard ratio (Placebo/RX) . The parameter p will be estimated by the sum of the area under the time to discontinuation curves for each subject's potential follow up divided by the sum of the area under the time to death curve. To project the value of p for the entire study it is necessary to know these beyond the current follow up period. This will require a parametric model. At each DSMB meeting we will report on the current estimate of p. If p is significantly (p=0.05 one sided) less than 0.67 the DSMB may recommend that the trial no longer have survival as a primary endpoint and be redesigned to focus on ALSFRS-R measured for 12 months.

The justification of this target is as follows. If p=0.67, that is, a subject is on treatment for 67% of the time they are in the study, and R=2 than 1/(p/R+(1-p))=1.5, which means that subjects while being treated will survive for 1.5 times longer than untreated subjects. Thus an on-treatment hazard ratio of 2 would give an intent-to-treat hazard ratio of 1.5. We feel that an on-treatment hazard ratio of 2 is feasible but any higher hazard ratio is unlikely to be met, so our target for p will be set to 0.67, that is, all treated subjects would be required to remain on their treatment for at least 67% of the total time they are in the study.

Feasibility/Futility Targets:

Study Month	60% of Accrual Target	P-value survival	p-value ALSFRS-R
3	NA	NA	NA
9	141	0.91	0.60
15	231	0.82	0.37
21	321	0.62	0.18
27	360	0.39	0.12
33	500	0.24	0.12
39	600	NA	NA

For example, on month 15, we require that at least 241 patients have accrued and that the p-value for survival be less than 0.82 or the p-value for ALSFRS-R be less than 0.29. We also we calculate the estimated value of p and it's standard error described above and require that it is significantly better than 67%.

The effect of a 2:1 randomization on power is relatively small. Suppose n is the total sample size of a clinical trial. Then the standard deviation of the test statistic measuring a treatment effect is roughly $\sqrt{1/(n/2)+1/(n/2)} = \sqrt{4/n}$ with equal allocation to both treatments. With a 2:1 allocation it is \sqrt{1/(2n/3)+1/(n/3)}=\sqrt{9/2n}. Thus the ratio of these is $\sqrt{8/9}$. The effective sample size is thus 8/9 of the sample size if we go from a 1:1 randomization to a 2:1 randomization. This only has a small effect of the extent of a difference that we can detect.

Analysis of Secondary Outcome Variables

Safety and Tolerability. The safety data will be summarized by treatment group. The treatment groups will be compared with respect to occurrence of each adverse event. Total number of adverse events and abnormal laboratory tests will be compared between groups using Fisher's exact test. Withdrawal, abnormal laboratory tests, vital signs and use of concomitant medications will be assessed to characterize the safety profile of ceftriaxone. Compliance data will be determined for each visit and by treatment group. The time to subject refusal will be compared between treatment groups in order to better determine tolerability. This will be accomplished using a method of survival analysis that allows informative censoring due to death [188]. Descriptive statistics denoting the changes from baseline to the final assessment visit with respect to key laboratory parameters and vital signs will also be provided.

VC, Dynamometry and Quality of Life. Our primary analysis strategy is to use a random effects analysis of variance using PROC MIXED in SAS [189]. The basic idea of this model is that each subject has his or her own trajectory with a random, slope, intercept and curvature the average value of which may depend on treatment. Since the subject groups start out the same, the intercept will not depend on treatment but the slope and curvature may. In our trial of topiramate this model was sensitive to a negative effect on muscle strength on treatment.

There was a significant curvature but only the slope was affected by treatment. The advantage of using this model is that it accommodates the variable duration of follow-up that will result from our trial design. We will look carefully at the residuals for this model to be sure that it fits the data. If there appear to be problems with fit, we will use a robust approach of assessing significance where the p-value for any treatment contrast is calculated using a permutation test.

To determine how large an effect on FVC and dynamometry we could detect, we calculated these parameters of this model from the placebo group of our 12-month topiramate study. We did not have dynamometry in that study so we substituted MVIC. Based on the estimates from this study we would have an 80% chance of showing significance at a two-sided p=0.05 level if the true rate of change in the treatment group was a 17% decrease in the rate of decline of the outcome. Interestingly this was the same for all three of the outcomes. The analysis of the extent to which FVC and dynamometry are surrogates for time to death, tracheostomy and PAV will be conducted using the methods of Shah and colleagues [190].

Analysis of Data on Women and Minorities. Our proposal for the analyses of these data follows NIH Guidelines. We intend to use the data from the trial to determine whether women or minorities respond differently to treatment. This will be accomplished by testing for a treatment gender and treatment ethnicity interaction in our efficacy and safety analyses. Although the power of these analyses may be low for the primary outcome the power will be adequate for the secondary outcomes.

9.2 Outcomes

Section 9.1, above, contains detailed information regarding Outcomes

9.3 Sample Size and Accrual

Section 9.1, above, contains detailed information regarding Sample Size and Accrual

9.4 Data and Safety Monitoring

9.4.1 Monitoring and Reporting Process

The Principal Investigator is responsible for oversight of the data safety and will work with the Co-PIs to monitor the proposed study. The stopping rules for futility and efficacy are described in detail in Section **9.1.3.** In summary, the trial will be stopped for futility if the trial doesn't reach accrual targets or if both ALSFRS-R and survival do not show sufficient trends towards efficacy. Also, if the dropout rate is so high that the power to show a survival benefit is minimal, then the primary endpoint of the trial will be changed to compare 12 month changes in ALSFRS-R and participants will be given the option of discontinuing study medication at 12 months. Stopping for efficacy is based upon alpha spending rules, described in Section 9.1.3.

The PI and the Steering Committee will work with the appointed DSMB to evaluate safety on an ongoing basis, and develop safety stopping rules as needed.

The DSMB will meet approximately every 6 months and at the end of the study. In addition, the DSMB will meet prior to study initiation and after completion of STAGE 1 and STAGE 2. Meetings may be conducted by teleconference at the request of the Board members. In addition, the Chairman of the Board may call ad hoc meetings. The PI will be responsible for overseeing the preparation of reports to the NINDS DSMB as well as interim communication through the program staff, should more frequency notification of safety issues be required. Summary reports of the frequency of all clinical adverse events and safety laboratory tests will be generated at pre-specified time points by the Statistical Consultant and the Coordination Center and forwarded to the DSMB for review. As necessary, the DSMB can review the frequencies of clinical and laboratory abnormalities. The DSMB will be responsible for establishing safety-stopping rules. The PI will provide some suggested safety stopping rule for consideration by the DSMB. Recommendations for modification or termination of the trial based on safety data will be made by the DSMB to the PI.

9.4.2 The Medical Monitor

The Medical Monitor will be responsible for independent review of the safety laboratory tests and adverse events. The Medical Monitor will be responsible for monitoring the real-time reporting of Serious Adverse Events, and will review laboratory reports and adverse events monthly, or more frequently as needed. He or she will be blinded to treatment assignment. If the Medical Monitor has concerns regarding the safety data, he or she may notify the DSMB.

9.5 Data Analyses

Section 9.1, above, includes detailed information regarding Data Analyses

10. DATA COLLECTION, SITE MONITORING, AND ADVERSE EXPERIENCE MONITORING

10.1 Records to be Kept and Project Organization

The MGH Neurology Clinical Trials Unit (NCTU) Coordination Center and the Biostatistics Center at the MGH will work in close collaboration with the Neurology Clinical Research Unit at SUNY Upstate Medical University to conduct the proposed study. The project coordination, data management, biostatistics, regulatory compliance, adverse event reporting, quality assurance issues and budgetary and contract components will be coordinated from MGH. The outcome measure training and study monitoring will be conducted by SUNY Upstate Medical University. This distributed effort is due to the expertise in project coordination, data management and biostatistics for multi-center clinical trials in ALS at MGH and the expertise at SUNY Upstate Medical University in outcome measure assessment and training and study monitoring. The project coordination staff will work in collaboration with the PI and Co-PIs to accomplish all the tasks necessary for preparation and implementation of the project, including the drafting of the study protocol, development of the model consent document, IND application and annual report, assisting in communications with the Steering Committee, FDA, DSMB, developing project timelines, compiling required documents from sites, preparing the detailed manual of operations, and overseeing enrollment and enrollment projections. The project coordination staff will reply

to site protocol inquiries on a daily basis, track adverse events, study drug discontinuations and study compliance.

A trial network will be set up that will utilize the Internet, telephone conference calls, and periodic mailings as its means of communications. Also, a contact information database will be created and maintained that will be used for group/individual mailing of paper documents and to facilitate telephone and fax communication between the Coordination Center and clinical sites. A Web portal has been established to provide the study personnel with an easily accessible repository of documents, which is essential for sharing information across multiple clinical trial sites. The secure, password-protected portal will contain role-based resources for study personnel. For example, the portal provides personnel lists with contact information, the complete study protocol, model informed consent forms and other study-related documents, a list of subject accrual figures that is updated continuously to allow anyone in the group to monitor study progress, a list of frequently asked questions for quick reference, and other resources that the clinical investigators and coordinators might find useful.

The Coordination Center has selected a central laboratory for the study. This laboratory has provided its current certificate of the laboratory and the curriculum vitae of the central laboratory director to the Study PI. In addition, a table listing the ranges of values considered normal for the determinations each laboratory is performing for the trial has been provided.

10.2 Role of Data Management

10.2.1 Data Flow and Management Overview

The Data Management staff at the MGH NCTU will be responsible for all aspects of data acquisition and processing, from the design of instruments used to collect data through the delivery of an accurate and timely database to the Biostatistics Center. In conjunction with the PI and the Biostatistician, the data management staff at MGH will design the electronic case report forms (eCRFs) that will capture all the data collected as part of the protocol. Interactive computer modules for real-time capture of all important study events including enrollments, serious adverse events (SAEs), reportable incidents and premature withdrawals will be created. The Electronic Data Capture and Data Management System (EDC&DM) will be tested and validated to ensure accuracy, reliability and consistent intended performance. The system conforms to the 21 CFR Part 11 and other guidance documents on computerized systems in clinical trials. The system will allow the study sites to single-enter the data via an Internet browser-based interface. The entered data will be saved in a Microsoft SQL Server database located on a server maintained by Partners Healthcare Systems IS department. Necessary documentation on the validation procedure will be maintained by the Coordination Center. A full time data manager will be responsible for the data acquisition and management at the MGH Coordination Center.

The flow of data and the data clarification process is summarized in Figure 7. The site personnel are instructed to enter information within 48 hours of the visit. An edit checking and data clarification process will be put in place to ensure accuracy and completeness of the database. Logic and range checks as well as more sophisticated rules will be built into the Web-based

forms (eCRFs) to provide immediate error checking of the data entered. The system will automatically create electronic queries on behalf of the Data Manager if saved electronic Case Report Forms (eCRFs) contain data that are out of range, out of window, missing or not calculated correctly. The Data Manager identifies the errors in the EDC system by using electronic logic checks and the Study Monitor identifies errors by direct visualization and comparison of data entered into the system with the source documents. Any inconsistent or questionable data points are queried to the sites and followed up on by both the Study Monitor and Data Manager as needed. Once the site addresses the query, the Data Manager or the Study Monitor can verify that the response is satisfactory, and that the value in the data field has been corrected. The Data Manager or Study Monitor can then "close" the query in the database. The sites and Study Monitor will only have access to the queries concerning their subjects. The Data Manager will be able to see the queries for the entire subject population. The Sites will have the ability to write a comment for a form or field. Each comment has its own history, which is recorded in a log. The log records the information that has been entered in the comment, who entered it, and when it was entered. The comment field is treated as a regular data field; hence all changes to the comments are tracked in the audit trail. We propose to use the National Cancer Institute's (NCI) Common Terminology Criteria for Coding Adverse Events (CTCAE) version 3.0. This is a descriptive terminology, organized by body system and including specific criteria for grading severity of Adverse Events. This system will allow study coordinators to quickly search for the most relevant term for each event and will give specific criteria governing the reporting of severity for each term. With this system, the event will be coded at the site and subsequently checked by the Data Manager. The system will have predefined roles and system Administrators will assign them to the system users. Depending on the role assigned, the users will differ not only in their rights to enter or view certain data, but also in their rights to access certain forms and views. These will be described fully in the EDC system functional specification document section of the Manual of Operations.

Figure 7. Data Flow and Clarification

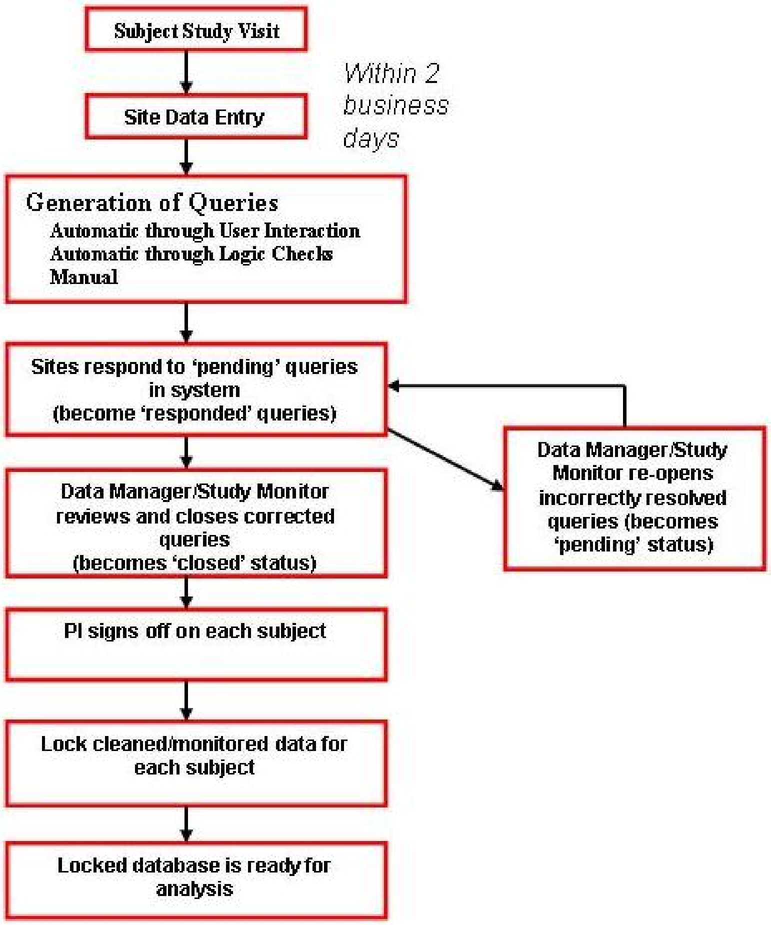

10.2.2 Database Security

The MS SQL Server database is located on a secure database server. This server is located in a restricted area of the Partners Healthcare server farm and physical access to it is limited to authorized personnel only. Both database and Web servers are located on the Partners Healthcare network behind the firewall. Access to the data at the clinical site will be restricted and monitored through the system's software with its required log-on, security procedures and audit trail. The data will not be altered, browsed, queried, or reported via external software applications that do not enter through the protective system software. There will be a cumulative record that indicates, for any point in time, the names of authorized personnel, their titles, and a description of their access privileges. The record will be in the study documentation accessible at the site. Controls will be in place to prevent, detect, and mitigate effects of computer viruses on study data and software. The application utilizes SSL (Secure Sockets Layer) technology and 128-bit encryption to comply with requirements of 21 CFR Part 11 for Open Systems. Backups of the database will be performed nightly using the services provided by the MGH network. All PC's run virus protection software full-time and are updated with the latest virus detection strings

regularly; the Windows NT server does this as well and has the additional security of scanning all e-mail for viruses before a user can even access them. All accounts are password protected and passwords must be changed on a regular basis.

In addition, the EDC system will have an extra level of password security. At study initiation, the Data Manager will set default passwords for the relevant study personnel at the MGH NCTU and at the study sites. When a new user logs in with the assigned username and default password for the first time, he or she will be forced to change the password to a unique one (at least six characters long), known only to the user. An ongoing paper log will be kept showing when usernames and passwords are set up, for whom, in what user capacity and when usernames are disabled. In case an employee forgets her/his password or a new user is added, they will submit a password request form via email to the Data Manager, who will issue a new default password. They must then go through the Change Password process. The passwords will expire every three months, when users will be required to go through the Change Password process. To avoid password-based software attacks, the system will lock a user for 1 minute if an incorrect password is provided 3 times in a row. A user will also be able to change the password at will if he or she feels that it may have been compromised.

10.2.3 Data Lock Process

The application will have the ability to lock the database to prevent any modification of data once the study is closed. Once this option is activated, every user will have Read-Only access to the data. Throughout the study, the Study Monitors will be verifying the source documents against the database. The Study Monitors will review source documents and electronic case report forms for accuracy and completeness as described in the study monitoring plan. The Data Manager can only lock the database once the following steps occur: the Site Investigator has signed off on each subject, the Study Monitor has verified the subject's data, and all queries have been resolved. The database will be transferred to the Biostatistics Center by unloading the relational MS SQL Server database to a SAS format for statistical analysis. The database will also be accessible to biostatisticians for reporting and statistical analysis during the trial.

10.3 Quality Assurance

Study Monitoring. Study monitors, supervised by the PI and Co-PIs, will visit each study site at least twice annually to review source documentation materials, informed consent forms, and confirm entered data and that queries have been accurately completed. Study Monitors will also verify that Adverse Events and protocol violations have been reported appropriately to the Coordination Center and their local IRB as required. The Study Monitor will also review clinical facilities resources and procedures for evaluating study subjects and study drug dispensing. Subsequently, the Study Monitor will provide reports of protocol compliance to PI and the Steering Committee. Completed informed consent forms from each subject must be available in the subjects file and verified for proper documentation.

10.4 Adverse Experiences Monitoring and Reporting

An adverse event (AE) is any unfavorable and unintended sign (including a clinically significant abnormal laboratory finding, for example), symptom, or disease temporally associated with a study, use of a drug product or device whether or not considered related to the drug product or device.

The adverse event (AE) definitions and reporting procedures provided in this protocol comply with current CFR 21 Part 312 and device regulations CFR 21 Part 812. The Site Investigator will carefully monitor each subject throughout the study for possible adverse events. All AEs will be documented on eCRFs designed specifically for this purpose. It is also important to report all AEs, especially those that result in permanent discontinuation of the investigational drug being studied, whether serious or non-serious.

For the purposes of this study, symptoms of progression/worsening of ALS, including 'normal' progression, will be recorded as adverse events. The following measures of disease progression will not be recorded as adverse events, even if they worsen (they are being recorded and analyzed separately): vital capacity results, HHD results, grip strength results, and ALSFRS-R ratings.

Subjects will be monitored for adverse events from the time they sign consent until completion of their participation in the study. Relationship of adverse experiences to the experimental intervention will be assessed at each in-person and telephone study visit by recording all voluntary complaints of subjects and by assessment of the clinical features of ALS. Attention will be directed to clinical adverse experiences associated with prior ceftriaxone studies, as well as any evidence of unexpected worsening of the underlying ALS. Laboratory surveillance tests will be obtained as outlined above.

A serious adverse event is defined as an adverse event that meets any of the following criteria:

- Results in death

- Is life threatening: that is, poses an immediate risk of death as the event occurred
  This serious criterion applies if the study subject, in the view of the Site Investigator, is at substantial risk of dying from the AE as it occurs. It does not apply if an AE hypothetically might have caused death if it were more severe.

- Requires inpatient hospitalization or prolongation of existing hospitalization.
  Hospitalization for an elective procedure (including elective PEG tube/g-tube/feeding tube placement) or a routinely scheduled treatment is not an SAE by this criterion because a "procedure" or a "treatment" is not an untoward medical occurrence.

- Results in persistent or significant disability or incapacity
  This serious criterion applies if the "disability" caused by the reported AE results in a substantial disruption of the subjects' ability to carry out normal life functions.

- Results in congenital anomaly or birth defect in the offspring of the subject (whether the subject is male or female)

- Necessitates medical or surgical intervention to preclude permanent impairment of a body function or permanent damage to a body structure.

An event is considered "life-threatening" if it places the subject, in the view of the Investigator, at immediate risk of death from the reaction as it occurred; i.e., it does not include a reaction that, had it occurred in a more severe form, might have caused death.

Important medical events that may not result in death, are not life-threatening, or do not require hospitalization may also be considered SAEs when, based upon appropriate medical judgment, they may jeopardize the subject and may require medical or surgical intervention to prevent one of the outcomes listed in this definition. Examples of such medical events include blood dyscrasias or convulsions that do not result in inpatient hospitalization, or the development of drug dependency or drug abuse.

An inpatient hospital admission in the absence of a precipitating, treatment-emergent, clinical adverse event may meet criteria for "seriousness" but is not an adverse experience, and will therefore, not be considered an SAE. An example of this would include a social admission (subject admitted for other reasons than medical, e.g., lives far from the hospital, has no place to sleep).

An unexpected adverse event is any adverse event, the specificity or severity of which is not consistent with the current Investigator's Brochure.

All serious adverse events (SAE) (including death and hospitalization), premature withdrawal, dosage changes and emergency treatment disclosures will be reported to the Coordination Center within one working day and SAE forms completed within 24 hours of their occurrence. The Medical Monitor and DSMB will review all SAE reports. Serious adverse events that occur during this study will be recorded in the subject's chart and on the CRF. All unexpected and related SAEs will be reported to the FDA, Health Canada as required, and to all sites by the Coordination Center. Death due to progression of disease, as expected in ALS, will not be reported in an expedited manner except in cases where the outcome of death is deemed related to study drug.

Sites will be instructed to submit these to their IRBs as well. Additionally, all SAEs will be reported to the FDA annually in the IND report. Any SAE that results in the study being put on hold at any site will be reported to all sites IRB, the NIH and the FDA. All unexpected and related SAEs will be reported to the FDA in an expedited manner, as required by FDA guidelines. Any adverse experiences will be followed for resolution.

There will be ongoing monitoring of non-serious Adverse Experiences through both the EDC system and on-site monitoring visits, to ensure adequate reporting of such events. The MGH Coordination Center will report all AEs to the FDA annually, and sites will be instructed to report AEs as required by their local IRBs. We have included in the Steering Committee two experts in infectious disease, one expert in nephrology, and one expert in gastroenterology, to advise us on how to minimize adverse events from the study drug.

10.4.1 Evaluating and Recording of Adverse Events

At each visit all adverse events that are observed, elicited by the Site Investigator, or reported by the subject will be recorded in the appropriate section of the Adverse Event log, entered into the EDC and evaluated by the Site Investigator.

Minimum information required for each AE includes type of event, duration (start and end dates), severity, seriousness, causality to study drug, action taken, and outcome.

The Site Investigator will grade the severity of AEs. The severity grading will be based on the unique clinical descriptions of severity for each AE in the CTCAE system, which follows this general guideline:

Grade 1	Mild AE
Grade 2	Moderate AE
Grade 3	Severe AE
Grade 4	Life-threatening or disabling AE
Grade 5	Death related to AE

The relationship of the AE to the study drug should be specified by the Site Investigator, using the following definitions:

1. Not Related: Concomitant illness, accident or event with no reasonable association with treatment.
2. Unlikely: The reaction has little or no temporal sequence from administration of the study drug, and/or a more likely alternative etiology exists.
3. Possibly Related: The reaction follows a reasonably temporal sequence from administration of the drug and follows a known response pattern to the suspected drug; the reaction could have been produced by the study drug or could have been produced by the volunteer's clinical state or by other modes of therapy administered to the volunteer.
4. Probably Related: The reaction follows a reasonable temporal sequence from administration of study drug; is confirmed by discontinuation of the study drug or by rechallenge; and cannot be reasonably explained by the known characteristics of the volunteer's clinical state.
5. Definitely Related: The reaction follows a reasonable temporal sequence from administration of study medication; that follows a known or expected response pattern to the study medication; and that is confirmed by improvement on stopping or reducing the dosage of the study medication, and reappearance of the reaction on repeated exposure.

If discernible at the time of completing the AE log entry, a specific disease or syndrome rather than individual associated signs and symptoms should be identified by the Site Investigator and recorded on the AE log. However, if an observed or reported sign, symptom, or clinically

significant laboratory anomaly is not considered by the Site Investigator to be a component of a specific disease or syndrome, then it should be recorded as a separate AE on the AE log. Clinically significant laboratory abnormalities are those that are identified as such by the Site Investigator and/or those that require intervention. The only exception to this will be ALS progression symptoms as previously noted.

11 HUMAN SUBJECTS

11.1 Institutional Review Board (IRB) Review and Informed Consent

The PI has obtained an IND from the FDA (IND 68,892), and has obtained approval from the Partners Human Research Committee (HRC, or Partners IRB) for the NCTU to serve as the Coordination Center for this trial. Each of the participating sites will also be required to obtain IRB approval of the protocol and consent form and send copies of these to the Coordination Center. The PI and Project Manager will review any alterations of the consent documents made by individual sites, to assure that all essential elements remain. Any subsequent modifications of the protocol or consent documents will be reviewed and approved by the MGH HRC and all site IRBs. Signed consent will be obtained from each subject. The consent form will describe the purpose of the study, the procedures to be followed, and the risks and benefits of participation. A copy of the consent form will be given to the subject, and this will be documented.

The PI and all key personnel involved in the study will have completed the Collaborative IRB training initiative, a mandatory tutorial on the responsible conduct of human subject research, or will have completed a comparable, institution-approved tutorial regarding the protection of human subjects. The NINDS has appointed a Data Safety Monitoring Board to review the study data for safety concerns. The PI will maintain minutes of the DSMB meetings and provide these to all the sites for submission to their IRBs. The PI will be responsible to report adverse events experienced by study subjects to the FDA, MGH IRB, the DSMB, NINDS and to all site investigators.

11.2 Subject Confidentiality

Confidentiality will be maintained, as all subject research data will be coded with subject ID number and initials. The Coordination Center files will be kept in a secure, double-locked area. The electronic database used during the trial will be secure. To date no breach of our security barriers has occurred, and we actively maintain a high level of security to assess the confidentiality of our databases. Only key personnel in this proposal will have access to the data and the codes. Subject results will never be discussed in any form in the presence of other subjects in the study or with non-laboratory personnel. A subject will be referred to by his/her randomization ID number only. The primary risks of participating in the study are the potential adverse effects from the experimental intervention and the central venous catheter. The PI, Steering Committee, Medical Monitor and Data Safety and Monitoring Board will monitor safety and risks throughout the study. In the rare event that an investigator needs immediate knowledge of the subject's treatment assignment, the individual treatment assignment generated by the Biostatistics Center will be available.

11.3 Inclusion of Women

The gender distribution for subjects with sporadic ALS is approximately 60% male and 40% female. The study goal is to recruit men and women with ALS in a 3:2 ratio. The MGH patient population includes 53% men and 47% women. Subjects at participating sites will be recruited from those site-specific areas and their surrounding communities. Special efforts will be employed to recruit female subjects. Advertising the study with several ALS and MND foundations will aid in the recruitment process, and in particular with the recruitment of female subjects.

The sites in the proposed study have demonstrated the ability to enroll females in prior NEALS and industry-sponsored studies. In the NEALS multi-center topiramate trial in ALS, 36% of the subjects were female. Similarly in the NEALS clinical trial of creatine in ALS and in the clinical trial of celebrex in ALS, 39% and 36% of the subjects were female, respectively. Each site has provided detailed enrollment goals and all sites documented ability to enroll 40% females. Copies of these documents are kept at the MGH Coordination Center. Based on this information, we do not anticipate difficulty enrolling the number of females for this study that represents the percentage of females with ALS in the total population.

11.4 Inclusion of Minorities

ALS is a relatively rare disease and there is mixed data on its incidence in minority groups. Most epidemiologic studies of ALS have investigated homogeneous populations. One of the only mortality studies conducted within a large, multi-ethnic population by Annegers et al. (1991) found that age- and sex-adjusted mortality did not differ among ethnic groups [191] Some studies suggest that ALS may be less frequent in non-white individuals. An incidence study in the state of Washington found rates in white males to be 1.8 per 100,000 compared to 0.74 per 100,000 per year for black males, although the difference was not statistically significant due to the small numbers of incident cases [8]. Dr. Kasarskis recently determined the rates of ALS in whites, blacks and other racial groups in a cohort of US soldiers serving during the Gulf War I era. They concluded that the rate for ALS/MND in “Other racial groups” (mainly Hispanic) was significantly elevated compared to whites in this young, predominantly male cohort whereas the overall rate for blacks was 33% lower than the specific rates estimates [192].

Approximately 17% of the national population is of a non-white racial background. For the purposes of recruitment in this study we will assume a racial distribution similar to the overall US population. Ethnically, 12.5% of the national population is Hispanic or Latino (http://www.census.gov). Thus, the study goal is to enroll at least 12.5% of the subjects from this ethnic group. Approximately 13% of our nation’s population is Black, 0.3% is Hawaiian/Pacific Islander, 4.2% is Asian, and approximately 1.5% is American Indian. Our goal is to enroll subjects with ALS that are representative of the nationwide demographics. The racial composition of MGH is 82% White, 3% Black, 1% Asian, and 12% unknown. The ethnic population of MGH is 3% Hispanic and 97% non-Hispanic. For geographical reasons, much of the local minority population does not have the MGH as its primary care facility in Boston.

To reach the recruitment goal, efforts will be focused on enrollment of individuals with ALS who are members of underrepresented minorities. Recruitment of members of minority groups will be closely monitored. Subjects will be recruited from the clinic population of the participating sites and through contact with support groups and healthcare providers from surrounding areas. IRB approval will be obtained for all planned letters, newsletters and web advertisements.

Potential study subjects will not be excluded from this study for reasons of race or gender and efforts will be made to enroll in representative numbers with respect to both gender and race. In particular, no racial discrimination will be made in subject enrollment. The participation of minority subjects will be actively encouraged throughout the study.

We will initiate different strategies to enhance minority recruitment and will include a section of these strategies in the Manual of Operations. The study PI will emphasize the significance of a balanced recruitment effort with each site investigator, as well as conduct reviews of recruitment rates. The study PI will contact sites with low minority enrollment and provide methods of improved recruitment.

11.5 Study Modification/Discontinuation

The study may be modified or discontinued at any time by the IRB (or Human Research Committee), the NINDS, the Office for Human Research Protections (OHRP), the FDA, or other government agencies as part of their duties to ensure that subjects are protected.

12 PUBLICATION OF RESEARCH FINDINGS

The Steering Committee, chaired by Dr. Cudkowicz, will be responsible for publications of results from this trial. The Steering Committee will comply with NINDS guidelines on publication of NIH funded clinical trials. Their responsibilities will include the following:

- Analyze and interpret data gathered in this study, and write publications from these data.
- Submit manuscripts to selected journals and address peer reviewers' comments.
- Submit abstracts to selected meetings and present data at the meetings.
- Determine authorship on the basis of the Uniform Requirements for Manuscripts Submitted to Biomedical Journals (International Committee of Medical Journal Editors,1997).

13 REFERENCES

1. Rothstein, J.D., et al., *Beta-lactam antibiotics offer neuroprotection by increasing glutamate transporter expression.* Nature, 2005. **433**(7021): p. 73-7.
2. Rothstein, J. and R. Kuncl, *Neuroprotective strategies in a model of chronic glutamate-mediated motor neuron toxicity.* Journal of Neurochemistry, 1995. **65**(2): p. 643-51.
3. Tikka, T., et al., *Tetracycline derivatives and ceftriaxone, a cephalosporin antibiotic, protect neurons against apoptosis induced by ionizing radiation.* Journal of Neurochemistry, 2001. **78**(6): p. 1409-14.
4. Carreer, R., et al., *Oxidant-scavenging activities of beta-lactam agents.* Eur J Clin Microbiol Infect Dis, 1998. **17**(1): p. 43-6.
5. Helms, R., et al. *Design strategies for Phases II through III: the traditional approach compared to new strategies using fewer, larger studies with multiple intermediate analyses or " non stop drug development".* in *FDA -ASA BioPharm Section workshop.* 2002: Rho, Inc. and UNC Department of Biostatistics.
6. Group, T.A.C.T.S.A.P.I.-I.S., *The amyotrophic lateral sclerosis functional rating scale.* Arch Neurol, 1996. **53**: p. 141-147.
7. Mulder, D., *Clinical limits of amyotrophic lateral sclerosis.*, in *Human motor neuron diseases.*, L. Rowland, Editor. 1982, Raven Press: New York. p. 15-22.
8. McGuire, V., et al., *Incidence of ALS in three counties in western Washington state.* Neurology, 1996. **47**: p. 571-73.
9. Mitsumoto, H., D. Chad, and E. Pioro, *Amyotrophic lateral sclerosis.* Amyotrophic Lateral Sclerosis., ed. H. Mitsumoto, D. Chad, and E. Pioro. Vol. Contemporary Neurology Series 49. 1998, New York: Oxford Press. 18-33; 164-176; 329-359.
10. Lilienfeld, D., et al., *Rising mortality from motoneuron disease in the USA:1962– 84.* Lancet, 1989: p. 710– 713.
11. Kurtzke, J. and L. Kurland, *The epidemiology of neurologic disease.*, in *Clinical Neurology*, R. Joynt, Editor. 1989, J.B. Lippincot: Philadelphia. p. 1-43.
12. Kurtzke, J.F., *Risk factors in amyotrophic lateral sclerosis.* Adv Neurol, 1991. **56**: p. 245-270.
13. Bensimon, G., L. Lacomblez, and V. Meininger, *A controlled trial of Riluzole in amyotrophic lateral sclerosis.* New England Journal of Medicine, 1994. **330**(9): p. 585-591.
14. Lacomblez, L., et al., *Dose-ranging study of riluzole in amyotrophic lateral sclerosis.* Lancet, 1996. **347**(May 25): p. 1425-1431.
15. Rosen, D.R., et al., *Mutations in Cu/Zn superoxide dismutase are associated with familial amyotrophic lateral sclerosis.* Nature, 1993. **362**: p. 59-62.
16. Andres, P.L., et al., *Use of composite scores (megascores) to measure deficit in amyotrophic lateral sclerosis.* Neurol, 1988. **38**: p. 405-408.
17. Haverkamp, L.J., V. Appel, and S.H. Appel, *Natural history of amyotrophic lateral sclerosis in a database population. Validation of a scoring system and a model for survival prediction.* Brain, 1995. **118**: p. 707-719.
18. Jablecki, C., C. Berry, and J. Leach, *Survival prediction in amyotrophic lateral sclerosis.* Muscle and Nerve, 1989. **12**: p. 833-41.
19. Gubbay, S.S., et al., *Amyotrophic lateral sclerosis: A study of its presentation and prognosis.* J Neurol, 1985. **232**: p. 295-300.

20. Eisen, A., et al., *Duration of amyotrophic lateral sclerosis is age dependent.* Muscle and Nerve, 1993. **16**: p. 27-32.
21. Rowland, L.P., *Ten central themes in a decade of ALS research*, in *Amyotrophic Lateral Sclerosis and Other Motor Neuron Diseases*, L.P. Rowland, Editor. 1992, Raven Press. p. 3-23.
22. Matzuzaki, T., et al., *HTLV-1 associated myelopathy (HAM)/tropical spastic parapareisis (TSP) with amyotrophic lateral sclerosis-like manifestations.* J Neurovirol, 2000. **6**(6): p. 544-8.
23. MacGowan, D., S. Scelsa, and M. Waldron, *An ALS-like syndrome with new HIV infection and complete response to antiretroviral therapy.* Neurology, 2001. **57**: p. 1094-1097.
24. Steele, A.J., et al., *Detection of serum reverse transcriptase activity in patients with ALS and unaffected blood relatives.* Neurology, 2005. **64**(3): p. 454-458.
25. Mu, X., et al., *Altered expression of bcl-2 and bax mRNA in amyotrophic lateral sclerosis spinal cord motor neurons.* Ann Neurol, 1996. **40**(3): p. 379-386.
26. Martin, L., *Neuronal death in amyotrophic lateral sclerosis is apoptotic: possible contribution of programmed cell death mechanism.* J Neuropathol exp Neurol, 1999. **58**: p. 459-471.
27. Rabizadeh, S., et al., *Mutations associated with amyotrophic lateral sclerosis convert superoxide dismutase from an antiapoptotic gene to a proapoptotic gene: studies in yeast and neural cells.* Proc Natl Acad Sci USA, 1995. **92**(7): p. 3024-3028.
28. Pasinelli, P., et al., *Caspase-1 and -3 are sequentially activated in motor neuron death in Cu/Zn superoxide dismutase mediated familial amyotrophic lateral sclerosis.* Proc Natl Acad Sci, 2000. **97**(25): p. 13901-13906.
29. Pasinelli, P., et al., *Caspase-1 is activated in neural cells and tissue with amyotrophic lateral sclerosis-associated mutations in copper-zinc superoxide dismutase.* Proc Natl Acad Sci, 1998. **95**: p. 15763-15768.
30. Gurney, M.E., et al., *Motor neuron degeneration in mice that express a human Cu/Zn superoxide dismutase mutation.* Science, 1994. **264**: p. 1772-1775.
31. Clement, A., et al., *Wild-type nonneuronal cells extend survival of SOD1 mutant motor neurons in ALS mice.* Science, 2003. **Oct 3**: p. 113-117.
32. Yang, Y., et al., *The gene encoding alsin, a protein with three guanine-nucleotide exchange factor domains, is mutated in a form of recessive amyotrophic lateral sclerosis.* Nat Genet, 2001. **29**: p. 160-165.
33. Hadano, S., et al., *A gene encoding a putative GTPase regulator is mutated in familial amyotrophic lateral sclerosis 2.* Nat Genet, 2001. **29**: p. 166-173.
34. Puls, I., et al., *Mutant dynactin in motor neuron disease.* Nature Genetics, 2003. **33**(4): p. 455-456.
35. Zhao, C., et al., *Charcot-Marie-Tooth disease type 2A caused by mutation in a microtubule motor KIF1Bbeta.* Cell, 2001. **105**(5): p. 587-97.
36. Nishimura AL, M.-N.M., Silva HC, Richieri-Costa A, Middleton S, Cascio D, Kok F, Oliveira JR, Gillingwater T, Webb J, Skehel P, Zatz M. , *A Mutation in the Vesicle-Trafficking Protein VAPB Causes Late-Onset Spinal Muscular Atrophy and Amyotrophic Lateral Sclerosis.* American Journal of Human Genetics, 2004. **75**(5): p. 822-831.

37. Chen, Y.-Z., et al., *DNA/RNA Helicase Gene Mutations in a Form of Juvenile Amyotrophic Lateral Sclerosis (ALS4).* American Journal of Human Genetics, 2004. **74**(6): p. 1128-1135.
38. Choi, D., *Glutamate neurotoxicity and diseases of the nervous system.* Neuron, 1988. **1**: p. 623-634.
39. Cleveland, D.W. and J.D. Rothstein, *From Charcot to Lou Gehrig: Deciphering selective motor neuron death in ALS.* Nature Reviews Neuroscience, 2001. **2**(11): p. 806-19.
40. Shaw, P. and P. Ince, *Glutamate, excitotoxicity and amyotrophic lateral sclerosis.* J Neurol, 1997. **244**(Suppl 2): p. S3-S14.
41. Urushitani, M., et al., *Mechanism of selective motor neuronal death after exposure of spinal cord to glutamate: Involvement of glutamate-induced nitric oxide in motor neuron toxicity and nonmotor neuron protection.* Ann Neurol, 1998. **44**: p. 796-807.
42. Roy, J., et al., *Glutamate potentiates the toxicity of mutant Cu/Zn-superoxide dismutase in motor neurons by postsynaptic calcium-dependent mechanisms.* J Neurosci, 1998. **18**: p. 9673-84.
43. Arriza, J., et al., *Functional comparisons of three glutamate transporter subtypes cloned from human motor cortex.* J Neurosci, 1994. **14**: p. 5559-69.
44. Fairman, W., et al., *An excitatory amino-acid transporter with properties of a ligand-gated chloride channel.* Nature, 1995. **375**: p. 599-603.
45. Lin, C., et al., *Molecular cloning and expression of the rat EAAT4 glutamate transporter subtype.* Mol Brain Res, 1998. **63**: p. 174-179.
46. Arriza, J., et al., *Excitatory amino acid transporter 5, a retinal glutamate transporter coupled to a chloride conductance.* Proc Natl Acad Sci, 1997. **94**: p. 4155-4160.
47. Chen, W., et al., *Expression of a variant form of the glutamate transporter GLT1 in neuronal cultures and in neurons and astrocytes in the rat brain.* J Neurosci, 2002. **22**: p. 2142-2152.
48. Utsunomiya-Tate, N., H. Endou, and Y. Kanai, *Tissue specific variants of glutamate transporter GLT-1.* FEBS Lett, 1997. **416**: p. 312-6.
49. Danbolt, N., *Glutamate uptake.* Prog Neurobiol, 2001. **65**: p. 1-105.
50. Danbolt, N., G. Pines, and B. Kanner, *Purification and reconstitution of the sodium and potassium-coupled glutamate transport glycoprotein from rat brain.* Biochemistry, 1990. **29**: p. 6734-6740.
51. Tanaka, K., et al., *Epilepsy and exacerbation of brain injury in mice lacking the glutamate transporter GLT-1.* Science, 1997. **276**: p. 1699-1702.
52. Rothstein, J., et al., *Knockout of glutamate transporters reveals a major role for astroglial transport in excitotoxicity and clearance of glutamate.* Neuron, 1996. **16**: p. 675-686.
53. Asztely, F., G. Erdemli, and D. Kullmann, *Extrasynaptic glutamate spillover in the hippocampus: Dependence on temperature and the role of active glutamate uptake.* Neuron, 1997. **18**: p. 281-293.
54. Bergles, D. and C. Jahr, *Synaptic activation of glutamate transporters in hippocampal astrocytes.* Neuron, 1997. **19**: p. 1297-1308.
55. Diamond, J. and C. Jahr, *Transporters buffer synaptically released glutamate on a submillisecond time scale.* J Neurosci, 1997. **17**: p. 4672-4687.
56. Barpeled, O., et al., *Distribution of glutamate transporter subtypes during human brain development.* J Neurochem, 1997. **69**: p. 2571-2580.

57. Furuta, A., et al., *Cellular and synaptic localization of the neuronal glutamate transporters excitatory amino acid transporter 3 and 4.* Neuroscience, 1997. **81**: p. 1031-1042.
58. Ginsberg, S., L. Martin, and J. Rothstein, *Regional deafferentation down-regulates subtypes of glutamate transporter proteins.* J Neurochem, 1995. **65**: p. 2800-2803.
59. Ginsberg, S., et al., *Fimbria-fornix transections selectively down-regulate subtypes of glutamate transporter and glutamate receptor proteins in suptum and hippocampus.* Neurochem, 1996. **67**: p. 1208-1216.
60. Rothstein, J., et al., *Selective loss of glial glutamate transporter GLT-1 in amyotrophic lateral sclerosis.* Ann Neurol, 1995. **38**: p. 73-84.
61. Trotti, D., et al., *SOD1 mutants linked to amyotrophic lateral sclerosis selectively inactivate a glial glutamate transporter.* Nat Neurosci, 1999. **2**: p. 427-433.
62. Howland, D., et al., *Focal loss of the glutamate transporter EAAT2 in a transgenic rat model of SOD1 mutant-mediated amyotrophic lateral sclerosis (ALS).* Proc Natl Acad Sci, 2002. **99**(3): p. 1604-9.
63. Fine, S., et al., *Tumor necrosis factor alpha inhibits glutamate uptake by primary human astrocytes. Implications for pathogenesis of HIV-1 dementia.* J Biol Chem, 1996. **271**: p. 15303-15306.
64. Su, Z., et al., *Insights into glutamate transport regulation in human astrocytes: cloning of the promoter for excitatory amino acid transporter 2 (EAAT2).* Proc Natl Acad Sci, 2003. **100**: p. 1955-1960.
65. Guo, H., et al., *Increased expression of the glial glutamate transporter EAAT2 modulates excitotoxicity and delays the onset but not the outcome of ALS in mice.* Hum Mol Genet, 2003. **12**: p. 2519-2532.
66. Sutherland, M., K. Martinowich, and J. Rothstein, *EAAT2 overexpression plays a neuroprotective role in the SOD1 G93A model of amyotrophic lateral sclerosis.* Society for Neuroscience Abstracts, 2001: p. 27.
67. Nagai, M., et al., *Rats expressing human cytosolic Cu/Zn SOD1 transgenes with amyotrophic lateral sclerosis-associated mutations develop motor neuron disease.* J Neurosci, 2001. **21**(23): p. 9246-9254.
68. Diaz, Z., et al., *Astroglial cytoprotection by erythropoietin pre-conditioning: implications for ischemic and degenerative CNS disorders.* Journal of Neurochemistry, 2005. **93**(2): p. 392-402.
69. Yogev, R., et al., *Once daily ceftriaxone for central nervous system infections and other serious pediatric infections.* Pediatric Infectious Disease, 1986. **5**: p. 298-303.
70. Congeni, B., J. Bradley, and M. Hammerschlag, *Safety and efficacy of once daily ceftriaxone for the treatment of bacterial meningitis.* Pediatric Infectious Disease, 1986. **5**(3): p. 293-297.
71. Stoeckel, K., et al., *Effects of concentration-dependent plasma protein binding on ceftriaxone kinetics.* Clin Pharm Ther, 1981. **29**: p. 650-657.
72. Patel, I. and S. Kaplan, *Pharmacokinetic profile of ceftriaxone in man.* Am J Med, 1984. **77**(Suppl 4C): p. 17-25.
73. Kroh, U., et al., *Pharmacokinetics of ceftriaxone in patients undergoing continuous veno - venous hemofiltration.* J Clin Pharmacol, 1996. **36**: p. 1114-1119.
74. Patel, I., et al., *Lack of probenecid effect on nonrenal excretion of ceftriaxone in anephric patients.* J Clin Pharmacol, 1990. **30**: p. 449-453.

75. Bergan, T., *Pharmacokinetic Properties of the cephalosporins.* Drugs, 1987. **34**(Suppl 2): p. 89-104.
76. Stoeckel, K., et al., *Effect of probenecid on the elimination and protein binding of ceftriaxone.* Eur J Clin Pharmacol, 1988. **34**: p. 151-156.
77. McNamara, P., V. Trueb, and K. Stoeckel, *Ceftriaxone binding to human serum album.* Biomedical Pharmacology, 1990. **40**(6): p. 1247-1253.
78. Lutsar, I., H. McCracken, and I. Friedland, *Antibiotic pharmacodynamics in cerebrospinal fluid.* Clinical Infectious Diseases, 1998. **27**: p. 1117-29.
79. Granero, L., et al., *Analysis of ceftriaxone and ceftazidime distribution in cerebrospinal fluid of and cerebral extracellular space in awake rats by in vivo microdialysis.* Antimicrob Agents Chemother, 1995: p. 2728-2731.
80. Spector, R., *Ceftriaxone transport through the blood brain barrier.* J Infect Dis, 1987. **156**(1): p. 209-211.
81. Kazragis, R., et al., *In vivo activities of ceftriaxone and vancomycin against Borrelia spp. in the mouse brain and other sites.* Antimicrob Agents Chemother, 1996: p. 2632-2636.
82. Marchou, B., T. Tho, and M. Armengaud, *Diffusion of ceftriaxone (Ro 13-9904/001) in the cerebrospinal fluid: Comparison with other beta-lactam antibiotics in dogs with healthy meninges and in dogs with experimental meningitis.* Chemotherapy, 1981. **27**(Suppl. 1): p. 37-41.
83. Chandrasekar, P., et al., *Diffusion of ceftriaxone into the cerebrospinal fluid of adults.* Journal of Antimicrobial Chemotherapy, 1984. **14**: p. 427-430.
84. Nau, R., et al., *Passage of cefotaxime and ceftriaxone into cerebrospinal fluid of patients with uninflammed meninges.* Antimicrob Agents Chemother, 1993. **37**(7): p. 1518-1524.
85. Chandrasekar, P., et al., *Penetration of ceftriaxone in human cerebrospinal fluid.* Clin Res, 1983. **31**: p. 628A.
86. Pfister, H., et al., *Randomized comparison of ceftriaxone and cefotaxime in Lyme neuroborreliosis.* Journal of Infectious Disease, 1991. **163**: p. 311-318.
87. Hattori, T., H. Kobayashi, and T. Uno, *Study on the penetration of ceftriaxone into cerebrospinal fluid.* Japanese Journal of Antibiotics., 1996. **49**(9): p. 813-817.
88. Clara, N., *CSF exchange after the erroneous intrathecal injection of 800 mg ceftriaxone for pneumococcal meningitis.* J Antimicrob Chemother, 1986. **17**(2): p. 263-5.
89. Pichler, H., et al. *Ceftriaxone vs piperacillin for acute bacterial meningitis in adults.* in *8th Mediterraneaon Congress of Chemotherapy*. 1988. Italy.
90. Miller, R., et al., *A placebo-controlled trial of recombinant human ciliary neurotrophic (rhCNTF) factor in amyotrophic lateral sclerosis.* Ann Neurol, 1996. **39**: p. 256-260.
91. Group, A.C.T.S., *A double-blind placebo-controlled clinical trial of subcutaneous recombinant human ciliary neurotrophic factor (rhCNTF) in amyotrophic lateral sclerosis.* Neurol, 1996. **46**: p. 1244-1249.
92. Miller, R., et al., *Placebo-controlled trial of gabapentin in patients with amyotrophic lateral sclerosis.* Neurology, 1996. **47**: p. 1383-1388.
93. Miller, R.G., et al., *Phase III randomized trial of gabapentin in patients with amyotrophic lateral sclerosis.* Neurology, 2001. **56**: p. 843-848.
94. BDNF Study Group (Phase III), *A controlled trial of recombinant methionyl human BDNF in ALS:* . Neurology, 1999. **52**(7): p. 1427-33.
95. Cudkowicz, M., et al., *A randomized, placebo-controlled trial of topiramate in amyotrophic lateral sclerosis.* Neurology, 2003. **61**(4): p. 456-464.

96. Cudkowicz, M., J. Shefner, D. Schoenfeld, J. Rothstein, D. Drachman, Northeast ALS Consortum NEALS, *Clinical Trial of Celecoxib in Subjects with Amyotrophic Lateral Sclerosis.* Amyotrophic Lateral Sclerosis and Other Motor Neuron Disorders, 2004. **5**(Supplement 2): p. 25.
97. Groenveld GJ and J. Veldink.
98. Shefner, J.M., et al., *A clinical trial of creatine in amyotrophic lateral sclerosis.* Neurology, 2004. **63**: p. 1656-1661.
99. Ripps, M.E., et al., *Transgenic mice expressing an altered murine superoxide dismutase gene provide an animal model of amyotrophic lateral sclerosis.* PNAS, 1995. **92**(3): p. 689-93.
100. Bruijn, L., et al., *ALS-linked SOD1 mutant G85R mediates damage to astrocytes and promotes rapidly progressive disease with SOD1-containing inclusions.* Neuron, 1997. **18**(2): p. 327-38.
101. Flanagan, S., et al., *Overexpression of manganese superoxide dismutase attenuates neuronal death in human cells expressing mutant (G37R) Cu/Zn-superoxide dismutase.* J. Neurochem, 2002. **81**: p. 170-177.
102. Ghadge, G., et al., *Mutant superoxide dismutase-1-linked familial amyotrophic lateral sclerosis: molecular mechanisms of neuronal death and protection.* J Neuroscience, 1997. **17**(22): p. 8756-66.
103. Leikis, M.J., et al., *Haemoglobin response to subcutaneous versus intravenous epoetin alfa administration in iron-replete haemodialysis patients.* Nephrology, 2004. **9**(3): p. 153-160.
104. Martinez-Rodriguez, J., et al., *Nonconvulsive status epilepticus associated with cephalosporins in patients with renal failure.* The American Journal of Medicine, 2001. **111**: p. 115-119.
105. Norrby, R., *Side effects of cephalosporins.* Drugs, 1987. **34**(Suppl 2): p. 105-120.
106. Tierney, L., et al., *Current Medical Diagnosis and Treatment*, ed. McGraw-Hill. 2002.
107. Massry, S.G. and R.J. Glassock, eds. *Massry and Glassock's Textbook of Nephrology*. 4 ed. 2001, Lippincott Williams and Wilkins: Philadelphia. 2072.
108. Dattwyler, R., J. Halperin, and H. Pass, *Ceftriaxone as effective therapy in refractory Lyme disease.* J Infect Dis, 1987. **155**: p. 1322-5.
109. Suttorp-Schulten, M., et al., *Long-term effects of ceftriaxone treatment on intraocular lyme borreliosis.* American Journal of Ophthalmology, 1993. **116**: p. 571-575.
110. Dattwyler, R., J. Halperin, and D. Volkman, *Treatment of late Lyme borreliosis-randomized comparison of ceftriaxone and penicillin.* Lancet, 1988. **1**: p. 1191-4.
111. Green, S., *Practical guidelines for developing an office-based program for outpatient intravenous therapy.* Reviews of Infectious Diseases, 1991. **13**(Suppl 2): p. S189-92.
112. Wiselka, M. and K. Nicholson, *Outpatient parenteral antimicrobial therapy: Experience in a large teaching hospital.* Journal of Infection, 1997. **35**(1): p. 73-76.
113. Lawrence, C., et al., *Seronegative chronic relapsing neuroborreliosis.* European Neurology, 1995. **35**: p. 113-117.
114. Workman, M., et al., *Emergence of ciprofloxacin resistance during treatment of salmonella osteomyelitis in three patients with sickle cell disease.* Journal of Infection, 1996. **32**: p. 27-32.
115. Eron, L., C. Park, and D. Hixon, *Ceftriaxone therapy of bone and soft tissue infections in hospital and outpatient settings.* Antimicrob Agents Chemother, 1983. **23**: p. 731-7.

116. Eron, L., R. Goldenberg, and D. Poretz, *Combined ceftriaxone and surgical therapy for osteomyelitis in hospital and outpatient settings.* Am J Surg, 1984. **148**(Suppl 4A): p. 1-4.
117. Guglielmo, B., et al., *Ceftriaxone therapy for staphylococcal osteomyelitis: a review.* Clinical Infectious Diseases, 2000. **30**(1): p. 205-7.
118. Breier, F., et al., *Isolation and polymerase chain reaction typing of Borrelia afzelii from a skin lesion in a seronegative patient with generalized ulcerating bullous lichen sclerosus et atrophicus.* British Journal of Dermatology, 2001. **144**: p. 387-392.
119. Meyers, B., et al., *Crossover study of the pharmacokinetics of ceftriaxone administered intravenously or intramuscularly to healthy volunteers.* Antimicrob Agents Chemother, 1983: p. 812-814.
120. Merriman, T., *A 6-month toxicity study of ceftriaxone administered by subcutaneous injection to rats with a 1-month recovery period. Study No. AAK00010.* 2008.
121. Mermel, L.A., et al., *Guidelines for the management of intravascular catheter-related infections.* Clin Infect Dis, 2001. **32**(9): p. 1249-72.
122. Moureau, N., et al., *Central venous catheters in home infusion care: outcomes analysis in 50,470 patients.* J Vasc Interv Radiol, 2002. **13**(10): p. 1009-16.
123. O'Grady, N., et al., *Guidelines for the prevention of intravascular catheter-related infections.* Am J Infect Control, 2002. **30**: p. 476-89.
124. Safdar, N., D. Kluger, and D. Maki, *A review of risk factors for catheter-related bloodstream infection caused by percutaneously inserted, noncuffed central venous catheters.* Medicine, 2002. **81**: p. 466-79.
125. Maki, D., C. Weise, and H. Sarafin, *A semiquantitative culture method for identifying intravenous-catheter related infection.* N Engl J Med, 1977. **296**: p. 1305-9.
126. Mermel, L.A., et al., *The pathogenesis and epidemiology of catheter-related infection with pulmonary artery Swan-Ganz catheters: a prospective study utilizing molecular subtyping.* Am J Med, 1991. **91**(3B): p. 197S-205S.
127. Sitges-Serra, A., et al., *A randomized trial on the effect of tubing changes on hub contamination and catheter sepsis during parenteral nutrition.* JPEN J Parenter Enteral Nutr, 1985. **9**(3): p. 322-5.
128. Linares, J., et al., *Pathogenesis of catheter sepsis: a prospective study with quantitative and semiquantitative cultures of catheter hub and segments.* J Clin Microbiol, 1985. **21**(3): p. 357-60.
129. Raad, I., et al., *Ultrastructural analysis of indwelling vascular catheters: a quantitative relationship between luminal colonization and duration of placement.* J Infect Dis, 1993. **168**(2): p. 400-7.
130. Hyman, G.S. and D.D. Cardenas, *Upper-extremity deep vein thrombosis associated with peripherally inserted central catheters in acute spinal cord injury: a report of 2 cases.* Arch Phys Med Rehabil, 2002. **83**(9): p. 1313-6.
131. Tseng, M., et al., *Radiologic placement of central venous catheters: rates of success and immediate complications in 3412 cases.* Can Assoc Radiol J, 2001. **52**(6): p. 379-84.
132. Eastman, M., et al., *Central Venous device-related infection and thrombosis in patients treated with moderate dose continous-infusion interleukin-2.* Cancer, 2001. **91**: p. 806-14.
133. De Cicco, M., et al., *Central venous thrombosis: An early and frequent complication in catheter patients bearing long-term silastic catheter. A prospective study.* Thrombosis Research, 1997. **86**: p. 101-103.

134. Davis, S.N., et al., *Activity and dosage of alteplase dilution for clearing occlusions of venous-access devices.* Am J Health Syst Pharm, 2000. **57**(11): p. 1039-45.
135. Gould, J.R., H.W. Carloss, and W.L. Skinner, *Groshong catheter-associated subclavian venous thrombosis.* Am J Med, 1993. **95**(4): p. 419-23.
136. Stirling, D.P., et al., *Minocycline Treatment Reduces Delayed Oligodendrocyte Death, Attenuates Axonal Dieback, and Improves Functional Outcome after Spinal Cord Injury.* J. Neurosci., 2004. **24**(9): p. 2182-2190.
137. Vrazas, J.I., *Central venous catheter placement.* Hosp Med, 1999. **60**(5): p. 337-42.
138. Klempner, M., et al., *Two controlled trials of antibiotic treatment in patient with persistent symptoms and a history of lyme disease.* N Engl J Med, 2001. **345**(2): p. 85-92.
139. Byar, D., et al., *Design considerations for AIDS trials.* New England Journal of Medicine, 1990. **323**(19): p. 1343-8.
140. Prentice, R., *Surrogate endpoints in clinical trials: Definition and operational criteria.* Statistics in Medicine, 1989. **8**: p. 431-440.
141. Dafni, U. and A. Tsiatis, *Evaluating surrogate markers of clinical outcome when measured with error.* Biometrics, 1998. **54**: p. 1445-1462.
142. Brooks, B., *El Escorial World Federation of Neurology criteria for the diagnosis of amyotrophic lateral sclerosis. Subcommittee on Motor Neuron Diseases/Amyotrophic Lateral Sclerosis of the World Federation of Neurology Research Group on Neuromuscular Diseases and the El Escorial "Clinical limits of amyotrophic lateral sclerosis".* J Neurol Sci, 1994. **124**: p. 96-107.
143. Cudkowicz, M., et al., *Longitudinal analysis of medication utilization for ALS/MND: Findings from the ALS patient care database.* Amyotrophic lateral sclerosis, 2000. **1**(Suppl 3): p. 71-72.
144. Trissel, L., *Handbook on Injectable Drugs*. 12 ed. 2003: American Society of Health System Pharmacists. 289.
145. Buke, A., et al., *Does dexamethasone affect ceftriaxone penetration into cerebrospinal fluid in adult bacterial meningitis.* International Journal of Antimicrobial Agents, 2003. **21**: p. 452-456.
146. Owens, H., C. Destaceh, and A. Dash, *Simple liquid chromatographic method for the analysis of the blood brain barrier permeability characteristics of ceftriaxone in an experimental rabbit meningitis model.* J Chromatogr B Biomed Sci Appl, 1994. **728**: p. 97-105.
147. Jungbluth, G. and W. Jusko, *Ion-paired reversed-phase high-performance liquid chromatopraphy assay for determination of ceftriaxone in human plasma and urine.* J Pharm Sci, 1989. **78**(11): p. 968-70.
148. Vonesh, E.F., T. Greene, and M.D. Schluchter, *Shared parameter models for the joint analysis of longitudinal data and event times.* Stat Med, 2006. **25**(1): p. 143-63.
149. Cedarbaum, J., et al., *The ALSFRS-R: a revised ALS functional rating scale that incorporated assessments of respiratory function.* J Neurol Sci, 1999. **169**: p. 13-21.
150. Cedarbaum, J.M. and N. Stambler, *Disease status and use of ventilatory support by ALS patients.* Amyotrophic Lateral Sclerosis & Other Motor Neuron Disorders, 2001. **2**(1): p. 19-22.
151. Clarke, S., et al., *Assessing individual quality of life in amyotrophic lateral sclerosis.* Qual Life Res, 2001. **10**(2): p. 149-58.

152. Miano, B., et al., *Inter-evaluator reliability of the ALS Functional Rating Scale.* Amyotrophic Lateral Sclerosis & Other Motor Neuron Disorders, 2004. **5**(4): p. 235-239.
153. Kasarskis, E.J., L. Dempsey-Hall, L.C. Luu, M. Mendionodo, R. Kryscio, *Rating the Severity of ALS by Caregivers over the phone using the ALSFRS-R.* ALS and Other Motor Neuron Disorders, 2004. **5**(Supplement 2): p. 12.
154. Miller, R.G., et al., *Practice parameter: the care of the patient with amyotrophic lateral sclerosis (an evidence-based review): report of the Quality Standards Subcommittee of the American Academy of Neurology: ALS Practice Parameters Task Force.* Neurology, 1999. **52**(7): p. 1311-23.
155. Albert, S.M., et al., *A prospective study of preferences and actual treatment choices in ALS.* Neurology, 1999. **53**(2): p. 278-83.
156. Albert, S.M., et al., *Prospective study of palliative care in ALS: choice, timing, outcomes.* J Neurol Sci, 1999. **169**(1-2): p. 108-13.
157. Miller, R., et al., *Practice parameter: the care of the patient with amyotrophic lateral sclerosis (an evidence-based review): report of the quality standards subcommittee of the American Academy of Neurology: ALS Practice Parameters Task Force.* Neurology, 1999. **52**: p. 1311-1323.
158. Mitsumoto, H., et al., *Percutaneous endoscopic gastrostomy (PEG) in patients with ALS and bulbar dysfunction.* Amyotroph Lateral Scler Other Motor Neuron Disord, 2003. **4**(3): p. 177-85.
159. Eisen, A. and M. Weber, *Treatment of amyotrophic lateral sclerosis.* Drugs Aging, 1999. **14**(3): p. 173-96.
160. Kleopa, K.A., et al., *Bipap improves survival and rate of pulmonary function decline in patients with ALS.* J Neurol Sci, 1999. **164**(1): p. 82-8.
161. Pinto, A., et al., *Respiratory assistance with a non-invasive ventilator (BIPAP) in MND/ALS patients: survival rates in a controlled trial.* J Neurol Sci, 1995. **129**(Suppl): p. 19-26.
162. Nelson, R., et al., *Behavioral abnormalities in male mice lacking neuronal nitric oxide synthase.* Nature, 1995. **378**(Nov 23): p. 383-386.
163. Thijs, V., et al., *Demographic characteristics and prognosis in a Flemish amyotrophic lateral sclerosis population.* Acta Neurologica Belgica, 2000. **100**(2): p. 84-90.
164. Nelson, L.M., et al., *Population-based case-control study of amyotrophic lateral sclerosis in western Washington State. II. Diet.* Am J Epidemiol, 2000. **151**(2): p. 164-73.
165. Mazzini, L., et al., *The natural history and the effects of gabapentin in amyotrophic lateral sclerosis.* J Neurol Sci, 1998. **160**: p. S57-63.
166. Slaugenhaupt, S.A., et al., *The human gene for neurotrophic tyrosine kinase receptor type 2 (NTRK2) is located on chromosome 9 but it not the familial dysautonomia gene.* Genomics, 1995. **25**: p. 730-732.
167. Pestronk, A., et al., *Distal lower motor neuron syndrome with high-titer serum IgM anti-GM1 antibodies: Improvement following immunotherapy with monthly plasma exchange and intravenous cyclophosphamide.* Neurology, 1994. **44**: p. 2027-2031.
168. Schneider-Gold, C., et al., *Creatine monohydrate in DM2/PROMM: a double-blind placebo-controlled clinical study. Proximal myotonic myopathy.* Neurology, 2003. **60**(3): p. 500-2.
169. Drachman, D., et al., *Trial of immunosuppression in amyotrophic lateral sclerosis using total lymphoid irradiation.* Ann Neurol, 1994. **35**(2): p. 142-50.

170. Beck, M., et al., *Comparison of maximal voluntary isometric contraction and Drachman's hand-held dynamometry in evaluating patients with amyotrophic lateral sclerosis.* Muscle Nerve, 1999. **22**(9): p. 1265-70.
171. Nahata, M., V. Kohlbrenner, and W. Barson, *Pharmacokinetics and cerebrospinal fluid concentrations of cefixime in infants and young children.* Chemotherapy, 1993. **39**(1): p. 1-5.
172. Ko, C.W., J.H. Sekijima, and S.P. Lee, *Biliary sludge.* Ann Intern Med, 1999. **130**(4 Pt 1): p. 301-11.
173. Cohen, J. and W. Powderly, eds. *Infectious Diseases*. 2 ed. 2004, Mosby: Edinburgh.
174. Armon, C., et al., *Linear estimates of disease progression predict survival in patients with amyotrophic lateral sclerosis.* Muscle and Nerve, 2000. **23**(6): p. 874-882.
175. Famularo, G., S. Polchi, and C. De Simone, *Acute cholecystitis and pancreatitis in a patient with biliary sludge associated with the use of ceftriaxone: a rare but potentially severe complication.* Annali Italiani di Medicina Interna, 1999. **14**(3): p. 202-4.
176. Maranan, M., S. Gerber, and G. Miller, *Gallstone pancreatitis caused by ceftriaxone.* Pediatric Infectious Disease Journal, 1998. **17**(7): p. 662-3.
177. Zimmermann, A., et al., *Ceftriaxone-induced acute pancreatitis.* Annals of Pharmacotherapy, 1993. **27**(1): p. 36-7.
178. Lopez, A., et al., *Ceftriaxone-induced cholelithiasis.* Annals of Internal Medicine, 1991. **115**(9): p. 712-4.
179. Department of Medicine, W.U.S.o.M., ed. *The Washington Manual of Medical Therapeutics*. 29 ed. 1998, Lipincott-Raven: Philadelpha.
180. Axcan, *Ursodiol Tablets, USP (Urso 250, Urso Forte) Package Insert.* Axcan Scandipharm, Inc. Birmingham, AL, 2008.
181. Monreal, M., et al., *Pulmonary embolism in patients with upper extremity DVT associated to venous central lines--a prospective study.* Thromb Haemost, 1994. **72**(4): p. 548-50.
182. Dedrick, R., et al., *Pharmacokinetic considerations on resistance to anticancer drugs.* Cancer Chemother Rep, 1975. **59**(4): p. 795-804.
183. Lindstrom, M. and D. Bates, *Nonlinear mixed-effects models for repeated measures data.* Biometrics 46, 1990. **46**: p. 673-687.
184. Lunn, D., et al., *WinBUGS -- a Bayesian modeling framework: concepts, structure, and extensibility.* Statistics and Computing, 2000. **10**: p. 325-337.
185. Greenblatt, D., et al., *Kinetics and dynamics of lorazepam during and after continuous intravenous infusion.* Crit Care Med, 2000. **8**: p. 2750-7.
186. O'Brien, P.C., *The appropriateness of analysis of variance and multiple-comparison procedures.* Biometrics, 1983. **39**(3): p. 787-94.
187. Proschan MA, L.K.a.W.J., *Statistical Monitoring of Clinical Trials: A Unified Approach*. 1 ed. 2006: Springer-Verlag New York, LLC. 105-111.
188. Robins, J. and D. Finkelstein, *Correcting for noncompliance and dependent censoring in an AIDS clinical trial with inverse probability of censoring weighted (IPCW) log-rank tests.* Biometrics, 2000. **56**(3): p. 779-788.
189. Laird, N. and J. Ware, *Random-effects models for longitudinal data.* Biometrics, 1982. **38**: p. 963-974.
190. Shah, A., D. Schoenfeld, and V. De Gruttola, *Risk estimation using a surrogate marker measured with error (STMA V37 3666), Statistical Modelling.*, in *Proceedings of the 10th*

International Workshop on Statistical Modelling. Lecture Notes in Statistics. 1995. p. 267-275.

191. Annegers, J., et al., *Incidence and prevalence of amyotrophic lateral sclerosis in Harris County, Texas, 1985-1988.* Arch Neurol, 1991. **48**: p. 589-593.

192. Kasarskis, E., et al., *Evaluation of ALS rates in different racial groups: Data from the ALS Gulf War study*. Neurology, 2003. **A140**(Suppl 1): p. A140.

Protocol Appendix 1

- Cerebrospinal Fluid Levels
- Ceftriaxone Package Insert
- Multivitamin Infusion (MVI) Package Insert

TABLE 1

CSF LEVELS IN ADULTS WITH NON-INFLAMED MENINGES

Reference	N	Dose (g)	Single/Multiple Dose	Time After 1st Dose	CSF (µg/ml)	Serum C_{max} (µg/ml)	CSF Penetration (%)	CSF T_{max} (hrs)	CSF (T ½ hr)
(1)	7	2	Single	Multiple	0.18-1.04	259.5	0.007 AUC ratio (CSF/Serum)	12 (1-16 range)	16.8 (median)
(2,3)	5<br>2<br>2<br>3	2<br>2<br>2<br>2	Single<br>Multiple (3 days)<br>Single<br>Multiple (3 days)	2<br>2<br>4<br>4	2.1<br>2.2<br>2.4<br>2.6	262.4<br>263<br>306.5<br>259.5	1.5		
(4)	17	2	Multiple (2-4 days)	3.5	2.3	134.5	1.7		
(5)	14<br><br><br>14	2<br><br><br>2	Single<br><br><br>Multiple (3-7 days)	0.5<br>3<br>24	1.7 ± 1.8<br>3.5 ± 3.3<br>0.7 ± 0.7	162 ±<br>51.2		3	

TABLE 2

CSF LEVELS IN ADULTS WITH INFLAMED MENINGES

Reference	N	Dose (g)	Single/Multiple Dose	Time After 1st Dose (hrs)	CSF (µg/ml)	Serum C_{max} (µg/ml)	CSF Penetration (%)
(6)	6	4 (2g bid)	Multiple (10 days)	24<br>48<br>72<br>96<br>264	4.85<br>3.76<br>3.92<br>4.76<br>2.90		11<br>8<br>9<br>11<br>7
(3)	3	2	Single	2-4	1.7-2.4	185-225	2.7
(2)	3	2	Single	2-4	2.03		2.2
(7)	15	75 (mg/kg)	Single	11.4-12.8	1.1-8.0	150-484	0.03-0.25
(8)	30	50-75 (mg/kg)	Multiple		5.4-6.4	230-295	
(9)	7	4 g (qd)	Multiple	3-5<br>18-24	27.8<br>7.5	128<br>51.3	

References for Tables 1 and 2:

1. Nau R, Prange H, Muth P, Mahr G, Mench S, Kolenda H, et al. Passage of cefotaxime and ceftriaxone into cerebrospinal fluid of patients with uninflamed meninges. Antimicrob Agents Chemother 1993; 37 (7): 1518-1524.

2. Chandrasekar P, Rolston K, Smith B, LeFrock J. Diffusion of ceftriaxone into the cerebrospinal fluid of adults. Journal of Antimicrobial Chemotherapy 1984; 14:427-430.

3. Chandrasekar P, Rolston K, Smith B, LeFrock J. Penetration of ceftriaxone in human cerebrospinal fluid. Clin Res 1983; 31:628A.

4. Pfister H, Mursic V, Wilske B, Schielke E, Sorgel F, Einhaupl M. Randomized comparison of ceftriaxone and cefotaxime in lyne neuroborreliosis. Journal of Infectious Disease 1991; 163: 311-318.

5. Hattori T, Kobayashi H, Uno T. Study on the penetration of ceftriaxone into cerebrospinal fluid. Japanese Journal of Antibiotics. 1996; 49(9): 813-817.

6. Buke A, Cavusoglu C, Karasulu E, Karakartal G. Does dexamethasone affect ceftriaxone penetration into cerebrospinal fluid in adult bacterial meningitis. International Journal of Antimicrobial Agents. 2003;21: 452-456.

7. Nahata M, Kohlbrenner V, Barson W. Pharmacokinetics and cerebrospinal fluid concentrations of cefixime in infants and young children. Chemotherapy 1993; 39(1): 1-5.

8. Steele R. Ceftriaxone therapy of meningitis and serious infections. The American Journal of Medicine. 1984: 50-53.

9. Pichler H, Diridi G, Jeschko E, Wolf D. Ceftriaxone vs. piperacillin for acute bacterial meningitis in adults. In: 8th Mediterraneaon Congress of Chemotherapy. 1988; Italy, 1988.

Protocol Appendix 2

El Escorial
World Federation Of Neurology
Criteria for the Diagnosis of ALS

ELSEVIER

Journal of the Neurological Sciences 124 (Suppl.) (1994) 96—107

JOURNAL OF THE
NEUROLOGICAL
SCIENCES

World Federation of Neurology Research Group on Neuromuscular Diseases

El Escorial
World Federation of Neurology Criteria for the Diagnosis of Amyotrophic Lateral Sclerosis

Subcommittee on Motor Neuron Diseases/Amyotrophic Lateral Sclerosis of the World Federation of Neurology Research Group on Neuromuscular Diseases and the El Escorial "Clinical Limits of Amyotrophic Lateral Sclerosis" Workshop Contributors

Introduction

Amyotrophic lateral sclerosis (ALS) is a progressive neurodegenerative disorder involving primarily motor neurons in the cerebral cortex, brainstem and spinal cord. The wide variety of clinical features which may be present early in the course of ALS makes absolute diagnosis difficult and compromises the certainty of diagnosis for clinical research purposes and therapeutic trials.

Because clinicians and scientists worldwide are in need of precise diagnostic criteria to apply in order for their studies to proceed, a three day workshop on "The Clinical Limits of ALS" was convened at El Escorial, Spain on May 29–31, 1990, by the World Federation of Neurology Subcommittee on Motor Neuron Disease (Amyotrophic Lateral Sclerosis). The purpose of the workshop which was funded by the Spanish Social Security Fund for Health Research (FIS) and the Spanish ALS Association (ADELA) was to develop diagnostic criteria for amyotrophic lateral sclerosis which are workable, internationally acceptable and provide an algorithm which will enhance clinical studies, therapeutic trials and molecular genetic research studies.

The draft criteria were distributed to neuromuscular disease researchers prior to open discussion of these criteria at the meeting of the World Federation of Neurology Subcommittee on Motor Neuron Disease (Amyotrophic Lateral Sclerosis) and members of the Research Group on Neuromuscular Diseases at the VIIth International Congress on Neuromuscular Diseases in Munich, Germany, on September 19, 1990. The consensus criteria were submitted to the Executive Committee of the World Federation of Neurology Research Group on Neuromuscular Diseases for review and approval.

This consensus document, reviewed, amended and ultimately accepted by all participants, and many additional clinicians and researchers involved in ALS research, as well as appropriate scientific review bodies and concerned voluntary organizations, defines the criteria for determining the diagnostic certainty of ALS that are suitable for clinical and research studies of the disease.

Benjamin Rix Brooks, MD
ALS Clinical Research Center
University of Wisconsin Hospital and Clinics
Clinical Science Center J6-504
Madison, WI 53792-5132, USA
Tel.: (608) 263-9057
Fax: (608) 263-0412

0022-510X/94/$07.00 © 1994 Elsevier Science B.V. All rights reserved
SSDI 0022-510X(94)00120-D

Criteria for the diagnosis of amyotrophic lateral sclerosis

The diagnosis of ALS ***requires*** the ***presence*** of
(1) signs of lower motor neuron (LMN) degeneration by clinical, electrophysiological or neuropathologic examination,
(2) signs of upper motor neuron (UMN) degeneration by clinical examination, and
(3) progressive spread of signs within a region or to other regions,

together with the ***absence*** of
(1) electrophysiological evidence of other disease processes that might explain the signs of LMN and/or UMN degeneration, and
(2) neuroimaging evidence of other disease processes that might explain the observed clinical and electrophysiological signs.

Steps in the diagnosis of amyotrophic lateral sclerosis

The diagnosis of ALS is made possible by
(1) history, physical and appropriate neurological examinations to ascertain clinical findings which may suggest **suspected, possible, probable** or **definite ALS**,
(2) electrophysiological examinations to ascertain findings which confirm LMN degeneration in clinically involved regions, identify LMN degeneration in clinically uninvolved regions and exclude other disorders,
(3) neuroimaging examinations to ascertain findings which may exclude other disease processes,
(4) clinical laboratory examinations, determined by clinical judgement, to ascertain possible ALS-related syndromes,
(5) neuropathologic examinations, where appropriate, to ascertain findings which may ***confirm*** or ***exclude*** **sporadic ALS, coexistent sporadic ALS, ALS-related syndromes** or **ALS variants,**
(6) repetition of clinical and electrophysiological examinations at least six months apart to ascertain evidence of progression.

Clinical features in the diagnosis of ALS

Patients with signs of LMN degeneration (weakness, atrophy and clinical fasciculations) and UMN degeneration (spasticity, pathologic reflexes, etc.) may be suspected as having ALS. Careful history, physical and neurological examination must search for further clinical evidence of LMN and UMN signs in four regions of the central nervous system (Table 1).
Clinical features ***required*** for the diagnosis of ALS
(1) *Signs of LMN degeneration (weakness, wasting and fasciculation) in one or more of the four regions (bulbar, cervical, thoracic, lumbosacral).* LMN findings in a region are without regard to right or left, but are indicative of the level of neuraxis involved. Therefore, spread of weakness, wasting and fasciculations to another region is more important than spread from right to left or vice-versa.
(2) *Signs of UMN degeneration (increased or clonic tendon reflexes, spasticity,* pseudobulbar features, Hoffmann reflex and extensor plantar response) in one or more of the four regions. These UMN signs

Table 1
Lower motor neuron and upper motor neuron signs in four regions

	Bulbar	Cervical	Thoracic	Lumbosacral
**Lower motor neuron signs** weakness, atrophy, fasciculations	jaw, face, palate, tongue, larynx	neck, arm, hand, diaphragm	back, abdomen	back, abdomen, leg, foot
**Upper motor neuron signs** pathologic spread of reflexes, clonus, etc.	clonic jaw, gag reflex, exaggerated snout reflex, pseudobulbar features, forced yawning pathologic DTRs spastic tone	clonic DTRs, Hoffmann response, pathologic DTRs, spastic tone	loss of superficial abdominal reflexes, pathologic DTRs, spastic tone	clonic DTRs, extensor plantar response, pathologic DTRs, spastic tone

are clinically appreciated best in the bulbar, cervical and lumbosacral regions. UMN findings in a region are also without regard to right or left (Table 2).

Once the physical and neurological examinations provide information on the presence or absence of LMN and UMN signs in the four regions (bulbar, cervical, thoracic, lumbosacral) they must be ordered topographically in a manner to determine the certainty of the diagnosis of ALS.

(3) *The topographical location of certain UMN and LMN signs in four regions of the CNS together with progression of these signs determines the certainty of the diagnosis of ALS.* Progression is a cardinal feature of the clinical diagnosis of ALS. Progression of signs within a region and progression of signs to involve other regions are crucial to the diagnosis.

Clinical examinations should be repeated at least every six (6) months to assess progression.

Cases which meet the topographical criteria for probable or definite ALS but which lack progression during the twelve (12) month period from diagnosis should also be designated as possible ALS.

Definite ALS

is defined on clinical grounds alone by the presence of UMN as well as LMN signs in the bulbar region and at least two of the other spinal regions or the presence of UMN and LMN signs in three spinal regions. The important determinants of the diagnosis of definite ALS in the absence of electrophysiological, neuroimaging and laboratory examinations are the presence of UMN and LMN signs together in multiple regions.

Probable ALS

is defined on clinical grounds alone by UMN and LMN signs in at least two regions. While the regions may be different, some UMN signs must be rostral (above) the LMN signs. Multiple different combinations of UMN and LMN signs may be present in patients with probable ALS.

Possible ALS

is defined on clinical grounds alone when the UMN and LMN signs are in only one region or UMN signs alone are present in 2 or more regions or LMN signs are rostral to UMN signs (the latter distribution of signs needs to be differentiated from multiple non-ALS processes). Monomelic ALS, progressive bulbar palsy without spinal UMN and/or LMN signs and progressive primary lateral sclerosis without spinal LMN signs constitute special cases which may develop LMN or UMN signs to meet the criteria for probable ALS with time or be subsequently confirmed at autopsy by specific LMN and UMN neuropathologic findings.

Suspected ALS

will manifest only LMN signs in 2 or more regions, although UMN pathology might be demonstrated at autopsy. However, only clinical signs are considered pertinent to this classification at the time of diagnostic evaluation.

Supportive clinical features

Clinical features that ***support*** the diagnosis of ALS include one or more of the following:

(1) abnormal pulmonary function tests not explained by other causes,
(2) abnormal speech studies not explained by other causes,
(3) abnormal swallowing studies not explained by other causes,
(4) abnormal larynx function studies not explained by other causes,
(5) abnormal isokinetic or isometric strength tests in clinically uninvolved muscles,
(6) abnormal muscle biopsy with evidence of denervation.

Inconsistent clinical features

Clinical findings ***inconsistent*** with the diagnosis of ALS include one or more of the following not explained by physiological changes associated with aging or other disease processes:

(1) sensory dysfunction,
(2) sphincter abnormalities,
(3) autonomic nervous system dysfunction,
(4) anterior visual pathway abnormalities,
(5) movement abnormalities associated with probable Parkinson's disease defined by DATATOP criteria,
(6) cognitive abnormalities associated with clinical Alzheimer's disease as defined by NINCDS-ADRDA criteria.

If these clinical findings occur, then close attention should be paid to the possible diagnosis of other disease processes.

Lower motor neuron and upper motor neuron signs may occur together with other clinical signs in diseases where the pathologic process is not primary motor neuron degeneration.

Types of ALS

The clinical signs of progressive LMN and UMN degeneration seen in ALS may

(a) occur alone (**sporadic ALS**),
(b) be present incidentally with other preexisting disease processes that have not developed in parallel with the ALS (**coexistent sporadic ALS**),
(c) occur in association with laboratory-defined or epidemiologically defined abnormalities that are

Table 2
Glossary

**Definite**	specific clinical and exclusionary criteria met; no other diagnosis possible on basis of clinical distribution or laboratory findings
**Dementia**	progressive deterioration of specific cognitive functions
**Extrapyramidal**	clinical features localizable to basal ganglia and/or midline cerebellum
**Hyperreflexia**	spread of deep tendon reflex outside stimulated territory
**Minor**	subjective and objective complaints confirmed by examination (utilization of instrumental sensory testing may increase the detection of sensory abnormalities)
**Onset**	time of first subjective symptom noticed by patient which later is confirmed by examination
**Possible**	specific clinical and exclusionary criteria met
**Probable**	specific clinical and exclusionary criteria met
**Radicular**	distribution conforming to particular nerve root
**Region**	brainstem, cervical, thoracic or lumbosacral spinal cord levels (regional involvement is defined by either right or left sided signs)
**Required**	necessary or sufficient
**Segment**	single brainstem or spinal cord level
**Spread**	involvement of new anatomic segments or regions in the central nervous system
**Support**	neither necessary nor sufficient, but may suggest
**Systemic**	non-central nervous system
**Weakness**	decreased isometric strength
**Worsening**	increased weakness of muscles in previously affected segment

time-linked to the ALS (**ALS-related syndromes**) or

(d) occur in association with clinical, genetic or epidemiological features which develop in parallel with the ALS (**ALS variants**).

The physical and neurological examinations will allow for the clinical diagnosis of ALS to a particular degree of certainty as defined above; however, the history of the disease onset, toxic exposures, past medical history, injuries, family history, geographic location, etc., must be incorporated with the clinical examinations in determining whether the patient may have an ALS related syndrome or an ALS variant.

ALS-related syndromes

must meet the clinical, electrophysiological and neuroimaging criteria for possible, probable or definite ALS. ALS-related syndromes have unique laboratory-defined or epidemiologically defined features which are time-liked to the development of the ALS phenotype. If correction of the associated laboratory-defined feature does not result in correction of the ALS phenotype, then the patient with an ALS-related syndrome should be considered in the same way as a patient with sporadic ALS.

ALS-related syndromes include

(1) **Monoclonal gammopathy** (monoclonal gammopathy of unknown significance, Waldenstrom's macroglobulinemia, osteosclerotic myeloma, etc.)
(2) **Dysimmune motor system degeneration** (autoimmune; high-titer GM_1 ganglioside antibody; etc.)
(3) **Nonmalignant endocrine abnormalities** (hyperthyroidism, hyperparathyroidism, hypogonadism, etc.)
(4) **Lymphoma** (Hodgkin's and non-Hodgkin's lymphoma). Cases of sporadic ALS associated with insulinoma, lung, colon or thyroid cancer are thought not to be causally related.
(5) **Infection** (HIV-1, HTLV-I, encephalitis lethargica, varicella-zoster, brucellosis, cat-scratch disease, Creutzfeldt-Jakob disease, syphilis, delayed post-poliomyelitis, etc.)
(6) **Acquired enzyme defects** (detoxification enzymes, etc.)
(7) **Exogenous toxins** (lead, mercury, arsenic, thallium, cadmium, manganese, aluminum, organic pesticides, lupin seeds, etc.)
(8) **Physical injury** (electric shock, radiation therapy, etc.)
(9) **Vascular** (vasculitis; ischemic (Dejerine anterior bulbar artery syndrome), etc.)
(10) **Spondylotic myelopathy** (painless myelopathy with no sensory signs, stabilization or progression post-surgery).

ALS variants

must meet the clinical, electrophysiological and neuroimaging criteria for possible, probable or definite ALS. The predominant presentation is that seen in sporadic ALS, but includes one or more features such as:

(1) **Familial pattern of inheritance** (multiple phenotypes characterized by age of onset; site of onset; length of survival; and presumed type of inheritance.)

Familial ALS variants in genetic linkage studies should be characterized by an established genetic mode of inheritance over at least two generations ***and*** at least

one clinically definite or autopsy confirmed case ***and*** compelling evidence excluding other possible causes. Affected sib pairs occurring in one generation alone may not result from a single gene effect.
Examples:
(a) ALS with defined inheritance and known gene product (hexosaminidase A/B deficiency, superoxide dismutase deficiency)
(b) ALS with defined inheritance and chromosome linkage but no gene product (chromosome 21 associated familial ALS or chromosome 2 associated juvenile familial ALS)
(c) ALS with defined inheritance and no known linkage or gene product (most cases of familial ALS).

(2) **Geographic clustering** (including disorders seen in the Western Pacific, Guam, Kii Peninsula, North Africa, Madras, etc.)
(3) **Extrapyramidal signs** (bradykinesia; cogwheel rigidity; tremor; clinically significant onset of supranuclear eye signs (pursuit abnormalities); familialor sporadic)
(4) **Cerebellar degeneration** (spinocerebellar abnormalities; familial or sporadic)
(5) **Dementia** (progressive cognitive abnormalities; familial or sporadic)
(6) **Autonomic nervous system involvement** (clinically significant abnormal cardiovascular reflexes; bowel or bladder control problems; familial or sporadic)
(7) **Objective sensory abnormalities** (decreased vibration; sharp-dull discrimination; blunting of cold sensation; familial or sporadic)

Electrophysiological features in the diagnosis of ALS

Patients with suspected, possible, probable or definite ALS on clinical grounds should have electrophysiological studies performed to confirm LMN degeneration in clinically affected regions, find electrophysiological evidence of LMN degeneration in clinically uninvolved regions and to exclude other pathophysiological processes.

ALS may be most reliably identified when the clinical and electrophysiological findings are widespread, involving a sufficient number of regions so that other possible causes of similar EMG abnormalities are highly unlikely. The confirmation of the diagnosis of ALS depends on finding electrophysiological evidence of LMN degeneration in at least two muscles of different root or spinal nerve and different cranial or peripheral nerve innervation in two or more of the four (bulbar, cervical, thoracic, lumbosacral) regions. The features of LMN degeneration in a particular muscle are defined by electromyographic needle examination and nerve conduction studies using standard methods for each measure.

Electrophysiological features ***required*** to identify ***definite*** primary LMN degeneration include all of the following:
(1) reduced recruitment (reduced interference pattern with firing rates over 10 Hz),
(2) large motor unit action potentials (large amplitude, long duration) and
(3) fibrillation potentials.

Electrophysiological features that ***support*** the identification of ***probable*** primary LMN degeneration include one or more of the following:
(1) reduced recruitment or large motor unit action potentials and fibrillation potentials and unstable motor unit action potentials or
(2) reduced motor unit estimates and increased macro EMG motor unit potentials.

Electrophysiological features that ***support*** the identification of ***possible*** primary LMN degeneration include one or more of the following:
(1) either reduced recruitment, large motor unit potentials, fibrillation potentials or unstable motor unit potentials alone,
(2) polyphasic motor unit potentials or increased single fiber density alone,
(3) low amplitude motor unit potentials if the disease duration is over 5 years or if there is associated atrophy,
(4) low amplitude compound muscle action potentials,
(5) compound muscle action potential change between proximal and distal sites of stimulation that is uniform along the length of the nerve,
(6) up to 30% decrement in motor conduction velocity below established normal values if a low amplitude compound muscle action potential greater than 10 percent of normal is present,
(7) up to 50% decrement in motor conduction velocity below established normal values if the compound muscle action potential is below 10% of normal,
(8) up to 20% decrement of the compound muscle action potential on 2 Hz repetitive stimulation,
(9) up to 10% decrement in sensory nerve conduction velocity and action potential amplitude from established normal values,
(10) complex repetitive discharges,
(11) absence of fasciculations.

Electrophysiological features ***compatible*** with UMN degeneration and not excluding ALS include one or more of the following:
(1) up to 30% increment in central motor conduction velocity,

(2) up to 10% decrement in somatosensory evoked potential amplitude and up to 10% increment in somatosensory evoked potential latency,
(3) mild abnormalities of autonomic function,
(4) mild abnormalities of polysomnography,
(5) mild abnormalities of electronystagmography.

Electrophysiological features that are ***inconsistent*** with the diagnosis of ALS or suggest the presence of additional other disease processes include one or more of the following:
(1) focal reduction in compound muscle action potential of more than 10% in a 4-cm segment,
(2) motor conduction velocities, F wave latencies or H wave amplitudes which are more than 30% above established normal values,
(3) more than 20% decrement on repetitive stimulation at 2 Hz,
(4) sensory action potential latencies more than 20% above or sensory action potential amplitudes more than 20% below established normal values,
(5) unstable motor unit potentials with no other electromyographic changes,
(6) small motor unit potentials with no other electromyographic changes,
(7) more than 30% increment of central motor conduction velocity,
(8) more than 10% increment in sensory evoked potential latency or more than 10% decrement in sensory evoked potential amplitude,
(9) moderate or greater abnormalities in autonomic function or electronystagmography.

Employing electrophysiological evidence of LMN degeneration to confirm the diagnosis of ALS

The certainty of LMN degeneration is determined by the presence of the above findings for each muscle tested in each region.

At least two muscles of different root or spinal nerve and different cranial or peripheral nerve innervation in each region should show electrophysiological evidence of either definite, probable or possible LMN degeneration for that region to be ranked as showing definite, probable or possible LMN degeneration.

Definite LMN degeneration by EMG has the same significance as clinical LMN degeneration and can upgrade the certainty of the clinical diagnosis of ALS in the same fashion as if the clinical signs of LMN degeneration were present in that region.

Probable or possible LMN degeneration by EMG does not carry the same weight as either clinical signs or definite electrophysiological evidence of LMN degeneration in a particular region.

However, the involvement of two regions with probable electrophysiological evidence of LMN degeneration or one region with probable and one region with possible electrophysiological evidence of LMN degeneration carries the same weight as one region with definite evidence of LMN degeneration in upgrading the certainty of the diagnosis of ALS.

A single region with electrophysiological evidence of probable LMN degeneration or two regions with electrophysiological evidence of possible LMN degeneration can be used to upgrade the certainty of the diagnosis of ALS from possible ALS to probable ALS but not from probable ALS to definite ALS.

Neuroimaging features in the diagnosis of ALS

Neuroimaging studies should be selected in order to exclude other conditions which may cause UMN and/or LMN signs that may simulate sporadic ALS.

Neuroimaging features ***required*** for the diagnosis of ALS:

There are no neuroimaging tests which confirm the diagnosis of ALS.

Neuroimaging features that ***support*** the diagnosis of ALS include one or more of the following:
(1) minimal bony abnormalities on **plain X-rays** of skull or spinal canal,
(2) minimal abnormalities on head or spinal cord **MRI** scans without spinal cord and/or root compression,
(3) minimal abnormalities on spinal cord **myelography** with post-myelography CT tomography showing no spinal cord and/or root compression.

Neuroimaging features that are ***inconsistent*** with the diagnosis of ALS include one or more of the following:
(1) significant bony abnormalities on **plain X-rays** of skull or spinal canal that might explain clinical findings,
(2) significant abnormalities of head or spinal cord **MRI** suggesting intraparenchymal processes, arteriovenous malformations or compression of brainstem/spinal cord and/or cranial nerve or spinal nerve roots by bony abnormalities, tumors, etc. MRI of craniocervical junction if bulbar onset *and/or* MRI of pertinent spinal region if spinal onset,
(3) significant abnormalities of spinal cord **myelography** with/without CT tomography or CT tomography alone suggesting lesions as noted above,
(4) significant abnormalities on spinal cord **angiography** suggesting arteriovenous malformations.

Employing neuroimaging evidence to confirm the diagnosis of ALS

The absence of abnormalities in appropriately performed neuroimaging studies will raise patients with clinical and/or electrophysiological evidence of probable ALS to definite ALS.

The absence of neuroimaging abnormalities cannot raise possible ALS to probable ALS.

Clinical laboratory features in the diagnosis of ALS

The diagnostic process employed to confirm the diagnosis of sporadic ALS includes repeated clinical examinations, repeated electrophysiological examinations, neuroimaging to exclude other disorders and clinical laboratory examinations to exclude other disorders or support the diagnosis of ALS related syndromes

Clinical laboratory features ***required*** for the diagnosis of ALS

There are no clinical laboratory tests which confirm the diagnosis of ALS.

Clinical laboratory features that ***support*** the diagnosis of ALS include one or more of the following:

- normal complete blood count, platelet count, sedimentation rate, prothrombin time,
- normal electrolyte (Na^+, K^+, Cl^-, CO_2, Ca^{2+}, Mg^{2+}, PO_4), renal (BUN, creatinine) and liver function (bilirubin, SGOT, SGPT, LDH) tests,
- creatine kinase (CK) elevation not more than 5 times upper limit of normal,
- normal hexosaminidase A and B activity (if possibility of deficiency indicated by suggestive family history or onset under 30 years of age),
- normal cerebrospinal fluid cell count, protein (not more than 65 mg/dl), absence of intrathecal immunoglobulin synthesis, oligoclonal immunoglobulins and evidence of elevated intrathecal antibodies to infectious agents (syphilis, HIV-1, HTLV-I, etc.), if indicated,
- normal parathyroid hormone level if calcium is borderline elevated,
- normal free thyroid hormone concentrations if any thyroid function abnormalities
- (borderline elevation in T_4, T_3, TSH); normal glycosylated hemoglobin, if indicated,
- normal serum protein electrophoresis and serum immunoelectrophoresis with immunofixation; normal urine immunoelectrophoresis with immunofixation, if indicated,
- minimal abnormalities in screening tests for collagen vascular diseases (anti-nuclear antibody; anti-DNA antibodies; rheumatoid factor; complement; anti-tissue specific antibodies), if indicated,
- minimal elevation in screening tests for anti-neural antigen (GM_1, GM_2, GD_{1b} gangliosides,myelin-associated glycoprotein, acetylcholine esterase, etc.) or anti-neuromuscular antigen (acetylcholine receptor, striated muscle, etc.) antibodies, if indicated.

Clinical laboratory features that ***support*** the diagnosis of ALS related syndromes

- abnormalities consistent with monoclonal gammopathy with/without significant elevation in monoclonal anti-neural antigen antibody,
- significant elevations in polyclonal anti-neural antigen (GM_1, GM_2, GD_{1b} gangliosides, myelin-associated glycoprotein, acetylcholine esterase, etc.) antibody,
- significant elevation in parathyroid hormone, thyroid hormone or other significant endocrine abnormalities,
- abnormalities consistent with lymphoma (Hodgkin's or non-Hodgkin's lymphoma),
- evidence of infection (HIV-1, HTLV-I, borrelia, syphilis, brucellosis, cat-scratch disease, varicella-zoster, influenza, Creutzfeldt-Jakob disease),
- evidence of intoxication (epidemiological evidence or elevated blood, urine, tissue or cerebrospinal fluid levels of lead, mercury, arsenic, cadmium, manganese, aluminum, organic pesticides, lupin seeds, etc.),
- evidence of physical injury (epidemiological evidence of antecedent electrical or radiation injury or severe trauma),
- evidence of vasculitis (elevated erythrocyte sedimentation rate and cerebrospinal fluid abnormalities consistent with spinal cord vasculitis, i.e., markedly elevated cerebrospinal fluid protein) or ischemic injury to spinal cord without sensory signs,
- evidence of preexisting mild or moderate spinal cord spondylotic compression, not amenable to surgical correction or not responding to surgical correction, which progressed with clinical signs consistent with at least probable ALS

Clinical laboratory features ***inconsistent*** with the diagnosis of ALS

There is no clinical laboratory finding which, if present with the proper clinical and electrophysiological signs of ALS and appropriate neuroimaging studies, rules out the diagnosis of ALS.

ALS-related syndromes which present with the ALS phenotype as defined have been described with laboratory abnormalities (acquired or genetic), time-linked exposure to chemical, physical or infectious agents and preexisting structural abnormalities. The correction of laboratory abnormalities, removal of chemical or physical agents, treatment of the associated disease (infection, tumor, structural abnormality) may or may not result in correction or stabilization of the ALS phenotype in ALS-related syndromes.

If correction of the abnormality results in clinical improvement the patient will be considered to have an ALS related syndrome.

If correction of the abnormality does not result in improvement then the patient will be considered to have sporadic ALS for the purposes of clinical studies and therapeutic trials.

Neuropathological features of the diagnosis of ALS

The clinical diagnosis may be **supported** or **excluded** by **biopsy** studies in the living patient and the **pathological diagnosis** may be **proven** or **excluded** by **autopsy** examination.

Pathological studies in the living patient with sporadic ALS

Indications for biopsies

Biopsies of the skeletal muscle, peripheral nerve and other tissues are not required for the diagnosis of amyotrophic lateral sclerosis, unless the clinical, electrophysiological or laboratory studies have revealed changes that are atypical for ALS. In addition, the muscle biopsy may be used to demonstrate LMN involvement in a body region that had not been shown to be involved by other techniques.

Muscle biopsy

Features ***required*** for the diagnosis:

Disseminated single angulated atrophic muscle fibers, or small or large groups of such fibers.

Features that strongly ***support*** the diagnosis:

Angulated atrophic muscle fibers that are strongly positive when stained with oxidative enzyme stains and with nonspecific esterase or that show immunoreactive surface staining with anti-NCAM antibodies.

Features that are ***compatible*** with, and do not exclude the diagnosis:

(1) Scattered hypertrophied muscle fibers.
(2) No more than a moderate number of target or targetoid fibers.
(3) Fiber type grouping of no more than mild-to-moderate extent.
(4) The presence of a small number of necrotic muscle fibers.

Features that ***rule out*** the diagnosis or suggest the presence of additional disease:

(1) Significant infiltration with lymphocytes and other mononuclear inflammatory cells,
(2) Significant arteritis,
(3) Significant numbers of muscle fibers involved with the following structural changes: necrosis; rimmed vacuoles; nemaline bodies; central cores; accumulation of mitochondria (ragged red fibers),
(4) Large fiber type grouping,
(5) Giant axonal swellings from accumulation of masses of neurofilaments, but not of PAS positive bodies, in intramuscular nerves.

Pathological studies at autopsy, other than in cases surviving for prolonged periods on life support systems

Gross pathological changes

Features ***required*** for the diagnosis:

There are no positive diagnostic features on gross pathological examination.

Features that ***support*** the diagnosis:

(1) Selective atrophy of the motor cortex,
(2) Grayness and atrophy of the anterior spinal nerve roots compared with normal,
(3) Grayness of the lateral columns of the spinal cord,
(4) Atrophy of skeletal muscles.

Features that ***rule out*** the diagnosis of ALS or suggest the presence of additional disease:

(1) Plaques of multiple sclerosis.
(2) A focal cause of myelopathy.

Light microscopic studies

Features ***required*** for the diagnosis:

(1) Some degree of loss of both of the following neuronal systems. Large motor neurons of the anterior horns of the spinal cord and motor nuclei of the brainstem (V motor, VII motor, IX and X somatic motor, and XII); and large pyramidal neurons of the motor cortex and/or large myelinated axons of the corticospinal tracts.
(2) The following cellular pathological changes in the involved neuronal regions described above: neuronal atrophy with relative increase in lipofuscin

and loss of Nissl substance. There should be evidence of different stages of the process of neuronal degeneration, including the presence of normal-appearing neurons, even in the same region.
(3) Evidence of degeneration of the corticospinal tracts at the same level.

Features that strongly ***support*** the diagnosis:
(1) Lack of pathological change in the motor neurons of cranial nerves III, IV and VI, the intermediolateral column of the spinal cord, and Onuf's nucleus.
(2) The occurrence of one or more of the following cellular pathological changes in the involved neuronal systems described above:
Axonal spheroids with accumulation of masses of neurofilaments,
Bunina bodies,
Basophilic cytoplasmic inclusions,
Non-basophilic hyaline bodies ("Lewy body-like structures") seen in H&E stained sections,
Increased immunocytochemical staining for phosphorylated neurofilaments in perikarya of the motor neurons;
Ubiquinated intracytoplasmic inclusions in the motor neurons;
Atrophy or loss of the arborizations of the dendrites of the motor neurons of the anterior horns of the spinal cord and the brainstem motor nuclei.
Wallerian degeneration in the anterior roots.

Features that are ***compatible*** with, and do not exclude, the diagnosis:
Variable involvement of Clarke's nucleus and the spinocerebellar tracts; posterior root ganglia, the posterior columns of the spinal cord and peripheral sensory nerves; the brainstem reticular neurons and the anterolateral columns of the spinal cord; the thalamus; subthalamic nucleus; and the substantia nigra.

Features that ***rule out*** the diagnosis or suggest the presence of additional disease:
Major pathological involvement of other parts of the nervous system, including: cerebral cortex other than the motor cortex; basal ganglia; substantia nigra; cerebellum; cranial nerves II and VIII; dorsal root ganglia.
The following cellular pathological changes in the involved neuronal systems described above:
Extensive central chromatolysis;
Extensive active neuronophagia;
Neurofibrillary tangles;
The presence of abnormal storage material;
The presence of significant spongiform change;
The presence of extensive inflammatory cell infiltration.

Electron-microscopic studies

Features ***required*** for the diagnosis:
Ultrastructural studies are not required for the diagnosis of ALS.

Features that strongly ***support*** the diagnosis:
(1) Accumulation of interwoven bundles of 10 nm neurofilaments in axonal spheroids or motor neuron perikarya, and thicker linear structures associated with dense granules (Hirano et al. 1984 J. Neuropath. Exp. Neurol. 43: 461),
(2) Bunina bodies (Hart et al. 1977 Acta Neuropath. 38: 225)

Features that are ***compatible*** with, and do not exclude the diagnosis:
The presence of intra-axonal polyglucosan bodies.

Features that ***rule out*** the diagnosis or suggest the presence of additional disease:
(1) The presence of significant numbers of definite viral particles,
(2) The presence of significant amounts of abnormal storage material,
(3) Extensive vacuolation of neuronal perikarya.

Participants of the "Clinical Limits of ALS" Workshop

Jack Antel, MD
Montreal Neurological Institute
3801 University Street
Montreal, Quebec, Canada H3A 2B4

Walter Bradley, MD
Burlington, VT, USA

Benjamin Rix Brooks, MD
University of Wisconsin-Madison
ALS Clinical Research Center
CSC J6-504
Madison, WI 53792, USA

Peter Cardy
Director, Motor Neurone Disease Association
P.O. Box 246
Northampton NN1 2PR, UK

Stirling Carpenter, MD
Montreal, Quebec, Canada

Samuel Chou, MD
Cleveland, OH, USA

Sebastian Conradi, MD
Neurology Department
Karolinska Sjukhuset
10401 Stockholm, Sweden

Jasper Daube, MD
Mayo Clinic
Rochester, MN, USA

Eric H. Denys, MD
ALS Center
2100 Webster Street, Suite 110
San Francisco, CA 94115, USA

Barry Festoff, MD
Neurobiology [151]
DVA Medical Center
4801 Linwood Blvd
Kansas City, MO 64128, USA

Asao Hirano, MD
Montefiore Hospital
Bronx, NY, USA

Adilia Hormigo, MD
Departamento de Neurologia, C VIII
Instituto Portugues de Oncologia
RVA Prof. Lima Basto
1093 Lisbon Codex, Portugal

George Karpati, MD
Montreal, Quebec, Canada

Edward Kasarskis, MD
Neurology Department
University of Kentucky Medical School
800 Rose Street
Lexington, KY 40536-0084, USA

Gerald Kuther, MD
Neurologische Klinik der Technischen Hochschule
Nohlstraße 28
8000 Munich 80, Germany

Rosa Larumbe, MD
Departamento de Neurologia
Clinica Universitaria de Navarra
c/ Pio XII s/n
Pamplona, Spain

Nigel Leigh, MD
Institute of Psychiatry
London, UK

J. Manuel Martinez-Lage, MD
Departamento de Neurologia
Clinica Universitaria de Navarra
Apartado 192
31080 Pamplona, Spain

Vincent Meininger, MD
Centre SLA Centre de Diagnostic
Hôtel Dieu
1 Rue de la Cité
75004 Paris, France

Douglas Mitchell, MD
Royal Preston Hospital
Smaroe Green Lane
Fulwood
Preston PR2 4HT, UK

Jesus S. Mora, MD
Unidad Neuromuscular
Clinica Puerta de Hierro
c/San Martin de Porres, 4
28035 Madrid, Spain

Forbes Norris, MD†
ALS/Neuromuscular Research Foundation
Pacific Presbyterian Medical Center
2351 Clay Street, Suite 416
San Francisco, CA 94115, USA

Alberto Portera-Sanchez, MD
Neurology Department
Hospital Universitario "12 de Octubre"
Carretera de Andalucia Km 5
28401 Madrid, Spain

F. Clifford Rose, MD
Academic Unit of Neuroscience
Charing Cross Hospital
Fulham Palace Road
London W6 8RF, UK

Guy Rouleau, PhD
Neurology Department
Montreal General Hospital
1650 Cedar Street
Montreal, Quebec, Canada

† Deceased.

Michael Swash, MD
Department of Neurology
Royal London Hospital
Whitechapel, London E1 1BB, UK

Janez Zidar, MD
Institute of Clinical Neurophysiology
University Medical Center
Zaloska 7 61105
Ljubljana, Slovenia

ALS Neuropathology Subsection

Walter Bradley, MD (Chairman)
Burlington, VT, USA

Stirling Carpenter, MD
Montreal, Quebec, Canada

Samuel Chou, MD
Cleveland, OH, USA

Asao Hirano, MD
Montefiore Hospital
Bronx, NY, USA

George Karpati, MD
Montreal, Quebec, Canada

Nigel Leigh, MD
Institute of Psychiatry
London, UK

ALS Neurophysiology Subsection

Michael Swash, MD (Chairman)
Royal London Hospital
London E1 1BB, UK

Jasper Daube, MD
Mayo Clinic Rochester, MN, USA

Eric H. Denys, MD
ALS Center
2100 Webster Street, Suite 110
San Francisco, CA 94115, USA

Gerald Kuther, MD
Neurologische Klinik der Technischen Hochschule
Nohlstraße 28
8000 Munich 80, Germany

Jesus S. Mora, MD
Unidad Neuromuscular
Clinica Puerta de Hierro
c/San Martin de Porres, 4
28035 Madrid, Spain

Janez Zidar, MD
Institute of Clinical Neurophysiology
University Medical Center
Zaloska 7 61105
Ljubljana, Slovenia

ALS Laboratory Subsection

Barry Festoff, MD (Chairman)
Neurobiology [151]
DVA Medical Center
4801 Linwood Blvd
Kansas City, MO 64128, USA

Jack Antel, MD
Montreal Neurological Institute
3801 University Street
Montreal, Quebec, Canada H3A 2B4

Sebastian Conradi, MD
Neurology Department
Karolinska Sjukhuset
10401 Stockholm, Sweden

Edward Kasarskis, MD
Neurology Department
University of Kentucky Medical School
800 Rose Street
Lexington, KY 40536-0084, USA

Vincent Meininger, MD
Centre SLA Centre de Diagnostic
Hôtel Dieu
1 Rue de la Cité
75004 Paris, France

Guy Rouleau, PhD
Neurology Department
Montreal General Hospital
1650 Cedar Street
Montreal, Quebec, Canada

ALS Clinical Subsection

Benjamin Rix Brooks, MD (Chairman)
University of Wisconsin-Madison
ALS Clinical Research Center
CSC J6-504
Madison, WI 53792, USA

Peter Cardy
Director, Motor Neurone Disease Association
P.O. Box 246
Northampton NN1 2PR, UK

Adilia Hormigo, MD
Departamento de Neurologia, C VIII
Instituto Portugues de Oncologia
RVA Prof. Lima Basto
1093 Lisbon Codex, Portugal

Rosa Larumbe, MD
Departamento de Neurologia
Clinica Universitaria de Navarra
c/ Pio XII s/n
Pamplona, Spain

J. Manuel Martinez-Lage, MD
Departamento de Neurologia
Clinica Universitaria de Navarra
Apartado 192
31080 Pamplona, Spain

Douglas Mitchell, MD
Royal Preston Hospital
Smaroe Green Lane
Fulwood
Preston PR2 4HT, UK

Forbes Norris, MD†
ALS/Neuromuscular Research Foundation
Pacific Presbyterian Medical Center
2351 Clay Street, Suite 416
San Francisco, CA 94115, USA

Alberto Portera-Sanchez, MD
Neurology Department
Hospital Universitario "12 de Octobre"
Carretera de Andalucia Km 5
28401 Madrid, Spain

F. Clifford Rose, MD
Academic Unit of Neuroscience
Charing Cross Hospital
Fulham Palace Road
London W6 8RF, UK

† Deceased.

El Escorial Criteria for the Diagnosis of ALS

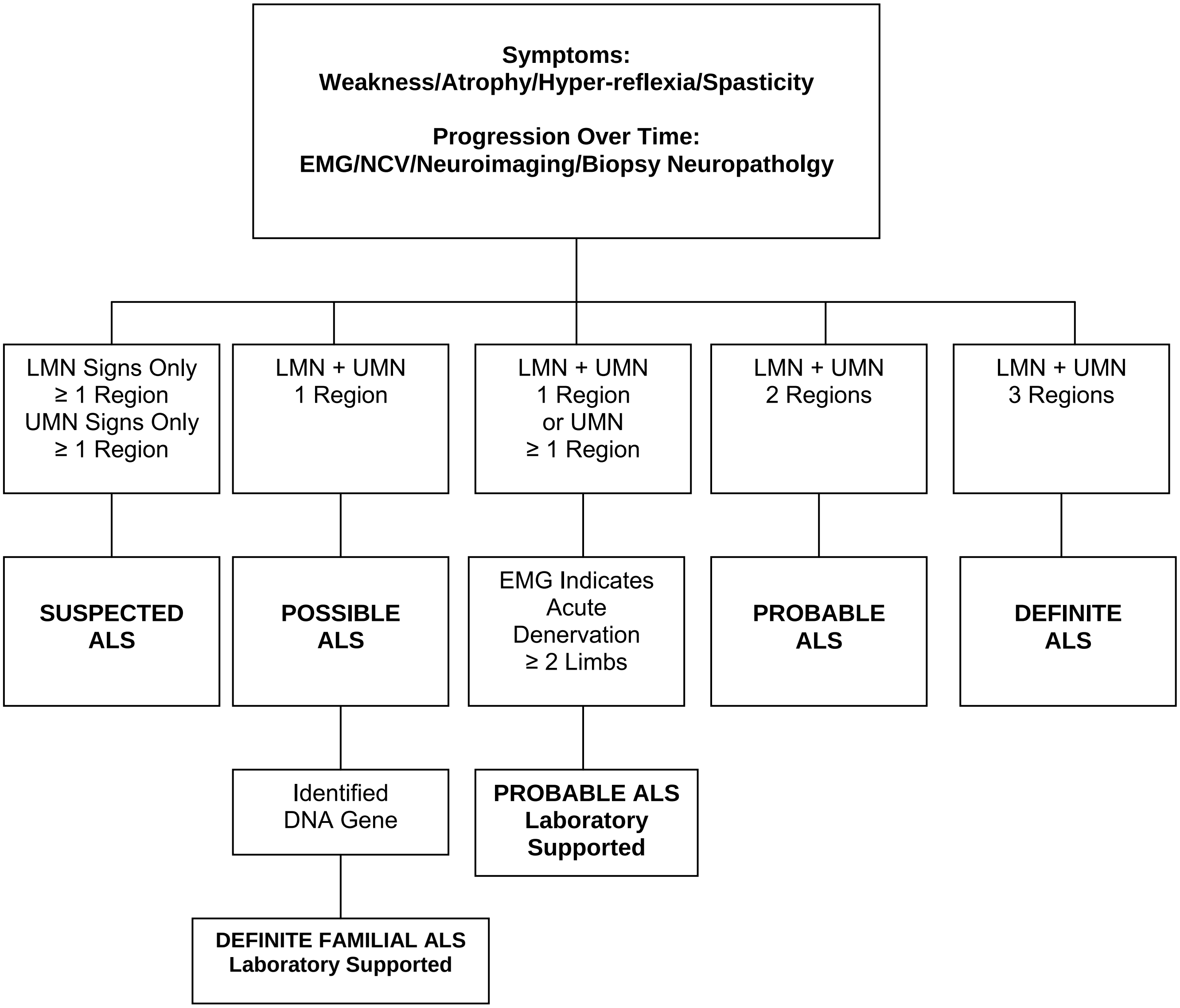

Protocol Appendix 3

MODEL
Informed Consent Forms

- STAGE 3 MODEL Consent
- Caregiver MODEL Consent

CONSENT TO PARTICIPATE IN A CLINICAL RESEARCH STUDY

SUBJECTS WITH ALS

<u>PROTOCOL TITLE</u>: Clinical Trial of Ceftriaxone in Subjects with Amyotrophic Lateral Sclerosis (ALS)

Sponsor: National Institutes of Health/ National Institutes of Neurological Disorders and Stroke (NIH/NINDS)

Protocol Number: CEF-ALS-2006

About this consent form

Please read this form carefully. It tells you important information about a research study. A member of our research team will also talk to you about taking part in this research study. People who agree to take part in this research study are called "subjects." This term will be used throughout this consent form. If you have any questions about the research or about this form, please ask us. If you decide to take part in this research study, you must sign this form to show that you want to take part. We will give you a copy of this form to keep.

Why is this research study being done?

We would like permission to enroll you as a subject in our research study. The purpose of this study is to evaluate the safety and effect of ceftriaxone treatment in subjects with amyotrophic lateral sclerosis (ALS). We are asking you to take part in this study because you have been diagnosed as having ALS.

It is known that nerve cells called "motor neurons" die in the brains and spinal cords of people with ALS. However, the cause of the cell death is unknown. Researchers think that increased levels of a chemical called "glutamate" may be related to the cell death. For this reason researchers want to study drugs that decrease glutamate levels near nerves. We are interested in studying ceftriaxone because the drug may increase the level of a protein that decreases glutamate levels near nerves. Studies of ceftriaxone in the laboratory suggest that it may protect motor neurons from injury.

Ceftriaxone is approved by the U.S. Food and Drug Administration (FDA) for the treatment of bacterial infections but not for the treatment of ALS. Ceftriaxone is also not approved for daily use for longer than 6 weeks. The use of ceftriaxone for the treatment of ALS is investigational. This means that ceftriaxone is being tested in research studies as a possible treatment for ALS. Ceftriaxone has not been given to people over a long period of time, such as months or years. The long-term use of ceftriaxone is also being studied.

How long will I participate in this research study?

Subjects will participate in this study until the last subject enrolled has taken the study drug for 52 weeks (one year). The length of time it takes you to complete the study will depend upon when you started the study. The shortest time to complete the study will be 52 weeks. The longest time to complete the study will be 5 years. Over the 5 years, subjects will be asked to make up to 70 visits to **[INSERT SITE NAME].**

What will happen in this research study?

This is a double-blind study where the subjects are selected by chance (like tossing a coin) to be in one of two groups. “Double-blind” means that neither you, the investigator (study doctor), nor any of the study staff at your site (other than the person preparing the drug for the study doctor) will know which drug you are receiving, although this information will be available in case of an emergency.

This research study will be done in three STAGES. Sixty subjects have already completed STAGES 1 & 2.

STAGES 1 & 2 consisted of a double-blind study where the subjects were selected by chance (like tossing a coin) to be in one of three groups. These 60 subjects will now continue on to be part of STAGE 3 of the study.

Before beginning STAGE 3, the information from STAGES 1 and 2 has been looked at by a safety committee (Data and Safety Monitoring Board, appointed by the National Institutes of Health’s neurology division, called NINDS) and the committee responsible for guiding the progress of the trial (the Steering Committee, made up of members who are in the fields involved in the study), and a decision has been made that the trial is able to move forward, based on safety data. The dose for STAGE 3 has also been determined by these groups based on the data collected in STAGES 1 & 2.

An additional 540 subjects will participate in STAGE 3 of this study. If you choose to participate, you will be one of these 540 subjects. At the end of the study, a total of 600 subjects will have participated in the study at up to 70 sites across the US and Canada. We plan to screen a total of about 30 people with ALS for the study at **[INSERT SITE NAME]** to find 15 who meet the study requirements.

You will be given the study drug (ceftriaxone or placebo) twice a day through a central venous catheter placed in your neck. A central venous catheter is used for long-term intravenous (IV) access. Central venous catheters are used when patients need many weeks or a few months of treatment with antibiotics, chemotherapy, or nutritional support. The central venous catheter will be used only to give you the study drug and cannot be used for other purposes during the study. The catheter may have to be replaced

during the study. The likelihood of the catheter needing replacement is unknown and will depend on side effects due to the catheter.

This study uses a placebo. The placebo looks like the study drug, but contains no ceftriaxone. Placebos are used to tell whether the effects seen are really from the study drug. The placebo used for this study is a low dose of pediatric multi-vitamin.

If you are selected to receive ceftriaxone as your study drug, you will also receive a medication (ursodiol) in the form of a tablet that you will take two times a day. This medication is being used to lower the risk of developing gallbladder problems, which is an expected side effect of ceftriaxone. If you are selected to receive placebo study drug, you will receive a placebo medication (instead of ursodiol) in the form of a tablet that you will take two times a day. This placebo medication will look like ursodiol, but will contain no active drug.

You cannot take part in this study if you:

- are less than 18 years old
- Are pregnant
- Are allergic to Penicillin or other cephalosporin antibiotics, such as Ancef, Keflex, Ceclor, Ceftin, Lorabid, Suprax, and Fortaz.
- Have a history of another neurological disorder (other than ALS)

During STAGE 3 (the stage in which you are entering the trial), research subjects will be assigned randomly (by chance, like a coin toss) to one of two study groups:

One group (2/3, or 67%, of subjects) will receive 4 grams per day of ceftriaxone

One group (1/3, or 33%, of subjects) will receive Placebo

STUDY VISITS

The first visit for the study is the Screening Visit. The purpose of this visit is to find out if you meet all of the requirements to participate in the study. The second visit is the Central Venous Catheter Placement Visit. Upon successful placement of the Central Venous Catheter the subject will be "randomized" into the study and the Baseline Visit will be performed.

Following the Baseline Visit, you will return to **[INSERT SITE NAME]** each week for the next two weeks, and then every 4 weeks for the duration of the study.

Description of Procedures

Abdominal Ultrasound: An ultrasound is a test that uses sound waves to get a picture of your insides, in this case your gallbladder. You will lie on a table and the doctor or nurse will use a transducer (hand-held device) and gel that is applied to your skin, running the

transducer over the skin of your abdomen. Ultrasound waves cause no sensation. The only thing you will feel is the pressure of the transducer on your skin.

Vital Capacity (VC) Testing: The VC measures the maximum amount of air you can exhale following a deep breath. For this test, you will be asked to hold a mouthpiece in your mouth, breathe in deeply, and breathe out as long and hard as you can.

ALS Functional Rating Scale – Revised (ALSFRS-R) Questionnaire: this questionnaire consists of 12 questions about your ability to function in certain daily activities. Although we hope you will answer all questions, you can skip any questions that you do not want to answer. This questionnaire will take about 5-10 minutes to answer.

ALS-Specific Quality of Life (ALSSQOL) Questionnaire and Caregiver Burden Inventory: You and your caregiver will be asked to fill out questionnaires that pertain to your quality of life. One (that you will fill out) is an ALS-specific Quality of Life Scale, and the other (that your caregiver will fill out) is called the Caregiver Burden Inventory. You can skip any questions that you do not want to answer. These questionnaires will take about 15-20 minutes to answer.

Strength testing: You will have muscle strength testing performed on your upper and lower limbs. For this procedure, the coordinator will hold a small device (called a hand-held dynamometer) in his or her hand and will push against your arms and legs while you try to hold against this pushing. This testing will take approximately 15 minutes. This should not hurt, but may be slightly uncomfortable due to pressure and may make your muscles tired.

Screening Visit

The Screening Visit will take about two hours. During this visit, the following procedures will be done to see if you meet the study requirements and are eligible to take part in the study:

- Obtain written informed consent
- Assessment of inclusion and exclusion criteria
- Review of medical history
- Complete physical exam
- Vital signs (blood pressure, heart and respiratory rates, temperature) and weight
- Review of current medications
- Blood drawn for routine laboratory tests (about 2 teaspoons)
- For women able to have children, blood drawn for pregnancy test (1 teaspoon)
- Urine collected for routine tests
- Vital capacity test (VC) to check breathing capacity
- Abdominal ultrasound to check for gallbladder disease
- ALS Functional Rating Scale, Revised – ALSFRS-R (a questionnaire about ability to function in certain daily activities)

- Complete ALS Specific Quality of Life Questionaire (ALSSQOL)
- Strength testing of arm and leg muscles

The investigator (doctor) or research nurse will call you in about one week to tell you if you are eligible to take part in this study. If you are eligible, you will be asked to return within 26 days of the screening visit to have a central venous catheter placed. The study drug (ceftriaxone or placebo) will be given through this catheter.

Central Venous Catheter Placement Visit

Placement of the central venous catheter will take place in the Interventional Radiology Department at **[INSERT SITE NAME]**. The procedure will be explained to you in detail by the radiologist or nurse practitioner that will do the procedure. You may be asked by the radiologist to sign a separate consent form to show that you agree to have the procedure done. This visit will take about **6 hours**. Except in rare cases, this visit will not require an overnight stay in the hospital.

Before the central venous catheter is placed we will check your vital signs and review any changes in the status of your condition and your current medications. Placement of a central venous catheter involves the following:

> The radiologist uses ultrasound to see if the vein at the base of your neck is suitable (easily identified). Before the procedure, an intravenous (IV) catheter is placed in a vein in your arm to give IV antibiotics to prevent infection. An IV catheter is a very thin flexible plastic tube that is inserted into a vein using a needle. We may give you medication before the procedure to help you relax. The area is cleaned thoroughly with an antiseptic solution and sterile cloths are placed over the area to lower the risk of infection. A local anesthetic is injected to numb the area.
>
> Once the area is numb, the radiologist makes a small cut in the skin. A needle is then inserted through the cut in the skin and placed in the vein at the base of the neck, known as the jugular vein. The radiologist creates a tunnel under the skin from the upper chest to the neck incision. The catheter is passed through the tunnel and into the jugular vein. The tip of the catheter is placed in the main vein of the chest. The radiologist uses ultrasound to guide and check the placement of the catheter. In some cases, the radiologist may also use a type of X-ray, called "fluoroscopy" to guide the catheter placement as well. As part of the catheter placement, you may also be given medications, including antibiotics.
>
> One or two stitches are used to close the small cut at the base of the neck and a stitch is also used around the catheter on the outside to hold it in place. The catheter will exit the skin in the upper chest, and will look something like an IV. The place where the catheter exits the skin is called the "exit site."

A Dacron-fiber cuff is placed below the skin to hold the catheter in place and to prevent infection. Over time, body fluids cause the cuff to expand and tissue forms in and around it to hold it in place.

Following the placement procedure, an x-ray is done to check the catheter placement. You will stay in the Interventional Radiology suite for about two hours to make sure that you are doing well and that there are no problems.

The catheter extends outside the chest by about 12 inches. While this may be accepted easily for some, it may cause negative feelings related to body image or sexuality issues for others. Once the wound heals, it is safe to do most normal activities of daily living. Sports activity is permitted except for contact sports where there is a risk of the catheter being pulled out. Showering is permitted, once the incision is healed and this is approved by the study nurse or doctor. The dressing site should be covered with plastic and securely fastened around the edges with tape to prevent the dressing from getting wet. If the dressing does get wet, it should be changed. The catheter should not be placed underwater (taking a tub bath, hot tub, etc). Swimming is also not permitted.

During the study, if you need to have the catheter removed temporarily, you may have to stop taking the study drug until the catheter is replaced. In some situations, you may be given the option of receiving the study drug through a different kind of intravenous (IV) line, called a peripheral line, until the catheter can be replaced. If this is necessary, your study doctor will discuss the options with you.

Pre –Randomization Central Venous Catheter Check.

After your catheter is placed, and before you have your Baseline visit (when you will begin taking the study drug), a member of the site staff will check the catheter and the exit site. This "check" is to make sure that the catheter is functioning properly and that there are no signs of fever or infection. The visit may be done at **[SITE NAME]**, or at your home. The visit will occur only if the Catheter Placement and Baseline Visits do not occur on the same day. If there are any problems with the catheter (if it is not working or if the medical person who checks it feels that it is infected), you will not be able to start study drug until the problem is resolved.

Baseline Visit (Day 0)

The Baseline Visit will take place no more than 28 days after the screening visit. This visit may take place on the same day as the central venous catheter placement visit, or on a different day. The baseline visit will take about **2 hours**. During this visit, the following procedures will be done:

- Review of changes in your condition
- Review of current medications
- Vital signs and weight

- Check of central venous catheter site
- Distribution of study drug, and ursodiol or placebo medication

At this visit you will begin taking the study drug (ceftriaxone or placebo). You will take the study drug twice a day. The study drug is provided in pre-filled syringes (small containers that hold the drug and attach to the tubing). The pre-filled syringes must be kept in the freezer until you are ready to use them. The study drug must be thawed before use and can not be re-frozen after it has been thawed.

Giving you the study drug includes inserting the syringe into the pump, preparing the tubing, connecting the tubing to the end of the catheter and setting the pump. Before you begin taking the study drug, we will teach you and your caregiver how to give the study drug. You will have time to practice and ask questions. We will also give you and your spouse/caregiver detailed written instructions on how to store, thaw, and give study drug. Your first batch of pre-filled study drug syringes will be given to you at this visit. You will be asked to bring any unused study drug and the empty syringes with you at each study visit.

For the first two weeks, a nurse that specializes in home infusion catheters will be available to come to your home if needed. The home infusion nurse will look at your catheter and check for signs of infection. In addition, the nurse will make sure you know how to take care of the catheter, how to take the study drug and how to change the dressing. The nurse may continue to visit you in the home if the site investigator **[INSERT SITE INVESTIGATOR NAME]** thinks that it is medically necessary.

At this visit you will also receive a bottle of ursodiol or placebo medication. You will take one tablet of this medication twice a day. You will be asked to bring any unused medication with you at each study visit.

Following the Baseline Visit, you will return to **[INSERT SITE NAME]** every week for the next two weeks, and then every four (4) weeks for the rest of the study.

Week 1 and Week 2 Visits

These study visits will each take about **2 hours**. During these visits, the following procedures will be done:

- Review of side effects or changes in your condition
- Review of current medications
- Vital signs
- Check of central venous catheter site
- Blood drawn for routine laboratory tests (just over 2 teaspoons)
- Urine collected for routine tests of kidney function

- Review of Catheter Care and study drug administration procedures with you and your caregiver as needed

Week 4 Visit

You will be given a 4 week supply of study drug (ceftriaxone or placebo) along with ursodiol or matching placebo medication at this visit. You will bring your filled-out study diary, unused tablets and study drug syringes (empty and un-used) to this visit. During this visit the following procedures and tests will be performed:

- Review of side effects or changes in your condition
- Review of current medications
- Vital signs and weight
- Check of central venous catheter site
- Blood drawn for routine laboratory tests (just over 2 teaspoons)
- Urine collected for routine tests of kidney function
- Vital capacity test (VC) to check breathing capacity
- ALS Functional Rating Scale, Revised (ALSFRS-R)
- ALS Specific Quality of Life Questionaire (ALSSQOL)
- Strength testing of arm and leg muscles
- An abdominal ultrasound to check for gallbladder disease*

* The abdominal ultrasound procedure will be performed at Week 4, Week 8, and Week 20 visits. If an abdominal ultrasound shows evidence of gall bladder problems, an abdominal ultrasound will be repeated at regular intervals until this condition resolves.

Week 8 Visit

You will be given a 4 week supply of study drug (ceftriaxone or placebo) along with ursodiol or matching placebo medication at this visit. You will bring your filled-out study diary, unused tablets and study drug syringes (empty and un-used) to this visit. During this visit the following procedures and tests will be performed:

- Review of side effects or changes in your condition
- Review of current medications
- Vital signs
- Check of central venous catheter site
- Blood drawn for routine laboratory tests (just over 2 teaspoons)
- Urine collected for routine tests of kidney function
- An abdominal ultrasound to check for gallbladder disease*

*abdominal ultrasound will be performed at Week 4, Week 8, and Week 20, and then as needed throughout the study. If an abdominal ultrasound is performed and shows

evidence of gall bladder problems, additional ultrasounds may be performed at regular intervals until the problem resolves.

These same procedures will be performed at the Week 20 Visit

Week 12 Visit

You will be given a 4 week supply of study drug (ceftriaxone or placebo) along with ursodiol or matching placebo medication at this visit. You will bring your filled-out study diary, unused tablets and study drug syringes (empty and un-used) to this visit. During this visit the following procedures and tests will be performed:

- Review of side effects or changes in your condition
- Review of current medications
- Vital signs
- Check of central venous catheter site
- Blood drawn for routine laboratory tests (just over 2 teaspoons)
- Urine collected for routine tests of kidney function

These same procedures will be performed at the Week 28, 36, 44, 52**, 60, 68, 76, 84, 92, 100, 108, 116, 124, 132, 140, 148 and 156 Visits
**At the Week 52 Visit, a Complete Physical Examination will be performed in addition to the tests listed above.

Week 16 Visit

You will be given a 4 week supply of study drug (ceftriaxone or placebo) along with ursodiol or matching placebo medication at this visit. You will bring your filled-out study diary, unused tablets and study drug syringes (empty and un-used) to this visit. During this visit the following procedures and tests will be performed:

- Review of side effects or changes in your condition
- Review of current medications
- Vital signs and weight
- Check of central venous catheter site
- Blood drawn for routine laboratory tests (just over 2 teaspoons)
- Urine collected for routine tests of kidney function
- Complete physical exam
- Additional blood drawn (2 teaspoons) to measure ceftriaxone levels
- Vital capacity test (VC) to check breathing capacity
- ALS Functional Rating Scale, Revised (ALSFRS-R)
- ALS Specific Quality of Life Questionaire (ALSSQOL)
- Strength testing of arm and leg muscles

Week 24 Visit

You will be given a 4 week supply of study drug (ceftriaxone or placebo) along with ursodiol or matching placebo medication at this visit. You will bring your filled-out study diary, unused tablets and study drug syringes (empty and un-used) to this visit. During this visit the following procedures and tests will be performed:

- Review of side effects or changes in your condition
- Review of current medications
- Vital signs
- Check of central venous catheter site
- Blood drawn for routine laboratory tests (just over 2 teaspoons)
- Urine collected for routine tests of kidney function
- ALS Functional Rating Scale, Revised (ALSFRS-R)

These same procedures will be performed every sixteen weeks at Week 40, 56, 72, 88, 104, 120, 136 and 152 Visits.

Week 32 Visit

You will be given a 4 week supply of study drug (ceftriaxone or placebo) along with ursodiol or matching placebo medication at this visit. You will bring your filled-out study diary, unused tablets and study drug syringes (empty and un-used) to this visit. During this visit the following procedures and tests will be performed:

- Review of side effects or changes in your condition
- Review of current medications
- Vital signs and weight
- Check of central venous catheter site
- Blood drawn for routine laboratory tests (just over 2 teaspoons)
- Urine collected for routine tests of kidney function
- Additional blood drawn (2 teaspoons) to measure ceftriaxone levels
- Vital capacity test (VC) to check breathing capacity
- ALS Functional Rating Scale, Revised (ALSFRS-R)
- ALS Specific Quality of Life Questionaire (ALSSQOL)
- Strength testing of arm and leg muscles

These same procedures will be performed every sixteen weeks at Week 48, 64, 80, 96, 112, 128 and 144 Visits.

Final Study Visit

You will bring your filled-out study diary, unused tablets and study drug syringes (empty and un-used) to this visit. Some or all of the following procedures may be performed:

- Review of side effects or changes in your condition
- Review of current medications
- Vital signs and weight
- Check of central venous catheter site
- Blood drawn for routine laboratory tests (just over 2 teaspoons)
- Urine collected for routine tests and tests of kidney function
- Vital capacity test (VC) to check breathing capacity
- ALS Functional Rating Scale, Revised (ALSFRS-R)
- ALS Specific Quality of Life Questionaire (ALSSQOL)
- Strength testing of arm and leg muscles
- Complete physical exam

Other procedures may be performed or additional safety laboratory tests may be drawn (including coagulation studies) at visits, based on medical necessity, as determined by the Site Investigator or other physician.

Catheter Removal

The catheter will be removed by a doctor or Nurse Practitioner who specializes in either Surgery, Interventional Radiology, or who is otherwise qualified to remove the catheter.

Catheter Removal Process:

- An antiseptic will be put on your skin around the catheter site to clean bacteria from the skin.
- An injection of medication to numb the area at and around the exit site will be given. This will produce some discomfort from the needle prick and a temporary burning feeling when the medication is given. The area will become numb within a minute or two.
- The doctor will then separate the skin and other tissue near the exit site from the catheter. This is the area under the skin that has grown into the cuff holding the catheter in place.
- Rarely, a small incision may need to be made in the skin to allow the doctor to better reach this cuff. The sensation of releasing the tissue will produce a feeling of pressure.
- When the catheter is released from the skin, the doctor will pull back the catheter until it is removed.
- Pressure will be put on the neck area where the catheter entered the vein. This will be held in place for several minutes to prevent bleeding under the skin. The doctor may put a suture or “stitch” at the exit site opening or, if an incision was made, to the incision.
- As needed, medications may be given (including antibiotics), or additional procedures performed, as part of the catheter removal.

Blood Draws

The amount of blood you will have drawn over the entire course of the study will depend on how long (between 1 year and 5 years) you are in the study. The maximum amount of blood that will be drawn over 5 years is 1,050 ccs, which is a little more than giving 2 pints of blood.

Post Treatment Telephone Call

You will be contacted by telephone by the study coordinator 30 days after the Final Study Visit. This phone call will take about 5 minutes. You will be asked questions about any side effects you may have experienced and your overall health, including pregnancy status for women, since stopping the study drug. You will also be asked about your current medications. In addition, the study staff may ask your permission to call you once or twice, up to 5 years after you discontinue study drug, to inquire about your status.

Subject Responsibilities

As a subject in this trial, you have certain responsibilities to help ensure your safety. Please follow these important responsibilities listed below:

- It is very important that you keep all scheduled appointments.
- Take the study drug as prescribed by the study doctor
- Return all empty and unused study drug syringes at each visit
- Store the drug properly
- Report all side effects and medical problems to the study staff
- Complete your study diaries as instructed
- If you decided to stop taking part in the study, you must inform the study doctor or staff **before** you stop taking the study drug. You will still have study visits, where you will perform vital capacity testing, strength testing, and ALS Functional Rating Scale, Revised, even if you stop taking study drug.
- Contact Dr. **[INSERT SITE INVESTIGATOR NAME]** before starting any new medications or changing current medications. This includes prescription drugs, over-the-counter drugs, and anything else, like herbal remedies. You may not take any other investigational drugs for ALS while you are taking part in this study.

Because there may be a reaction between different drugs, it is important that you tell your doctor (the investigator) if you are on blood thinners. If you are taking blood thinning medication such as coumadin (warfarin), the dose of coumadin may need to be changed while you are receiving ceftriaxone. You should have your blood tested regularly by the physician prescribing your coumadin to make sure you are on the correct dose.

If you require a typhoid vaccination while you are in the study (usually if you are traveling), it is important that you tell the investigator and the doctor giving you the

vaccine all of your medications ahead of time. Antibiotics including ceftriaxone can interfere with the development of an adequate immune response to the oral typhoid vaccine. Because of this, you should not take the oral (by mouth) typhoid, and instead you should request (and receive) a different preparation of the typhoid vaccine.

If you receive any IV treatment that contains calcium (such as nutrition through an IV (called "parenteral" nutrition or TPN)), you should let your study doctor know immediately. There have been reports of interaction between these products, which contain calcium, and ceftriaxone in babies. Because of this, these products should not be given within 48 hours of ceftriaxone, even in adults.

If you wish to stop the study drug, you should tell Dr. **[INSERT SITE INVESTIGATOR NAME**] so that **he/she** may plan for your continued medical care. **[INSERT SITE INVESTIGATOR NAME]** may decide not to enter you in the study or to stop your participation without your permission if **he or she** feels that you cannot follow the study plan or if your health is in question. Side effects from the study drug or the central line would be examples of reasons for stopping the study drug. In addition, if certain unexpected effects occur (either harmful or beneficial), the entire study may be stopped.

What are the risks to me?

Ceftriaxone (Study Drug):

Ceftriaxone is FDA approved for the treatment of infection. The usual dose is 2 grams a day for 4 to 6 weeks. Side effects seen in people who have taken ceftriaxone for treatment of infection are listed below. The risks of taking ceftriaxone for more than 4 to 6 weeks are not known. Similar doses of ceftriaxone that will be given to subjects in this study were given to baboons for 26 weeks. Some of the baboons experience kidney problems and gallstones. At much higher doses of ceftriaxone than what will be given in this study, some of the baboons developed severe kidney damage, which caused kidney failure. Taking ceftriaxone for more than 6 weeks may increase your risk of kidney or gall bladder problems. We will test your blood and urine frequently to check for signs of kidney problems, and you will have abdominal ultrasounds to check for gall bladder problems.

<u>The most frequent side effects due to Ceftriaxone include:</u>

- Diarrhea
- Pain and cramping in the stomach area and
- Yeast infections in the mouth and tongue (oral candidiasis) and in women, vaginal yeast infection and itching in the vaginal area

Less frequent side effects include:

- Nausea
- Joint pain and fever
- Low white blood cell count – this may make a person at risk for certain types of bacterial infections
- Pain in the area where the drug is given
- Fever
- Allergic reaction (rash, itching, hives and difficulty breathing)
- Loss of appetite
- Blood conditions, including those that decrease your blood's ability to clot, are possible but very rare.
- Although seizures can occur with some of the antibiotics in this family of drugs, it is an unlikely event with ceftriaxone.

There may be other risks of taking ceftriaxone that have not been seen in people treated for infection. Since ceftriaxone has not been tested in people with ALS, other unexpected side effects may occur. There may be risks of drug interactions causing side effects when people are taking both ceftriaxone and riluzole.

The most serious side effects of receiving ceftriaxone are described below:

Diarrhea:

Diarrhea occurs in a small number of patients treated with ceftriaxone. You should contact your doctor right away if you develop diarrhea.

The diarrhea associated with ceftriaxone may or may not be caused by an infection. When it is not caused by an infection, it is called antibiotic-associated diarrhea. People that get antibiotic-associated diarrhea sometimes have had this in the past with other antibiotics. They usually have frequent, loose stools. It can be treated with over-the-counter medications, such as Imodium and increasing your fluid intake (water, Gatorade).

C. Difficile Diarrhea:

When the diarrhea is associated with an infection, it is usually caused by a bacteria called *C. difficile*. The symptoms of *C. difficile* diarrhea are frequent watery diarrhea, fever, nausea, loss of appetite and stomach cramping. If you have these symptoms, call **[INSERT SITE INVESTIGATOR NAME]** immediately at **[INSERT SITE INVESTIGATOR PHONE OR PAGER].** If you develop these symptoms, your doctor will check your stool for the infection. You will be treated with an antibiotic called metronidazole, or another appropriate medication, for 14 days. The diarrhea should stop after four or five days of being on the metronidazole.

If *C. difficile* diarrhea is not treated early, it can cause dehydration or more serious side effects (including pseudomembranous colitis, see below). Depending on how serious the infection is, you may have to stop the study drug for a short time or permanently.

Pseudomembranous Colitis:

The most serious side effect of ceftriaxone is pseudomembranous colitis. It is a result of an infection from *C. difficile* (see above).

The symptoms of pseudomembranous colitis are high fever (103-105°F), watery or green diarrhea and stomach cramps. If you have these symptoms, call **[INSERT SITE INVESTIGATOR NAME AND NUMBER]** IMMEDIATELY. Your doctor will admit you to the hospital for treatment. You will be treated with a different type of antibiotic and will need to be examined by a stomach/colon doctor.

Pseudomembranous colitis is a medical emergency and can be life-threatening if is not treated early. Most cases can be treated with discontinuation of the antibiotic (ceftriaxone) and treatment with one of several other medications including oral metronidazole or oral vancomycin. Pseudomembranous colitis can cause chills and rapid heartbeat, and severe cases can result in dehydration or electrolyte abnormalities. In very serious rare cases, it can cause toxic megacolon (a painful condition where the colon becomes enlarged) or colonic perforation (a hole in the colon) and can result in death.

Superinfection or Antibiotic-Resistant Infections:

Antibiotic use for a long period of time can cause the bacteria that live on a person's skin and in the body to become resistant to that antibiotic. When this happens, the antibiotic is no longer helpful in treating infections. The long term use of ceftriaxone may lead to the growth of bacteria that are resistant to ceftriaxone and other antibiotics in the same family of medicines known as cephalosporins. This means that after being on ceftriaxone for a long time, your body can get infected with bacteria that can no longer be treated by ceftriaxone, or possibly by other cephalosporins (antibiotics in the same group as ceftriaxone). This condition may last your whole life and means that any infection you develop would have to be treated with a different type of antibiotic. It is also possible that this resistance could lead to an infection that is difficult or impossible to treat.

Kidney Problems:

Occasionally treatment with cephalosporins (antibiotics in the same group as ceftriaxone) can cause kidney problems. You may develop irritation of the kidneys (acute allergic interstitial nephritis) or lack of blood supply to the kidney (acute tubular necrosis). Both of these conditions can lead to kidney failure. If you have kidney failure, you will require dialysis (mechanical process that replaces kidney function), either temporarily (if

the kidney failure resolves) or for the rest of your life (if the kidney failure is permanent). If kidney failure is untreated, it can be fatal.

Gall Bladder Problems:

The overall rate of gall bladder sludge or gallstones in STAGEs 1 and 2 of this study was 26%. Gall bladder sludge is a mixture of substances inside the gallbladder or ducts. Symptoms of gall bladder sludge or stones include pain in the abdomen, nausea, vomiting, fever and jaundice (yellowing of the skin, due to an excess substance called bilirubin in the blood). Gall bladder sludge and gallstones can be treated with medication. Sometimes, treatment of gall bladder problems requires surgery.

Data from STAGE 1 and 2 of this study, suggested that ursodiol, a medication that you will receive along with the study drug if you are selected to receive ceftriaxone, was helpful in management of these types of gall bladder problems, and was safe and well tolerated.

You will have abdominal ultrasounds performed at Week 4, Week 8, and Week 20 to look for gall bladder sludge or stones, or more frequently if you have symptoms of gall bladder disease. If you develop symptoms and gall bladder sludge or gallstones, the study drug may be stopped temporarily while you receive treatment for the gall bladder disease. You may be seen by a specialist in gall bladder disease to determine the best treatment options.

Pancreatitis:

Gallstone pancreatitis has been reported with Ceftriaxone therapy.

Pediatric Multivitamin Solution (placebo):

There have been rare reports of the following side effects associated with the multivitamin (MVI) solution that will be used as the placebo treatment:

- Rash
- Redness of the skin
- Itching
- Headache
- Dizziness
- Anxiety
- Double vision
- Hives
- Swelling around the eyes and fingers

Central Venous Catheter:

The risks of the central venous catheter include:

- Infection of skin, near the exit site of the catheter or along the catheter tube
- Infection in the blood (bacteremia) – this can sometimes cause infection of the heart or other parts of the body, which can be very serious. This may require you having the catheter removed.
- Blood clot in the vein or the catheter tube
- Blood clot in the lung
- Puncture of an artery during line placement
- Collapse of the lung (pneumothorax)
- Breakage or accidental removal of the catheter

We will give you individual instructions and written information on catheter care to prevent potential problems and to recognize catheter problems early. Consultation with your medical providers in the study will be available 24 hours a day, 7 days a week

The catheter must be cared for daily to keep it working correctly. If properly cared for, the catheter is designed to last indefinitely. Some serious problems could, however, require that it be removed. Other less serious problems may develop which can be handled with the catheter remaining in place. If you have an infection or another problem with the catheter, the catheter may have to be replaced one or more times during the study. This involves a surgical procedure to remove the original catheter and a repeat of the catheter placement procedure to implant a new catheter. If the catheter never becomes infected and there are no other problems with the catheter, it is possible that the catheter may remain in place for two years or more.

During different time periods while the catheter is in place, some risks are more likely to occur.

Early problems (during or in the weeks following catheter placement) include:

- There will be some discomfort at the surgical incision site until it heals.

- Puncture of an artery (a hole made in an artery) during placement of the catheter. Problems of puncture are usually local, in the area immediately around the puncture site; however, there is the potential for serious bleeding to occur. This risk is minimized with the use of ultrasound to guide placement of the central venous catheter.

- During the placement procedure air may get into the lung space, causing the lung to partially or completely collapse. A lung collapse may result in admission to a

hospital with the need for a chest tube and may possibly cause serious complications, including death.

- Infection of the wound where the catheter comes out of the chest. This type of infection may be controlled with oral or intravenous antibiotics. Admission to a hospital may be necessary if the infection is serious.

- As with any surgical procedure, some chest wall discomfort from the placement of the catheter is to be expected. You should let your study doctor know if this discomfort or tenderness is severe or lasts longer than expected.

The following potential risks can occur more often the longer the catheter is in place include:

Infection:

Long term placement of catheters can be complicated by the development of a local infection at the site of catheter entry (cellulitis). This can present with redness, fever, or pain. An infection can occur along the blood vessel. This is known as septic phlebitis. Both cellulitis and septic phlebitis can be associated with an infection of the bloodstream. This can cause fever, chills, a drop in blood pressure, overwhelming infection known as sepsis, and in some cases can infect the heart causing an infection known as endocarditis. In serious cases this can lead to death.

At the first sign of catheter infection (e.g. at the exit site, in the blood, etc), blood cultures are obtained and appropriate antibiotics are begun. In most cases, the catheter will need to be removed. It can be replaced when the infection has been properly treated. If you have the symptoms of an infection, you will be referred to an infectious disease doctor for evaluation.

Blood Clots:

Blood clots can occur during use of a central line. While this problem can be fixed using a medication to dissolve the clot, the risk of developing another clot increases after developing the first one.

The use of medication to dissolve the clot also increases the risk of bleeding during this clearing procedure. Blood clots inside the catheter can extend into the vein and cause a blood clot in the vein or break off to cause a blood clot in the lung or other organs. Blood clots left untreated can cause serious or life-threatening problems, and can lead to death.

If blood clots develop in the vein or go to the lung, the catheter will be removed to decrease the risk of new ones developing. You may need to take medications that thin the blood for several months. Swelling of the arm, neck, or face can result from a blood clot in the vein and may continue for several months or even longer.

Catheter Breaks:

Though the catheter is designed to last indefinitely, cracks or breaks in the catheter may occur as the catheter ages. Most often, this occurs in the tubing that extends outside the body. This is a more common problem and repair kits can permanently fix it. However, a break in the catheter increases the risk of an infection developing inside the catheter. There is also the rare potential for the air to get in the blood. If large amounts of air get into the blood stream it is an emergency that requires hospitalization.

Risks related to Catheter Removal include:

- Infection
- Bleeding or bruising in the area of the exit site or where the catheter was under your skin
- Discomfort
- Allergic reaction to the numbing medication or skin antiseptic
- Inability to remove the catheter without the addition of a new skin incision
- Air getting into the chest cavity causing a partial or complete collapse of a lung
- Breakage of the catheter resulting in part of the catheter remaining in the body or blood stream.

Drawing Blood and Placement of the Central Venous Catheter

The risks of drawing blood and placement of the Central Venous catheter include:

- Slight pain, a bruise, and/or bleeding where the needle is inserted
- Feeling faint
- Rarely, an infection may develop, which can be treated

Local Anesthetics

A small amount of medication to numb the skin for catheter placement will be used. The numbing medication is called lidocaine. You will not be able to sense pain, hot and cold for a few hours after it is injected into your skin.

Some people are allergic to lidocaine and other drugs in its class. If you are allergic to lidocaine, bupivicaine, etidocaine, mepivicaine, prilocaine or ropivicaine, you must tell the study staff before having the central venous catheter placed.

The risks of lidocaine are an allergic reaction, irritation, pain, numbness that lasts a long time, tingling and swelling at the area where it is injected.

Exposure to X-Rays (Radiation)

You will have a chest X-ray to check the placement of the central venous catheter. Additional X-rays may be done at any time if we think there is a problem with the catheter. The chest X-ray will expose you to 8 millirem (mrem) of radiation. A millirem (mrem) is the way we measure the amount of radiation. This amount of radiation is equal to about 3% of the annual background radiation one is exposed to each year from the earth and the sky.

If we use fluoroscopy to guide the placement of the central venous catheter, you will be exposed to some additional radiation, measured as 230 mrem. This additional exposure is a little over 2/3 of the amount of exposure the average person in the US gets in one year (300 mrem) from background radiation (radiation from their surroundings).

Breathing (Vital Capacity) and Muscle Strength Testing

The risks and discomforts of the tests of breathing and muscle strength include:

- Feeling tired (fatigue)
- Muscle cramps

Questionnaires

You will be asked to complete questionnaires during some of the study visits. These questionnaires ask about your quality of life and ability to carry out certain daily activities. You may feel upset when answering these questions. Although we hope you answer all of the questions, you may skip over any question you do not want to answer.

Pregnancy Risks:

If you become pregnant while taking the study drug, you should tell Dr. **[INSERT SITE INVESTIGATOR NAME]** right away. The effects of ceftriaxone on an unborn child are unknown; therefore if you become pregnant you will need to stop study drug.

If you are a woman who can become pregnant, you must not be pregnant and/or currently nursing a child. This means you must actively use effective birth control measures during the entire study. Effective birth control includes:

- Abstinence (not participating in sexual activity that may cause pregnancy)
- Hormonal Birth Control
  - Oral Birth Control Pills
  - Implanted or Injected birth control agents (Norplant, for example)
  - Other hormonal birth control (Patch or Ring, for example)
- Intrauterine Device (IUD) in place for at least 3 months before screening
- Condom AND Spermicide

- Another adequate method (as determined by steering committee member review)

The study doctor will discuss birth control methods with you before you start taking the study drug.

New Information:

We may learn new information about the study drug during the course of this study. If we do, we will share any information that might affect your decision to be in or stay in the study.

What are the possible benefits from being in this research study?

At this time it is not known if the study drug will provide any benefit to you. It is hoped that ceftriaxone will slow the rate of progression of ALS. The knowledge gained from this study may be of future benefit to you and others with ALS.

What other treatments are available?

You do not have to participate in this study to receive care for your disease. There are other clinical research trials available to patients with ALS. The study doctor can discuss any other trials open for enrollment at this time. Whether or not you choose to participate in a clinical trial will not affect your care at **[INSERT INSTITUTION NAME HERE].**

There is currently no known effective therapy for ALS. Riluzole, a drug that slows the release of glutamate, is approved by the FDA for treatment of ALS. Two studies have shown that subjects with ALS who took Riluzole lived about 3 months longer than expected. Subjects who take part in this study will be able to take Riluzole. However they must be on the same dose of Riluzole for at least 30 days before they can start the study drug.

Will I be paid to take part in this study?

You will not receive any compensation for participating in this research study.

What are the costs to me to take part in this study?

The study drug (ceftriaxone or placebo), and all study tests will be provided without cost to you or your insurance company while you are actively participating in the clinical trial. Placement of the central line, including the chest x-ray, will be provided to you without cost. *[U.S SITES USE THIS LANGUAGE]* The costs of other treatments, including care for your ALS, are the responsibility of your insurance company or you directly. *[CANADIAN SITES USE THIS LANGUAGE]* The costs of other treatments, including care for your ALS, will be covered under your provincial health care plan.

[OPTIONAL] For each study visit, your parking will be validated (or you will receive a voucher), so you will not have to pay for parking during study visits.

What happens if I get injured when taking part in this study?

If you experience any injury or illness due to your participation in this study, please contact **[INSERT SITE INVESTIGATOR NAME]** at **[INSERT SITE INVESTIGATOR PHONE NUMBER]** right away. If you need medical treatment for this injury, **[INSERT INSTITUTION NAME HERE]** will assist you in getting treatment as needed. If this is an emergency and you can not get in touch with **[INSERT SITE INVESTIGATOR NAME]** or study staff, please seek immediate medical treatment on your own. *[U.S. SITES USE THE FOLLOWING LANGUAGE]* The cost of such treatment will be billed, in the ordinary way, to you or your insurance company. Any cost over what your insurance company pays will be your responsibility. *[CANADIAN SITES USE THE FOLLOWING LANGUAGE]* If you need medical treatment for this injury [INSERT SITE NAME] will assist you in getting appropriate treatment as needed, which will be covered under your provincial health care plan.

No other compensation will be covered by the study, including compensation for lost wages, transportation, co-pays, deductibles, or indirect losses.

Who can I contact with questions on this study?

[INSERT SITE INVEISTIGATOR NAME] is the investigator in charge of this study (also called the study doctor) at **[INSERT SITE NAME]**. The study doctor can be reached at **[INSERT SITE INVESTIGATOR OFFICE #]** during the day and by page **[INSERT SITE INVESITGATOR PAGER #]**, 24 hours a day. You may also contact **[INSERT Nurse/Coordinator NAME]** at **[INSERT PHONE NUMBER]** during working hours.

If you have questions about the scheduling of appointments or study visits, call **[INSERT NAME(S)]** at **[INSERT PHONE NUMBER(S)]**.

If you want to speak with someone not directly involved in this research study, please contact the Human Research Committee [or INSERT IRB/WIRB/ERB or other research volunteer advocate] office. You can call them at [INSERT PHONE NUMBER].

You can talk to them about:

- You rights as a research subject
- Your concerns about the research
- A complaint about the research

Also, if you feel pressured to take part in this research study, or to continue with it, they want to know and can help.

CONFIDENTIALITY OF RECORDS & PERMISSION FOR RELEASE OF INFORMATION

[*NOTE: YOU MAY INSERT YOUR INSTITUTION'S SPECIFIC LANGUAGE HERE OR YOU CAN USE THE LANGUAGE INDICATED BELOW. HOWEVER YOU <u>MUST</u> LIST ALL THE ENTITIES THAT MAY HAVE ACCESS TO THE DATA AS DELINEATED IN THE BULLETS BELOW.*]

While we will make every effort to keep information we learn about you private, this cannot be guaranteed. Other people may need to see the information. While they normally protect the privacy of the information, they may not be required to do so by law. Results of the research may be presented at meetings or in publications, but your name will not be used.

The federal Health Insurance Portability and Accountability Act (HIPAA) requires us to get your permission to use health information about you that we either create or use as part of the research. This permission is called an Authorization. We will use your research record, related information from your medical records, results of DNA analysis, screening logs, case report forms and both clinical and research observations made while you take part in the research. Clinical information that will be collected includes: tests about your motor function, mood, and your activities of daily living. During the course of this study, as we collect research data, a unique code number will identify you, and all coded clinical information will be kept separately from your name.

We will use your health information to conduct the study, to monitor your health status, to measure effects of procedures, to determine research results, and possibly to develop new tests, procedures, and commercial products. Health information is used to report results of research to sponsors and federal regulators. It may be audited to make sure we are following regulations, policies and study plans. [**NAME OF INSTITUITON**] policies let you see and copy this information after the study ends, but not until the study is completed. If you have never received a copy of the [**NAME OF INSTITUTION**] HIPAA Notice, please ask the study doctor for one.

[*Note to Investigators: the Notice must be provided and receipt documented if this is the first contact with your* **[Name of Institution].]**

To meet regulations, or for reasons related to this research, the study doctor may share a copy of this consent form and records that identify you with the following people and organizations:

- U.S. National Institutes of Health (NIH)/National Institute of Neurological Disorders and Stroke (NINDS) – (the government agency funding the study)

- The NIH Data Safety Monitoring Board – (committee reviewing study conduct, progress, enrollment and safety data periodically for any safety concerns)
- The U.S. Food and Drug Administration–(the government agency oversees drug development and INDs [investigational new drug application])
- Health Canada (the Canadian government agency that oversees the patient safety issues in human research for studies conducted in Canada)
- Representatives of the Massachusetts General Hospital Neurology Clinical Trials Unit (the academic group conducting the study and the group managing the information (data) from the study)
- Representatives of the Massachusetts General Hospital Biostatistics Department, Boston, MA (the academic group responsible for the data analysis for the study)
- Representatives at The State University Of New York (SUNY) Upstate Medical University (representatives managing the outcome measures and training personnel for the study, and for monitoring study data to make sure it is complete and accurate)
- **[INSERT SITE INVESTIGATOR NAME]** and Ceftriaxone clinical trial research staff at the **[INSERT NAME OF INSTITUTION]**
- The Institutional Review Boards at the Massachusetts General Hospital and **[INSERT NAME OF INSTITUTION]** – (committees that make certain that your rights are protected)
- Representatives at ICON Laboratories (the group responsible for processing, analyzing and reporting the results of the coded blood and urine samples)
- Representatives at the Tufts University School of Medicine Laboratory – (representatives receiving your coded Ceftriaxone plasma level samples for analysis)
- Members of the Ceftriaxone clinical trial Steering Committee (the committee overseeing the progress of the trial)
- The Medical Monitor of the study - a doctor who reviews coded data on a monthly basis for safety concerns
- A doctor who specialized in kidney problems at **[INSERT NAME OF INSTITUTION]** who will be involved in the study
- A doctor who specializes in Infectious Diseases at **[INSERT NAME OF INSTITUTION]**
- The staff in the Interventional Radiology department who will place your catheter and provide medical care during the catheter placement visit
- Nurses and health care personnel at the company that will be helping you with care for your catheter at home.

If you decide to take part, your Authorization for this study will not expire unless you cancel (revoke) it. The information collected during your participation will be kept indefinitely. You can always cancel this Authorization by writing to the study doctor. If you cancel your Authorization, you will also be removed from the study. However, standard medical care and any other benefits to which you are otherwise entitled will not be affected. Canceling your Authorization only affects use and sharing of information

after the study doctor gets your written request. Information gathered **before** then may need to be used and given to others. For example, by Federal law, we must send study information to the FDA for drug and device studies it regulates. Information that may need to be reported to FDA cannot be removed from your research records.

As stated in the section on Voluntary Participation below, you can also refuse to sign this consent/Authorization and not be part of the study. You can also tell us you want to leave the study at any time without canceling the Authorization. By signing this consent form, you give us permission to use and/or share your health information as stated above.

Finally, you should understand that the study doctor is not prevented from taking steps, including reporting to authorities, to prevent serious harm to yourself or others.

Voluntary Participation:

Your participation in this research study is completely voluntary. You can decide not to participate or you can end your participation in the study at any time. Such a decision will not result in a penalty or loss of benefits to which you are otherwise entitled. In the event that you do end your participation in this study, the information you have already provided will be kept confidential.

CONSENT TO PARTICIPATE

I have read (or have had read to me) and understand the contents of this consent form and have been encouraged to ask questions. I have received answers to my questions. I agree to participate in this study. I have received (or will receive) a copy of this form for my records and future reference.

Study Subject

Print Name: ____

Signatures: ____

Date: ____

Person Obtaining Consent (Site Investigator)

I have read this form to the participant and/or the participant has read this form. An explanation of the research was given and questions from the participant were solicited and answered to the participant's satisfaction. In my judgment, the participant has demonstrated comprehension of the information.

Print Name: ____

Signatures: ____

Date: ____

CONSENT TO PARTICIPATE IN A CLINICAL RESEARCH STUDY

CAREGIVER BURDEN SUB-STUDY

<u>PROTOCOL TITLE</u>**: Clinical Trial of Ceftriaxone in Subjects with Amyotrophic Lateral Sclerosis (ALS)**

Sponsor: National Institutes of Health/National Institute of Neurological Disorders and Stroke (NIH/NINDS)

Protocol Number: CEF-ALS-2006

About this consent form

Please read this form carefully. It tells you important information about a research study. A member of our research team will also talk to you about taking part in this research study. People who agree to take part in this research study are called "subjects." This term will be used throughout this consent form. If you have any questions about the research or about this form, please ask us. If you decide to take part in this research study, you must sign this form to show that you want to take part. We will give you a copy of this form to keep.

Why is this research study being done?

The purpose of the study is to evaluate the burden that care-giving may have on you, and how that burden may change over time. You are being asked to participate in this study because you provide care to someone with ALS. Currently, very little research has focused on care providers and the personal challenges they face while providing care to patients with ALS. We hope that in collecting this data we will be better able to develop and tailor treatment regimens to benefit both patients and their caregivers.

We will offer participation in this questionnaire sub-study to all caregivers of subjects participating in the multicenter Trial of Ceftriaxone in ALS. We expect to enroll 60 subjects and caregivers in the first phase of the study, and approximately 540 of each in the second phase. Approximately 15 subjects will be enrolled at **[INSERT NAME OF INSTITUTION].** In order to find those 15 eligible subjects, we will likely screen up to 30 potential research subjects here at **[INSERT NAME OF INSTITUTION]**.

What will happen in this research study?

If you choose to participate, you will receive a questionnaire every 16 weeks throughout the course of the Ceftriaxone study, at the same time as the study visits. The survey will ask questions about your feelings towards being the provider of care for someone with ALS. The questionnaire will be identical each time you fill it out, and should take about 10 minutes to complete. Although it is hoped that you will answer all of the questions, you may skip over any questions that you do not want to answer. Your responses will not be shared with the person you care for.

What are the risks to me?

You may experience negative feelings (including sadness, frustration, anger or guilt) while reading and answering some of the questions posed in the questionnaire. There are no other risks to you from participating in this sub-study.

What are the possible benefits from being in this research study?

We do not know if taking part in this study will benefit you. However, the information learned from this study may help others in the future.

What are the alternatives?

You may choose not to participate. Your choice to participate or not will not affect the eligibility of the person you care for to participate in the main ceftriaxone study.

Will I be paid to take part in this study?

You will not receive any compensation for participating in this research study.

What are the costs to me to take part in this study?

There are no costs for participating in this study

Who can I contact with questions on this study?

For more information concerning this research or if you believe that you have suffered a research related injury, please contact: **[INSERT NAME OF SITE INVESTIGATOR],** the investigator (study doctor) at **[INSERT NAME OF INSTITUTION]**. **[INSERT NAME OF SITE INVESTIGATOR]** can be reached at **[INSERT PI CONTACT NUMBER]** during the day and by page **[INSERT PAGER NUMBER]**, 24 hours a day.

If you have questions about the rights of research participants, you may call [NAME AND PHONE NUMBER OF CONTACT PERSON FOR PARTICIPANT'S RIGHTS].

CONFIDENTIALITY OF RECORDS & PERMISSION FOR RELEASE OF INFORMATION

[NOTE: YOU MAY INSERT YOUR INSTITUTION'S SPECIFIC LANGUAGE HERE OR YOU CAN USE THE LANGUAGE INDICATED BELOW. HOWEVER YOU MUST LIST ALL THE ENTITIES THAT MAY HAVE ACCESS TO THE DATA AS DELINEATED IN THE BULLETS BELOW.**]**

While we will make every effort to keep information we learn about you private, this cannot be guaranteed. Other people may need to see the information. While they normally protect the privacy of the information, they may not be required to do so by law. Results of the research may be presented at meetings or in publications, but your name will not be used.

The federal Health Insurance Portability and Accountability Act (HIPAA) requires us to get your permission to use health information about you that we either create or use as part of the research. This permission is called an Authorization. We will use your research record, related information from your medical records, results of DNA analysis, screening logs, case report forms and both clinical and research observations made while you take part in the research. Clinical information that will be collected includes: tests about your motor function, mood, and your activities of daily living. During the course of this study, as we collect research data, a unique code number will identify you, and all coded clinical information will be kept separately from your name.

We will use your health information to conduct the study, to monitor your health status, to measure effects of procedures, to determine research results, and possibly to develop new tests, procedures, and commercial products. Health information is used to report results of research to sponsors and federal regulators. It may be audited to make sure we are following regulations, policies and study plans. **[NAME OF INSTITUTION]** policies let you see and copy this information after the study ends, but not until the study is completed. If you have never received a copy of the **[NAME OF INSTITUTION]** HIPAA Notice, please ask the study doctor for one.

[Note to Investigators: the Notice must be provided and receipt documented if this is the first contact with your institution**].**

To meet regulations or for reasons related to this research, the study doctor may share a copy of this consent form and records that identify you with the following people:

- National Institutes of Health (NIH)/National Institute of Neurological Disorders and Stroke (NINDS) – (the government agency funding the study)
- The NIH Data Safety Monitoring Board – (committee reviewing study conduct, progress, enrollment and safety data periodically for any safety concerns)
- The Food and Drug Administration– (the government agency that oversees drug development and INDs [investigational new drug applications])
- Health Canada – (the Canadian government agency that oversees patient safety issues in human research for studies conducted in Canada)
- Representatives of the Massachusetts General Hospital Neurology Clinical Trials Unit (the academic group responsible for conducting the study and for all aspects of data collection and processing)
- Representatives at The State University Of New York (SUNY) Upstate Medical University (the group managing the Outcome Measure testing and training personnel for the study, and for monitoring the study data to make sure it is complete and accurate)

- **[SITE PI]** and Ceftriaxone clinical trial research staff at the **[NAME OF INSTITUTION]**
- The Institutional Review Boards at **[NAME OF INSTITUTION] –** (committees that make certain that your rights are protected)
- Members of the Ceftriaxone clinical trial Steering Committee (the committee overseeing the progress of the trial)

If you decide to take part, your Authorization for this study will not expire unless you cancel (revoke) it. The information collected during your participation will be kept indefinitely. You can always cancel this Authorization by writing to the study doctor. If you cancel your Authorization, you will also be removed from the study. However, standard medical care and any other benefits to which you are otherwise entitled will not be affected. Canceling your Authorization only affects uses and sharing of information **after** the study doctor gets your written request. Information gathered before then may need to be used and given to others. For example, by Federal law, we must send study information to the FDA for drug and device studies it regulates. Information that may need to be reported to FDA cannot be removed from your research records.

As stated in the section on Voluntary Participation below, you can also refuse to sign this consent/Authorization and not be part of the study. You can also tell us you want to leave the study at any time without canceling the Authorization. By signing this consent form, you give us permission to use and/or share your health information as stated above.

Finally, you should understand that the study doctor is not prevented from taking steps, including reporting to authorities, to prevent serious harm to yourself or others.

Voluntary Participation

Your participation in this research study is completely voluntary. You can decide not to participate or you can end your participation in the study at any time. Such a decision will not result in a penalty or loss of benefits to which you are otherwise entitled. In the event that you do end your participation in this study, the information you have already provided will be kept confidential.

CONSENT TO PARTICIPATE

I have read (or have had read to me) and understand the contents of this consent form and have been encouraged to ask questions. I have received answers to my questions. I agree to participate in this study. I have received (or will receive) a copy of this form for my records and future reference.

Study Subject

Print Name: ______________________________

Signatures: ______________________________

Date: ______________________________

Person Obtaining Consent (Study Doctor)

I have explained the purpose of the research, the study procedures, the possible risks and discomforts and potential benefits. I have answered any questions regarding the research study to the best of my ability.

Print Name: ______________________________

Signatures: ______________________________

Date: ______________________________

Protocol Appendix 4

Questionnaires

ALS Functional Rating Scale – Revised (ALSFRS-R)

1. SPEECH

4 Normal speech processes
3 Detectable speech disturbance
2 Intelligible with repeating
1 Speech combined with non-vocal communication
0 Loss of useful speech

2. SALIVATION

4 Normal
3 Slight but definite excess of saliva in mouth; may have night-time drooling
2 Moderately excessive saliva; may have minimal drooling
1 Marked excess of saliva with some drooling
0 Marked drooling; requires constant tissue or handkerchief

3. SWALLOWING

4 Normal eating habits
3 Early eating problems – occasional choking
2 Dietary consistency changes
1 Needs supplemental tube feeding
0 NPO (exclusively parenteral or enteral feeding)

4. HANDWRITING (with dominant hand prior to ALS onset)

4 Normal
3 Slow or sloppy; all words are legible
2 Not all words are legible
1 Able to grip pen but unable to write
0 Unable to grip pen

5a. CUTTING FOOD & HANDLING UTENSILS (patients w/o gastrostomy)

4 Normal
3 Somewhat slow and clumsy, but no help needed
2 Can cut most foods, although clumsy and slow; some help needed
1 Food must be cut by someone, but can still feed slowly
0 Needs to be fed

5b. CUTTING FOOD & HANDLING UTENSILS (patients with gastrostomy)

4 Normal
3 Clumsy but able to perform all manipulations independently
2 Some help needed with closures and fasteners
1 Provides minimal assistance to caregivers
0 Unable to perform any aspect of task

ALS Functional Rating Scale – Revised (ALSFRS-R)

6. DRESSING & HYGIENE

4 Normal function
3 Independent and complete self-care with effort or decreased efficiency
2 Intermittent assistance or substitute methods
1 Needs attendant for self-care
0 Total dependence

7. TURNING IN BED & ADJUSTING BED CLOTHES

4 Normal
3 Somewhat slow and clumsy, but no help needed
2 Can turn alone or adjust sheets, but with great difficulty
1 Can initiate, but not turn or adjust sheets alone
0 Helpless

8. WALKING

4 Normal
3 Early ambulation difficulties
2 Walks with assistance (any assistive device including ankle foot orthosis – AFO)
1 Non-ambulatory functional movement only
0 No purposeful leg movement

9. CLIMBING STAIRS

4 Normal
3 Slow
2 Mild unsteadiness or fatigue
1 Needs assistance (including handrail)
0 Cannot do

RESPIRATORY SUBSCALE: (R-1 THROUGH R-3)

R-1. DYSPNEA

4 None
3 Occurs when walking
2 Occurs with one or more of the following: eating, bathing, dressing
1 Occurs at rest, difficulty breathing when either sitting or lying
0 Significant difficulty, considering using mechanical respiratory support

R-2. ORTHOPNEA

4 None
3 Some difficulty sleeping at night due to shortness of breath; does not routinely use more than two pillows
2 Needs extra pillow in order to sleep (more than two)
1 Can only sleep sitting up
0 Unable to sleep

R-3. RESPIRATORY INSUFFICIENCY

4 None
3 Intermittent use of BiPAP
2 Continuous use of BiPAP during the night
1 Continuous use of BiPAP during the night and day
0 Invasive mechanical ventilation by intubation or tracheostomy

ALS-Specific Quality of Life Questionnaire (ALSSQOL)

ALS-Specific Quality of Life Questionnaire

Subject ID# ____________________

Date: _________________________

Instructions:

The questions in this questionnaire begin with a statement followed by two opposite answers. Numbers extend from one extreme answer to its opposite. Please circle the number between 0 and 10, which is most true for you.
There are no right or wrong answers.
Completely honest answers will be most helpful.

EXAMPLE:

	**Not at All**										**Extremely**
I am hungry.	0	1	2	3	4	5	6	7	8	9	10

- If you are not even a little bit hungry, you should circle 0.
- If you are a little hungry (you just finished a meal but still have room for dessert), you might circle 1, 2, or 3
- If you are feeling moderately hungry (because mealtime is approaching), you might circle 4, 5, or 6.
- If you are very hungry (because you haven't eaten all day), you might circle a 7, 8, or 9.
- If you are extremely hungry, you should circle 10.

ALS-Specific Quality of Life Questionnaire (ALSSQOL)

BEGIN HERE

Please assess your overall quality of life over the **past week *(7 days):***

	**Very bad**										**Excellent**
Considering all parts of my life – physical, emotional, social, spiritual, and financial – over the past week, the quality of my life has been.	0	1	2	3	4	5	6	7	8	9	10

ALS-Specific Quality of Life Questionnaire (ALSSQOL)

Please rate the following symptoms and experiences according to **how much of a problem** each one has been for you. Please respond about how you have felt or what you have experienced over the **past week** using the scale provided.

		**No Problem**										**Tremendous Problem**
1.	Pain	0	1	2	3	4	5	6	7	8	9	10
2.	Fatigue	0	1	2	3	4	5	6	7	8	9	10
3.	Eating	0	1	2	3	4	5	6	7	8	9	10
4.	Excessive Saliva	0	1	2	3	4	5	6	7	8	9	10
5.	Mucous in My Throat	0	1	2	3	4	5	6	7	8	9	10
6.	Speaking	0	1	2	3	4	5	6	7	8	9	10
7.	Breathing	0	1	2	3	4	5	6	7	8	9	10
8.	My Strength and Ability to Move	0	1	2	3	4	5	6	7	8	9	10
9.	Sleep	0	1	2	3	4	5	6	7	8	9	10
10.	Bowel and Bladder (Constipation, Diarrhea, Poor Control)	0	1	2	3	4	5	6	7	8	9	10

ALS-Specific Quality of Life Questionnaire (ALSSQOL)

Please rate the following statements according to **how strongly you agree or how strongly you disagree** with each of them. Please respond about how you have felt or what you have experienced over the **past week.**

		**Strongly Disagree**										**Strongly Agree**
11.	I have felt physically terrible.	0	1	2	3	4	5	6	7	8	9	10
12.	My life has been purposeful and meaningful.	0	1	2	3	4	5	6	7	8	9	10
13.	I have been coping well with my illness.	0	1	2	3	4	5	6	7	8	9	10
14.	I believe I have control over my life.	0	1	2	3	4	5	6	7	8	9	10
15.	When I have thought about my life, I thought that my life to this point has been worthwhile.	0	1	2	3	4	5	6	7	8	9	10
16.	The past week has been a burden.	0	1	2	3	4	5	6	7	8	9	10
17.	The world has been caring and responsive to my needs.	0	1	2	3	4	5	6	7	8	9	10
18.	I have felt supported.	0	1	2	3	4	5	6	7	8	9	10
19.	ALS has interfered with the important things in my life.	0	1	2	3	4	5	6	7	8	9	10
20.	The past week has been a gift.	0	1	2	3	4	5	6	7	8	9	10
21.	I have felt good about myself as a person.	0	1	2	3	4	5	6	7	8	9	10
22.	When I have thought about my whole life, I thought that I have achieved my life's goals.	0	1	2	3	4	5	6	7	8	9	10
23.	Whatever the future holds, I know that things will be ok.	0	1	2	3	4	5	6	7	8	9	10

ALS-Specific Quality of Life Questionnaire (ALSSQOL)

Please rate the following statements according to **how much** you have felt or experienced what is described. Please respond about how you have felt or what you have experienced over the **past week.**

		**Not at All**										**Very Much**
24.	I have been depressed.	0	1	2	3	4	5	6	7	8	9	10
25.	My religion has been a source of strength or comfort to me.	0	1	2	3	4	5	6	7	8	9	10
26.	Communication has been a problem.	0	1	2	3	4	5	6	7	8	9	10
27.	My interests, desires, and goals have been the same as they were before ALS.	0	1	2	3	4	5	6	7	8	9	10
28.	When I have thought of the future, I have been afraid.	0	1	2	3	4	5	6	7	8	9	10
29.	Relationships with those closest to me have been satisfying.	0	1	2	3	4	5	6	7	8	9	10
30.	I have been interested in other people or things.	0	1	2	3	4	5	6	7	8	9	10
31.	I have found life to be satisfying.	0	1	2	3	4	5	6	7	8	9	10
32.	I have been nervous or worried.	0	1	2	3	4	5	6	7	8	9	10
33.	I consider myself to have been religious or spiritual.	0	1	2	3	4	5	6	7	8	9	10
34.	I enjoyed spending time with other people.	0	1	2	3	4	5	6	7	8	9	10
35.	I have felt helpless.	0	1	2	3	4	5	6	7	8	9	10
36.	I have felt hopeless.	0	1	2	3	4	5	6	7	8	9	10
37.	I have enjoyed the beauty of my surroundings.	0	1	2	3	4	5	6	7	8	9	10

ALS-Specific Quality of Life Questionnaire (ALSSQOL)

Please rate the following statements according to **how often** you have felt or experienced what is described. Please respond about how you have felt or what you have experienced over the **past week.**

		**Never**										**Very Often**
38.	I have felt sad.	0	1	2	3	4	5	6	7	8	9	10
39.	I have appreciated aspects of my life I had previously thought were unimportant, or to which I had previously not given much thought.	0	1	2	3	4	5	6	7	8	9	10
40.	I have felt happy.	0	1	2	3	4	5	6	7	8	9	10
41.	I have felt lonely.	0	1	2	3	4	5	6	7	8	9	10
42.	I have found that aspects of my life which I used to think were important are actually unimportant.	0	1	2	3	4	5	6	7	8	9	10
43.	I have prayed to God.	0	1	2	3	4	5	6	7	8	9	10
44.	I have laughed.	0	1	2	3	4	5	6	7	8	9	10
45.	I was excited about or looked forward to something.	0	1	2	3	4	5	6	7	8	9	10
46.	I have engaged in religious practices in my home or in my community.	0	1	2	3	4	5	6	7	8	9	10
47.	I have participated in personal and family decisions.	0	1	2	3	4	5	6	7	8	9	10

ALS-Specific Quality of Life Questionnaire (ALSSQOL)

The following statements are about **social contact** (for example, visits from family and friends). Please think about your experiences with or how you have felt about social contact in the **past week,** and use the scales provided below to respond.

48.	My desire for social contact has been strong.	**Strongly Disagree** 0	1	2	3	4	5	6	7	8	9	**Strongly Agree** 10
49.	Family and friends have visited me.	**Never** 0	1	2	3	4	5	6	7	8	9	**Very Often** 10
50.	Visits from family and friends have been satisfying. (If you have not had any visits, please leave the response section blank).	**Not at All** 0	1	2	3	4	5	6	7	8	9	**Very Much** 10

The following statements are about **emotional intimacy** (for example, sharing deep, private thoughts; feeling connected). Please think about your experiences with or how you have felt about emotional intimacy in the **past week,** and use the scales provided below to respond.

51.	My desire for emotional intimacy has been strong.	**Strongly Disagree** 0	1	2	3	4	5	6	7	8	9	**Strongly Agree** 10
52.	I have shared emotional intimacy with others.	**Never** 0	1	2	3	4	5	6	7	8	9	**Very Often** 10
53.	Emotional intimacy with others has been satisfying. (If you have not shared emotional intimacy, please leave the response section blank).	**Not at All** 0	1	2	3	4	5	6	7	8	9	**Very Much** 10

The following statements are about **physical intimacy** (for example, touching, hugging, kissing). Please think about your experiences with or how you have felt about physical intimacy in the **past week,** and use the scales provided below to respond.

54.	My desire for physical intimacy has been strong.	**Strongly Disagree** 0	1	2	3	4	5	6	7	8	9	**Strongly Agree** 10
55.	I have shared physical intimacy with others.	**Never** 0	1	2	3	4	5	6	7	8	9	**Very Often** 10
56.	Physical intimacy with others has been satisfying. (If you have not shared physical intimacy, please leave the response section blank).	**Not at All** 0	1	2	3	4	5	6	7	8	9	**Very Much** 10

ALS-Specific Quality of Life Questionnaire (ALSSQOL)

The following statements are about **sexual intercourse**. Please think about your experiences with or how you have felt about sexual intercourse in the **past week,** and use the scales provided below to respond.

57.	My desire for sexual intercourse has been strong.	**Strongly Disagree** 0	1	2	3	4	5	6	7	8	9	**Strongly Agree** 10
58.	I have shared sexual intercourse with others.	**Never** 0	1	2	3	4	5	6	7	8	9	**Very Often** 10
59.	My sexual relationship has been satisfying. (If you have not had sexual intercourse, please leave the response section blank).	**Not at All** 0	1	2	3	4	5	6	7	8	9	**Very Much** 10

END OF QUESTIONS

THANK YOU FOR TAKING THE TIME TO COMPLETE THIS QUESTIONNAIRE

Caregiver Burden Inventory

Factor 1: Time Dependence Burden

1. My care receiver needs my help to perform many daily tasks
   - 0- not at all descriptive
   - 1- minimally descriptive
   - 2- modestly descriptive
   - 3- often descriptive
   - 4- very descriptive

2. My care receiver is dependent on me
   - 0- not at all descriptive
   - 1- minimally descriptive
   - 2- modestly descriptive
   - 3- often descriptive
   - 4- very descriptive

3. I have to watch my care receiver constantly
   - 0- not at all descriptive
   - 1- minimally descriptive
   - 2- modestly descriptive
   - 3- often descriptive
   - 4- very descriptive

4. I have to help my care receiver with many basic functions
   - 0- not at all descriptive
   - 1- minimally descriptive
   - 2- modestly descriptive
   - 3- often descriptive
   - 4- very descriptive

5. I don’t have a minute’s break from my caregiving chores
   - 0- not at all descriptive
   - 1- minimally descriptive
   - 2- modestly descriptive
   - 3- often descriptive
   - 4- very descriptive

Factor 2: Developmental Burden

1. I feel that I am missing out on life
   - 0- not at all descriptive
   - 1- minimally descriptive
   - 2- modestly descriptive
   - 3- often descriptive
   - 4- very descriptive

Caregiver Burden Inventory

2. I wish I could escape from this situation
   0- not at all descriptive
   1- minimally descriptive
   2- modestly descriptive
   3- often descriptive
   4- very descriptive

3. My social life has suffered
   0- not at all descriptive
   1- minimally descriptive
   2- modestly descriptive
   3- often descriptive
   4- very descriptive

4. I feel emotionally drained due to caring for my care receiver
   0- not at all descriptive
   1- minimally descriptive
   2- modestly descriptive
   3- often descriptive
   4- very descriptive

5. I expected that things would be different at this point in my life
   0- not at all descriptive
   1- minimally descriptive
   2- modestly descriptive
   3- often descriptive
   4- very descriptive

Factor 3: Physical Image Burden

1. I'm not getting enough sleep
   0- not at all descriptive
   1- minimally descriptive
   2- modestly descriptive
   3- often descriptive
   4- very descriptive

2. My health has suffered
   0- not at all descriptive
   1- minimally descriptive
   2- modestly descriptive
   3- often descriptive
   4- very descriptive

Caregiver Burden Inventory

3. Caregiving has made my physically sick
    - 0- not at all descriptive
    - 1- minimally descriptive
    - 2- modestly descriptive
    - 3- often descriptive
    - 4- very descriptive

4. I'm physically tired
    - 0- not at all descriptive
    - 1- minimally descriptive
    - 2- modestly descriptive
    - 3- often descriptive
    - 4- very descriptive

Factor 4: Social Burden

1. I don't get along with other family members as well as I used to
    - 0- not at all descriptive
    - 1- minimally descriptive
    - 2- modestly descriptive
    - 3- often descriptive
    - 4- very descriptive

2. My caregiving efforts aren't appreciated by others in my family
    - 0- not at all descriptive
    - 1- minimally descriptive
    - 2- modestly descriptive
    - 3- often descriptive
    - 4- very descriptive

3. I've had problems with my marriage
    - 0- not at all descriptive
    - 1- minimally descriptive
    - 2- modestly descriptive
    - 3- often descriptive
    - 4- very descriptive

4. I don't do as good a job at work as I used to
    - 0- not at all descriptive
    - 1- minimally descriptive
    - 2- modestly descriptive
    - 3- often descriptive
    - 4- very descriptive

Caregiver Burden Inventory

5. I feel resentful of other relatives who could but do not help
   0- not at all descriptive
   1- minimally descriptive
   2- modestly descriptive
   3- often descriptive
   4- very descriptive

Factor 5: Emotional Burden

1. I feel embarrassed over my care receiver's behavior
   0- not at all descriptive
   1- minimally descriptive
   2- modestly descriptive
   3- often descriptive
   4- very descriptive

2. I feel ashamed of my care receiver
   0- not at all descriptive
   1- minimally descriptive
   2- modestly descriptive
   3- often descriptive
   4- very descriptive

3. I resent my care receiver
   0- not at all descriptive
   1- minimally descriptive
   2- modestly descriptive
   3- often descriptive
   4- very descriptive

4. I feel uncomfortable when I have friends over
   0- not at all descriptive
   1- minimally descriptive
   2- modestly descriptive
   3- often descriptive
   4- very descriptive

5. I feel angry about my interactions with my care receiver
   0- not at all descriptive
   1- minimally descriptive
   2- modestly descriptive
   3- often descriptive
   4- very descriptive
