# Supplementary material for: Design and Initial Results of a Multi-Phase Randomized Trial of Ceftriaxone in Amyotrophic Lateral Sclerosis
Source: PLoS One. 2013 Apr 17;8(4):e61177. doi: 10.1371/journal.pone.0061177 (PMC3629222; doi:10.1371/journal.pone.0061177)
Supplement: File S1 — Author Appendix - Northeast ALS Consortium (NEALS) authors. (DOC) [file pone.0061177.s003.doc]

Northeast ALS Consortium (NEALS) Author Appendix

Massachusetts General Hospital

Thomas Kwiatkowski, MD

Darlene Sawicki

Allison Goldenberg

Jennifer Scalia

Pat Andres, MS, PT, CRA

Matt Bellanich, NP

SUNY Upstate

James Taft, MPAS, PA-C

Mary Lou Watson, RRT

Katherine Tindall, CCRP

Laura Simionescu, MD

Megan Grosso, RPA-C, DPT

Washington University

Oliver Ni, MD

Julaine Florence, PT, DPT

Barbara Abrams

Betsy Malkus, PT

Jeanine Schierbecker, PT

Renee Renna

Stacy Sketon RN MSN, CCRC

Charlie Wulf

Theresa Radake, RN, CCRC

Wake Forest University

Michael S. Cartwright, MD

Mozhdeh Marandi, MD

Theresa Johnston-Crews, RN

Barbara Freiberg, PT

Carolyn Ashburn, RN

California Pacific Medical Center

Catherine Madison, MD

Barbara Russell, RN/MS

Dina Scholtz, MD, CCRC

Terence Santos

Giovanna Kushner

Michelle Mendoza, PT

Marguerite Engel

Methodist Neurological Institute

Milvia Pleitez, MD

Sharon Gilson, RN

Joan Appel, PA-C, CCRC

Peggy Ingels Allred, PT

Kaye Wilson

Valrie Bickley

Luis Lay

Sharon Halton

Carolinas Medical Center

Elena Bravver, MD

Sanjay Iyer, MD

Anne Blythe, RN

Lien Ngo

Cathleen Stenger

Sheryl Homes MD

Indiana University

John Kincaid, MD

Angela Hensley Micheels, PT

Sandra Guingrich, LPN, CCRC

Emory University

Jonathan Glass, MD

Meraida Polak, RN

Crystal Richards

Jaffar Khan, MD

University of Chicago

Elizabeth Shaviers, CCRP

Joumana Fawaz-Baroody RN

Betty Soliven, MD

Steering Committee:

Swati Aggarwal, MD

Benjamin Brooks, MD

Robin Conwit, MD

Donna Felsenstein, MD

Carl Leventhal, MD

Robert Miller, MD

Jeffrey Rothstein, MD PhD

Nina Tolkoff-Rubin, MD

Robert Rubin, MD

David Schoenfeld, PhD

Myles Keroack, MD

Stephan Wicky, MD

David Greenblatt, MD

Jeffrey Rosenfeld, PhD, MD, FAAN

Coordination Center:

Matt Bellanich, NP

Alicia Di Bernardo, MD

John Vetrano, PharmD

Cheryl Reilly-Tremblay, RPh

Amy Swartz, PT, DPT

Kathryn Delaney

Francesca Belouin

Marianne Kearney

Brenda Thornell

Sarah Titus, MPH

Systems and Data Management:

Alexander Sherman

Hong Yu, MS

Mabel Chan, MPH

Veena Lanka, MBBS MPH

April Opoliner

Statistics:

Amy Shui

Hui Zhang

Yuvika Paliwal

Pharamacokinetics:

Jerold S. Harmatz

Yanli Zhao
